# Identifying healthy and sustainable high-impact eating behaviour in French children aged 6–15 years: a combined multidisciplinary and living lab participatory approach

**DOI:** 10.1017/jns.2026.10105

**Published:** 2026-05-26

**Authors:** Anthony Fardet, Claire Planchat-Héry, Edmond Rock, Laureen Bochan

**Affiliations:** 1 INRA, UMR 1019, UNH, CRNH Auvergne, F-63000 Clermont-Ferrand & Clermont Auvergne Université, Université d’Auvergne, Unité de Nutrition Humaine, BP 10448, F-63000 Clermont-Ferrand, France; 2 Department of Consumer Research, Communication, and Food Sociology, Justus-Liebig University, 35390 Giessen, Germany

**Keywords:** Children, Eating behaviours, Health, Living lab, Sustainability

## Abstract

A growing standardisation of eating behaviours worldwide is observed, especially in children, notably characterised by the increasing consumption of both unsustainable animal- and plant-based ultra-processed foods (UPFs). The objective of this study was to identify five high-impact eating behaviours (HIBs) in a French child population (living in Clermont-Ferrand city and surroundings, *n* = 92, aged 6–15), that is, behaviours to change for the strongest positive impact on health and sustainability. For this, both multidisciplinary top-down and participatory bottom-up approaches has been carried out. First, from European and French dietary guidelines, desk research, European researchers’ evaluation, and expert interviews and workshops the technical potential (for their impact on health and environment), plasticity and feasibility of 40 HIB were first quantified (top-down approach). Secondly, in the French living lab (LL), from photo-voice and focus groups with children, online surveys, meetings with families and children, interviews with stakeholders, and assessments of the behavioural change potential of the French LL children’s target, five HIB were finally selected (bottom-up approach). Therefore, among the initial 342 European recommended eating behaviours, the selected HIBs for our specific population were: limiting the consumption of UPFs (notably those high in added salt, sugars and/or fats), eating 0–3 servings of meat/week, eating three servings of legumes/week, choosing primarily wholegrain than refined cereals, and choose water instead of sweetened beverages. These five HIBs constitutes a solid basis to perform next LL participatory actions, notably to test HIB feasibility/plasticity, and implement them in real life conditions through transversal actions with non-researcher.

## Graphical abstract

**Figure:**
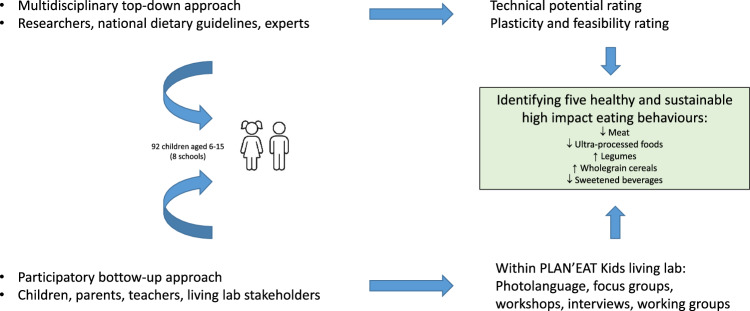

## Introduction

A growing standardisation of eating behaviours worldwide is being observed, beginning in high-income countries, and now extending to low- and middle-income countries.^(1)^ This tendency is notably characterised by the increasing consumption of animal products^(2)^ and ultra-processed food (UPF),^(3)^ resulting in monotonous and unsustainable diets that include high levels of empty calories.^(4)^ Thus, UPFs are hyper standardised industrial products that are composed of similar ingredients, regardless of the country,^(5)^ and therefore threatening culinary traditions among the youngest.^(6)^ The scale of economy applied to these products demands a high availability of standardised ingredient quality throughout the year, regardless of the season considered, driving unsustainable food systems.^(6,7)^ Such standardisation of eating behaviours has high hidden costs for human health, the environment, and socio-economics.^(8)^ Therefore, improving eating behaviours toward healthier and more sustainable choices appears to be a relevant lever for global health, especially if it is adopted by children, who can potentially maintain their eating behaviours throughout their life.

Children’s eating behaviours are influenced at the macro, meso- and micro levels. At the macro level, the dominant food system is mainly hyper standardised and globalised,^(9)^ which does not always allow the supply of high-quality foods in line with their culinary traditions. International (*e.g.* WHO)^(10)^ and national dietary guidelines (*e.g.* French PNNS, Programme National Nutrition Santé, and PNAN, Programme National de l’Alimentation et de la Nutrition)^(11)^ struggle to reach their targets and improve eating behaviours. Although these guidelines overall recommend healthy foods, they reflect a too marked top-down approach,^(12)^ *i.e.* they are very closely linked to social norms, not very flexible, not always adapted to the specific needs of regional populations and the ecological availability of food resources, and are difficult to follow most of the time by the most disadvantaged households with children.^(13)^ There is also high pressure at the meso level that influences children’s eating behaviours to be unhealthy and unsustainable, such as through food advertising on all forms of media,^(14)^ food offered in the school canteen,^(15)^ the establishment of fast-food restaurants around school locations,^(16)^ and family food purchases (*e.g.* for breakfast, snacks, and dinner, when families have insufficient time to cook).^(17,18)^ At the micro level, the socio-economic conditions of families, preferred tastes, eating pleasure, lifestyles driving convenient food demand, food culture, culinary traditions, religious food prohibitions, and/or children’s psychological state (*e.g.* vulnerability, poor self-esteem) are generally insufficient to resist meso- and macro-level factors, as they are more difficult to change.^(19)^ For example, children between 6 and 15 years of age are preferred targets of food advertising and marketing, generally for unhealthy UPFs,^(20)^ which are often highly consumed at breakfast and snack times.^(21)^

To study children^(22)^ and adolescent^(23)^ eating behaviours in real life conditions, and to consider their sociocultural context, the living lab (LL) approach appears as a relevant method. A LL is defined as an open, user-centred innovation ecosystem based on a systematic co-creation approach, integrating research and innovation processes into real communities and environments,^(24)^ and is therefore both empirical, holistic, bottom-up, and participatory. Within a LL, researchers collect their data ‘*in an existing eating environment*’,^(25)^ considering children in association with six categories of stakeholders to potentially work together to improve children’s eating behaviours, *i.e.* education and health professionals, agri-food chain actors, civil society (*e.g.* NGOs, associations, and citizens), politics, and local researchers. Indeed, interrelationships of children with their immediate environment is unique, and influenced by many specific interactions with parents, teachers, health professionals, among others.^(26)^ Notably, interactive actions with children in real life conditions (*e.g.* workshops, focus group, photo language…) within a LL context ‘*can help explore children’s cognitive thinking about context and future perspectives*’.^(22)^ Therefore, also investigating eating behaviours through the lens of children’s eyes allows for better development of scientific-derived innovative solutions to reach significant behavioural changes.

Unsustainable food systems generate relevant hidden costs at health, environmental and social levels. For example, unhealthy dietary patterns related to non-communicable diseases account for 70% of all quantified hidden costs^(27)^, among which overweight and obesity. Thus, in France, 17% of children (<18 years old) were overweight in 2014, whereas 4–5% were obese.^(28)^ In 2020, overweight affects 34% of children aged 2 to 7 and 21% of young people aged 8 to 17, while obesity affects 18% and 6%, respectively, reflecting a sharp increase in only six years.^(29)^ In our region, a study carried out in 2009 among elementary school students revealed that 11.4% of children were affected by overweight, and 3.3% by obesity.^(30)^

Therefore, the main objective of this study was to identify and select five high-impact eating behaviours (HIBs) in our regional LL children population, and that simultaneously benefit human health, the environment, and social factors. For this, with regards to the specific target group of children and involved stakeholders, potential eating behavioural changes have notably been measured *via* a tripartite framework, and comprising three key components: technical potential, initiative feasibility, and behavioural plasticity.^(31)^ Such HIBs can be different in other contexts, countries, and populations of the eight other European LLs of the PLAN’EAT project in which the same research was conducted. For this, both participatory bottom-up and multidisciplinary top-down evaluations have been performed, and combined to reach the most relevant HIB selection.

## Methods

### Study setting

This study was part of a wider project, *Food Systems Transformation toward Healthy and Sustainable Eating behaviour* (work program HORIZON-CL6-2021-FARM2FORK-01-15), coordinated by the Council for Agricultural Research and Economics (CREA, Italy). The PLAN’EAT project aims to transform food systems and environments through healthier and more sustainable eating behaviours. The French PLAN’EAT Kids-INRAE LL is a partner in a consortium of 24 organisations in 11 European countries (see: https://planeat-project.eu/).

**Figure 1.**
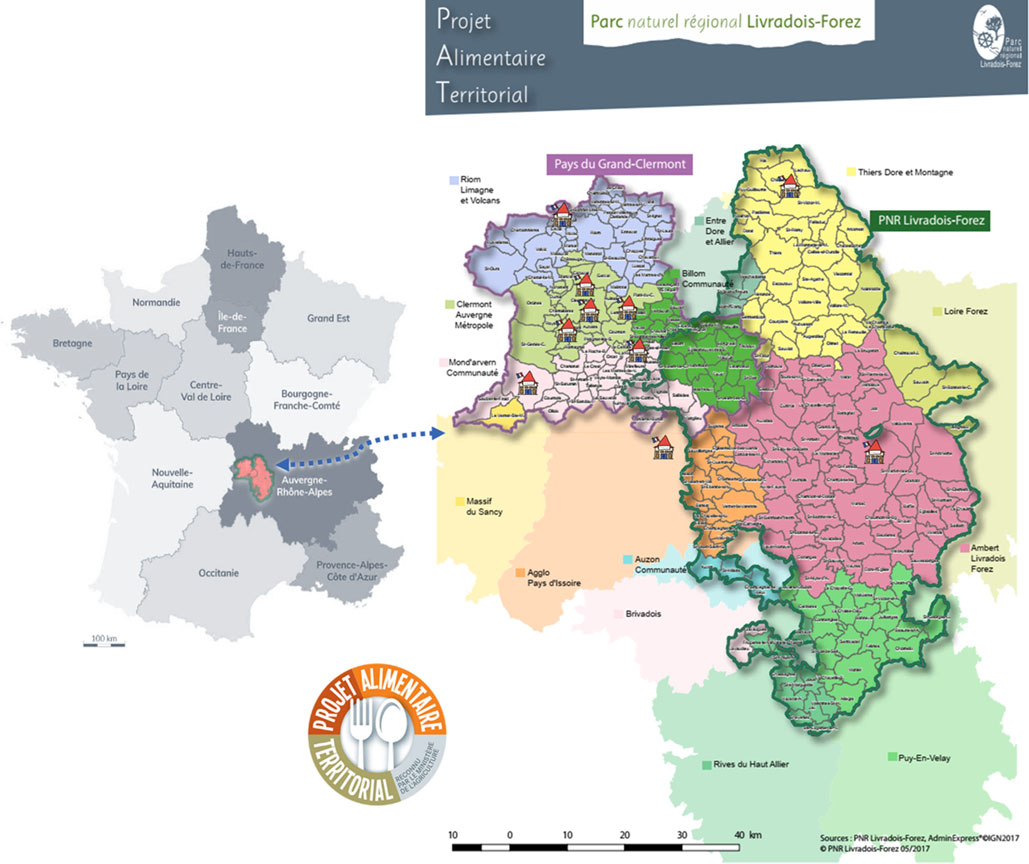
The geographical area of the French Territorial Food Project (PAT Grand Clermond/Parc Naturel Regional Livradois-Forez, PNR) that hosts the French PLAN’EAT Kids-INRAE living laboratory (LL). Selected schools were indicated with symbols in both urban, peri-urban and rural areas. PNR Livradois-Forez correspond to exclusively rural areas (on the right-hand side of the figure).

To understand the issue of unhealthy and unsustainable food behaviours at micro, meso and macro levels, we implemented a LL (*i.e.* PLAN’EAT Kids-INRAE LL) at the centre of France. The users targeted are children in primary, middle and high schools between 6 and 15 years old, *i.e.* the time during which food behaviours and tastes are gradually built (from this point, they tend to persist into adulthood). The LL includes 227 children (44% girls, 40% boys, and 16% unspecified), who are apparently healthy and live in the territory of both Clermont-Ferrand city, an urban agglomeration and its surroundings called Pays du Grand Clermont, and in rural areas linked with the National Regional Park Livradois-Forez. These children come from rural, peri-urban and urban schools and from families with different socio-economic statuses. Socio-economic statuses have been initially collected through a previous PLAN’EAT survey requesting families’ incomes (varying from <1,500 to >7,001 euros/month, results not shown). In the period where we were defining and selecting the HIBs, our panel included 92 children, essentially from urban and peri-urban areas, but with different socio-economic statuses. The unique feature of these two areas is that they are united around a food policy known as the Territorial Food Project (PAT, Projet Alimentaire Territorial, in French) (Figure 1).

Beyond the LL children, this study also mobilised other European project partners and external experts, as well as two INRAE LL scientific coordinators. The two INRAE researchers were specialised in healthy and sustainable diets, and in participatory research. Initially, for HIB selection, Justus-Liebig University’s researchers, including a nutritionist, an environmental psychologist, and an economist, all specialising in consumer research, identified and selected dietary behaviours with high impact potential, *i.e.* behaviours 1) that show a significant behaviour change potential; 2) whose changes would be acceptable for target groups and scalable; and 3) lie within a safe operating space for food systems encompassing environmental, social and health impacts. To do so, in each European LL (*n* = 9; located in Ireland, Hungary, Spain, Greece, Italy, Germany, Poland, Sweden, France), Justus-Liebig University’s researchers, in collaboration with all LL coordinators, implemented behavioural scoping, mapping and behavioural reduction. Scoping and mapping were realised by employing desk research and expert interviews, and behavioural reduction was carried out during a final workshop. The interviews and workshop took place with one representative per LL who knows well the considered target group. Up to five behaviours has been finally selected per LL and population group to be further analysed, with regard to identify potential leverage points for intervention strategies to induce behaviour change. Concerning the experts, the three impact areas (*i.e.* environmental, health, and socio-economic) were surveyed separately. Experts were recruited anonymously from within the institutions of the PLAN’EAT research consortium (*n* = 8) and from external institutions (*n* = 3) recommended by consortium partners based on existing collaborations and recognised expertise in the respective impact areas. Each expert provided responses only for their area of specialisation. The present study reports the procedure (see below) and results for the French LL.

### Ethical approval

This study was conducted according to the guidelines laid down in the Declaration of Helsinki and all procedures involving research study participants were approved by the Research Ethics Committee of Clermont Auvergne University (reference IRB00011540-2022-97). Besides, written informed consents were obtained from all children and parents.

### HIB identification procedure

**Figure 2.**
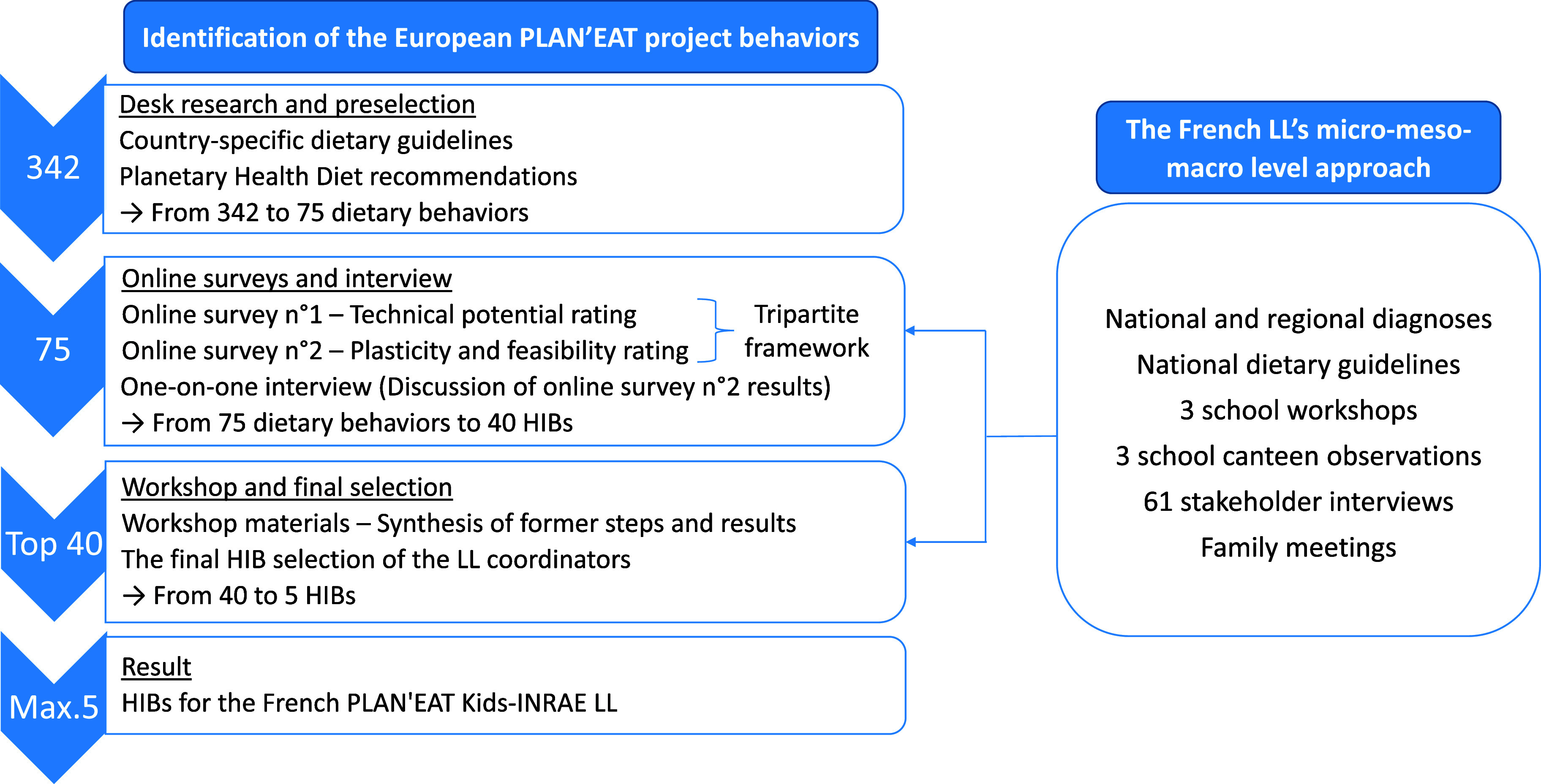
Chronological overview of the approaches to select five HIBs for the French living laboratory.

From February to July 2023, several quantitative and qualitative steps were carried out to identify and select five HIBs for the French PLAN’EAT Kids-INRAE LL (Figure 2). These steps are described below in chronological order:

- *Step 1: From 342 to 75 dietary behaviors (desk research and European researcher’s preselection)*

Initially, a desk research was carried out to preselect potential behaviours based on national dietary guidelines from European countries participating in the PLAN’EAT project, *i.e.* the ‘Programme National Nutrition Santé n°4” (PNNS4) for the French PLAN’EAT Kids-INRAE LL, as well as the Planetary Health Diet recommendations,^(32)^ resulting in a collection of 342 recommended eating behaviours for the 9 LLs (Figure 2 and SM 1, ‘Dietary Behaviors Selection – Country overview’ tab). The Planetary Health Diet recommendations were included to add dietary behaviours relevant to the objectives of the PLAN’EAT project, *i.e.* sustainable and healthy diets.

Justus-Liebig University’s researchers systematically reduced the preselected behaviours to 75 key behaviour items in 12 different categories, *e.g.* by deleting overlapping behaviours, and choosing behaviours most in alignment with the aim of developing sustainable and healthy diets (SM 2, ‘Dietary Behavior Selection – Survey structure’ tab).

- *Step 2: From 75 dietary behaviors to 40 HIBs (online surveys and interviews)*

In a second step (Figure 2), the 75 proposed key behaviours were evaluated through two types of online surveys developed by PLAN’EAT researchers using Qualtrics CoreXM software (Qualtrics^©^, Seattle, WA, USA) to interrogate the components of the tripartite framework:^(31)^

Online survey n°1 (Technical potential rating): PLAN’EAT experts assessed technical potential of key behaviours in terms of their environmental, health, and socio-economic impacts. Technical potential refers to the maximum possible benefits that could be achieved if specific behavioural changes were fully realised. For each of the 75 key behaviours, a technical potential sum score was calculated by summing the environmental and health ratings averaged across project and external experts.^(31,33)^ The three impact areas were surveyed individually, *i.e.* experts with specialised expertise in each impact area provided responses only for their area of expertise.

The environmental and health sub-dimensions each consisted of the same set of 80 items and were assessed using a 5-point Likert scale ranging from 0 (‘No/negative potential’) to 5 (‘Very high potential’). The environmental survey (SM 3) was completed three times and the health survey (SM 4) six times; mean scores were first calculated for each sub-dimension, and subsequently summed, resulting in a total technical potential score ranging from 0 to 10.

The socio-economic dimension (SM 5) was assessed using the same 80 items but was completed only twice. In addition, 9 items were rated as ‘Not applicable/Don’t know’ in the socio-economic survey, whereas all items were rated in the environmental and health surveys. Based on expert feedback, the limited number of responses, and the presence of missing values, socio-economic ratings were not included in the technical potential sum score, but were analysed separately, and used as a control. Specifically, for all behaviours classified as having high technical potential (sum score 6.0–10), socio-economic scores were checked to ensure they were not lower than 2.5 (on a 0–5 scale), in order to avoid selecting behaviours with low socio-economic potential.

**Figure 3.**
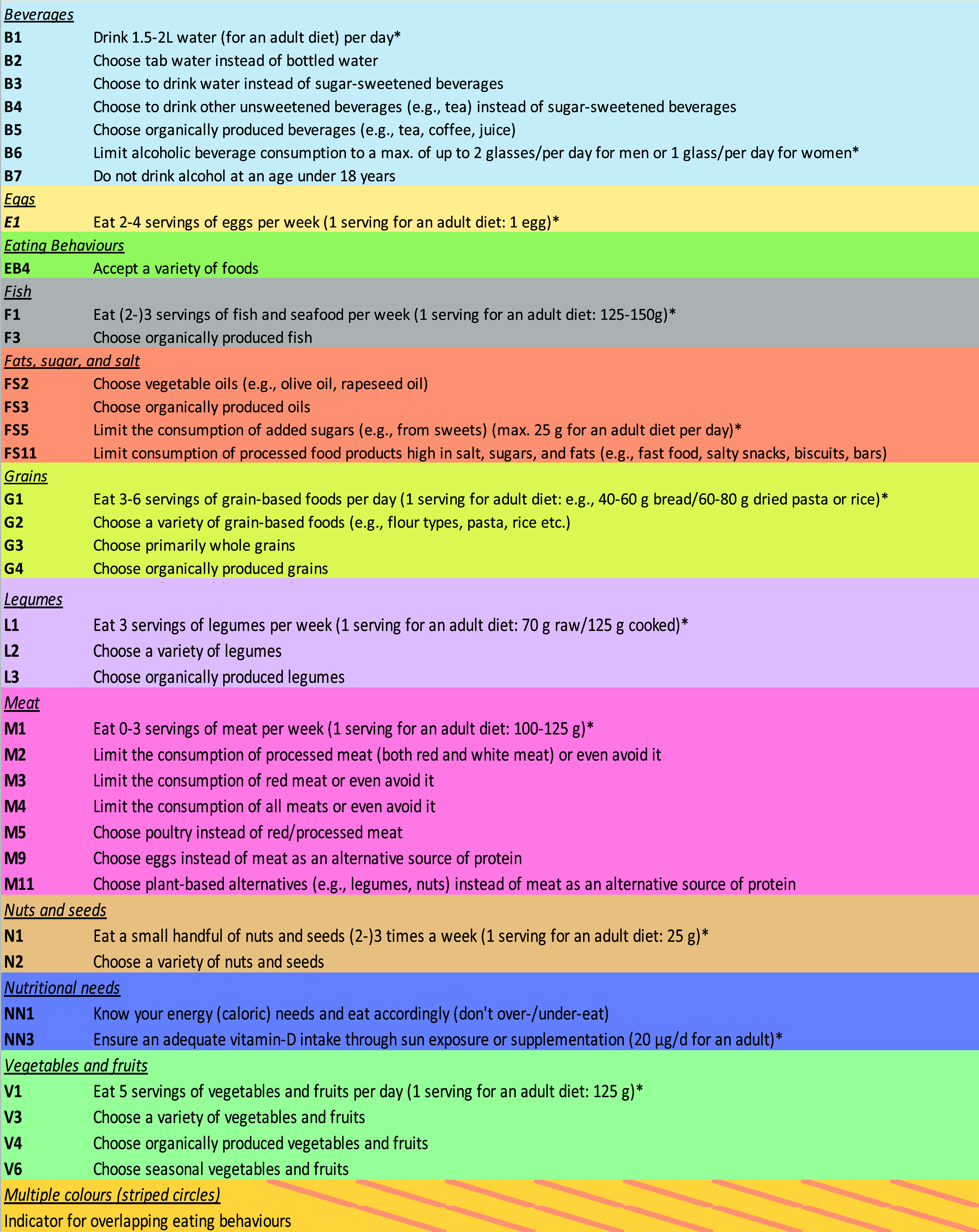
The top 40 high-impact behaviours (*i.e.* HIBs).

The environmental, health, and socio-economic impact potential was assessed with questions such as: ‘*What is the environmental (or health or socioeconomic) potential of the following behaviours related to red meat? e.g. ‘Limit the consumption of red meat or even avoid it*’. Thresholds were defined based on the midpoint of the technical potential scale: sum scores of 0–5.9 were classified as no/low technical potential for HIBs, whereas sum scores of 6.0–10 were classified as high technical potential. Using this score, the 75 key behaviour items were reduced to the top 40 HIBs in 11 categories, such as ‘beverages’, ‘legumes’, and ‘meat’ (Figures 2 and 3), *i.e.* eating behaviours that were chosen to improve environmental and health outcomes while considering socio-economic aspects.

Online survey n°2 (Plasticity and Feasibility rating): PLAN’EAT LL coordinators assessed behavioural plasticity and initiative feasibility of the key behaviours. Behavioural plasticity refers to the degree to which children can adopt a behavioural shift,^(33,34)^ and thus the likelihood that individuals will respond to initiatives in ways that lead to meaningful behavioural change, taking into account social and cultural influences. Initiative feasibility evaluates the practicality of implementing these changes by assessing whether relevant stakeholders and institutions are able and willing to support the desired change.

The survey (SM 6) consisted of two parts: the first part assessed behavioural plasticity of dietary behaviours in the target group, and the second part assessed initiative feasibility, *i.e.* the extent to which relevant stakeholders could support these behaviours. Each part contained 73 items and was answered for three different contexts: the school restaurant, and middle-lower and upper-class families. This approach aims to cover local settings in which children repeatedly spend time during food intake and to account for the different socio-economic statuses within the target group.

Behavioural plasticity was assessed using the statement ‘*How likely is it that your target group would adopt the following behaviors related to…*’, for each of the 75 behaviours, *e.g. ‘…meat: Limit the consumption of red meat or even avoid it*’ (SM 6).^(26)^ Response categories ranged from 0 (‘Target group cannot do this’) to 5 (‘Very likely’). Initiative feasibility was assessed using the statement ‘*How likely is it that relevant stakeholders would support your target group with adopting the following behaviors related to…*’, for each of the 75 behaviours, *e.g.* ‘*… meat: Limit the consumption of red meat or even avoid it*’ (SM 6) with response categories ranging from 0 (‘Stakeholders can’t support this’) to 5 (‘Very likely’).^(31)^

Based on the integer response categories of the 5-point Likert scale, thresholds were defined to distinguish between low and high potential for change. Ratings of ≤2 (‘Very unlikely’ to ‘Unlikely’) were classified as low/unlikely, whereas ratings of ≥3 (‘Somewhat likely’ to ‘Very likely’) were classified as high/likely. This cut-off reflects the point at which adoption or support begins to be likely.

In the French LL, the survey was completed once by the LL coordinator with expertise in the field, who assessed behavioural change potential for the three contexts. In the other eight LLs of the PLAN’EAT project, the same survey was completed by the respective LL coordinators for their local contexts.

The results for behavioural plasticity and initiative feasibility of the LL surveys (online survey n°2) were critically examined by Justus-Liebig University’s researchers and discussed in a subsequent one-on-one interview with the LL coordinators to obtain qualitative information about the target group, the different contexts, and the stakeholders involved (SM 7). During the interviews, examples of eating behaviour in specific contexts, such as school cafeterias, were given, and attention was given to particular cultural aspects, such as the typical eating habits of French children, especially in Clermont-Ferrand agglomeration and surroundings. The aim was to gain a better understanding of each LL, to elucidate which dietary behaviours are particularly relevant to each target group, and to identify which of these behaviours the LL should work with in the further course of the project. For the French LL, the interview content was notably based on the following research actions carried out with children and members of the LL from February to June 2023: photo-voice and focus groups, workshops and online surveys, creative food workshops, meetings with families and 92 children from 8 schools, interviews with stakeholders and members of the LL, and assessments of the behavioural change potential of the French LL children’s target. A focus was also placed on the traditions and cultural aspects of French diets. Thus, the LL leader has used their knowledge from working directly with children to answer the questions.

- *Step 3: From 40 to 5 HIBs (workshop and final selection)*

During the workshop (Figure 2), a final selection of a maximum of five HIBs per LL was realised. This was based on a synthesis of all methodological steps described above and the resulting findings. In the workshop, graphs of the synthesised survey material of the top 40 HIBs, *i.e.* the behaviours with the highest technical potential, as well as the suggestions of five HIBs proposed by Justus-Liebig University researchers based on the interviews were provided to the LL coordinators. Carrying out the workshop tasks on the synthesised results and discussing them with the entire working group was the last step for the final selection of the five HIBs for the French LL target group (SM 8). For this selection, it was important to consider the national French context. Thus, in France, the most recent dietary guidelines for children and adolescents (4–17 years old, 2019–2023) were also examined.^(35)^

- *Step 4: Final selection of the five HIB*

At the end of the workshop, the LL coordinators also had the opportunity to work on, and discuss the synthesised results of the top 40 HIBs and suggested five HIBs with representatives of other target groups to identify commonalities and differences, and further reflect on their final selection of HIBs (Figure 2). These are the eating behaviours that the French LL will continue to work on within the PLAN’EAT project, and for which intervention strategies for behavioural change will be implemented for their target group in the further course of the project. Therefore, the final five HIBs selected by the French LL may differ from the five HIBs originally proposed based on the expert interviews (SM 8), as they incorporate the LL’s experience, feedback, and field data.

## Results

### Technical potential, acceptability, and feasibility of selected eating behaviours

**Figure 4.**
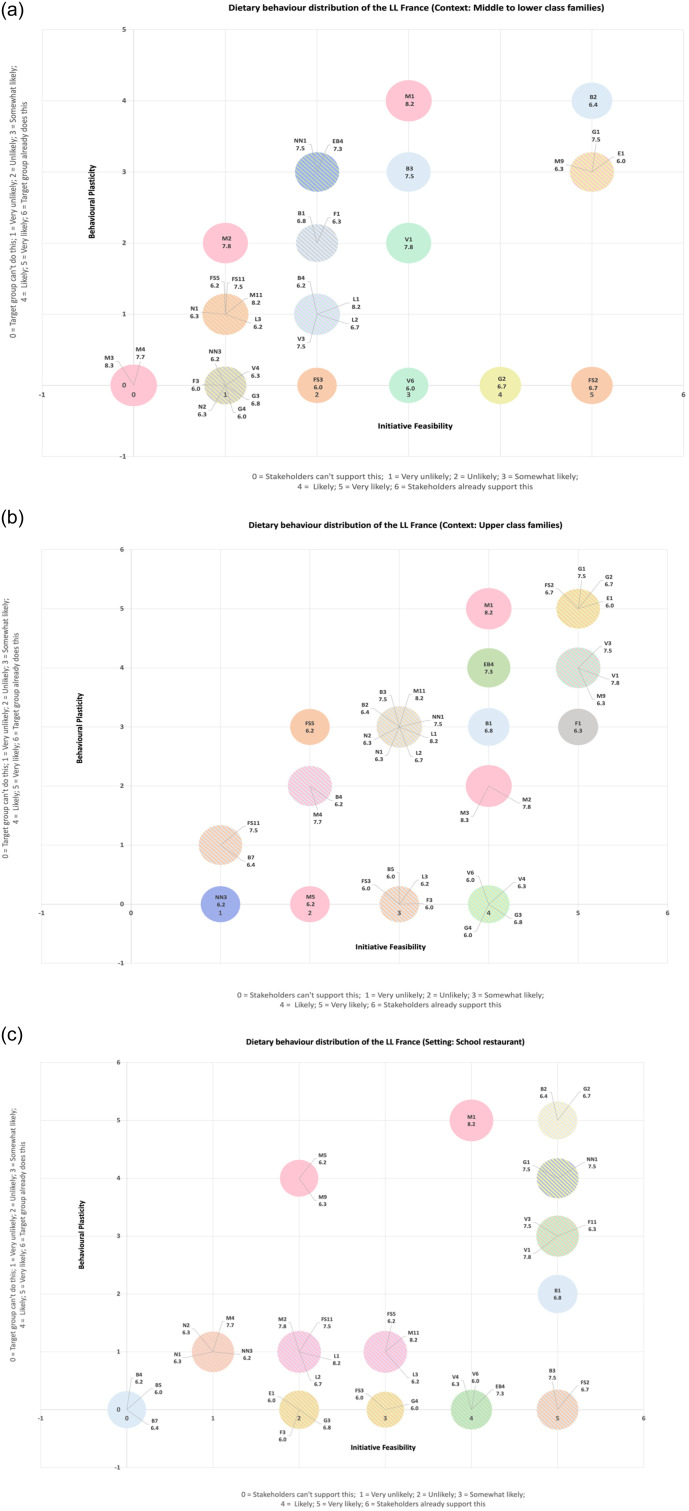
Dietary behaviour distribution of the French living laboratory: behavioural plasticity and initiative feasibility assessment in relation to the technical potential for the top 40 high-impact behaviours (HIB) (sum score ≥ 6.0) for **a)** middle-lower class families, **b)** upper-class families), and **c)** school restaurants. The numbers below the behaviour code (*e.g.*, B1) refer to the behaviour’s environmental and health potential score (*e.g.*, 6.8, see Table 1**)**; the score range is 0 to 10; the circle size is in accordance with the score (*i.e.* behaviours with higher scores have larger circles). The colour and behaviour coding on Figure 4 are the same as those on Figure 3. For example, on Figure 4a the NN1 code correspond to ‘Know your energy (caloric) needs and eat accordingly (don’t over-/under-eat)’.

Table 1 lists the top 40 HIBs in terms of their technical potential from high to low, and Figure 3 presents the colours and codes of the circles for Figures 4a–c. The synthesis of material from the methodological steps carried out, *i.e.* the results of expert and LL coordinator surveys, led to the integration of the results in one graph and for three different contexts (Figures 4a–c). Note that if the LL leaders answered ‘Not applicable/I don’t know’ for behavioural plasticity or initiative feasibility, the eating behaviour is not indicated in the graphs, which is why <40 HIBs are indicated in Table 1, Figure 3, and Figures 4a–c.

**Table 1.** Ranking of the top 40 high-impact behaviours as regards technical potential, from high to low (*i.e.* ≥6.0)

Technical potential	The top 40 HIBs
8.3	Limit the consumption of red meat or even avoid it
8.2	Eat 0–3 servings of meat per week (1 serving for an adult diet: 100–125 g)
8.2	Eat 3 servings of legumes per week (1 serving for an adult diet: 70 g raw/125 g cooked)
8.2	Choose plant-based alternatives (*e.g.* legumes, nuts) as protein sources instead of meat
7.8	Limit the consumption of processed meat (both red and white meat) or even avoid it
7.8	Eat 5 servings of vegetables and fruits per day (1 serving for an adult diet: 125 g)
7.7	Limit the consumption of all meat or even avoid it
7.5	Choose a variety of vegetables and fruits
7.5	Eat 3–6 servings of grain-based foods per day (1 serving for an adult diet: *e.g.* 40–60 g bread/60–80 g dried pasta or dried rice)
7.5	Limit the consumption of processed food products high in salt, sugar, and fat (*e.g.* fast food, salty snacks, cookies, bars)
7.5	Drink water instead of sugar-sweetened beverages
7.5	Know your energy (caloric) needs and eat accordingly (do not over/undereat)
7.3	Eat a variety of foods
6.8	Eat primarily whole grains
6.8	Drink 1.5–2 L water (for an adult diet) per day
6.8	Limit alcoholic beverage consumption to 2 glasses per day for men and 1 glass per day for women
6.7	Choose a variety of legumes
6.7	Choose a variety of grain-based foods (*e.g.* flour types, pasta, rice)
6.7	Choose vegetable oils (*e.g.* olive oil, rapeseed oil)
6.4	Choose tap water instead of bottled water
6.4	Do not drink alcohol if under 18 years of age
6.3	Choose organically produced vegetables and fruits
6.3	Eat a small handful of nuts and seeds (2–3 times a week; 1 serving for an adult diet is 25 g)
6.3	Choose eggs instead of meat as an alternative source of protein
6.3	Eat (2–3 servings of fish and seafood per week (1 serving for an adult diet: 125–150 g)
6.3	Choose a variety of nuts and seeds
6.2	Choose organically produced legumes
6.2	Limit the consumption of added sugar (*e.g.* from sweets) (max. 25 g per day for an adult diet)
6.2	Drink unsweetened beverages (e.g. tea) instead of sugar-sweetened beverages
6.2	Ensure an adequate vitamin D intake through sun exposure or supplementation (20 µg/d for an adult)
6.2	Choose poultry instead of red/processed meat
6.0	Choose seasonal vegetables and fruits
6.0	Choose organically produced grains
6.0	Choose organically produced fish
6.0	Eat 2–4 servings of eggs per week (1 serving for an adult diet: 1 egg)
6.0	Choose organically produced oils
6.0	Choose organically produced beverages (*e.g.* tea, coffee, juice)

‘*Choose tap water instead of bottled water*’ was the HIB with the highest feasibility and plasticity but not with the highest technical potential in middle-lower-class families (Figure 4a); ‘*Eat 3–6 servings of grain-based foods per day*’, ‘*Choose a variety of grain-based foods*’, ‘*Choose vegetable oils*’, and ‘*Eat 2–4 servings of eggs per week*’ were the HIBs with the highest feasibility and plasticity but not with the highest technical potential in upper-class families (Figure 4b); and ‘*Choose tap water instead of bottled water*’ and ‘*Choose a variety of grain-based foods*’ were the HIBs with the highest feasibility and plasticity but not with the highest technical potential in the school restaurant setting (Figure 4c). In addition, ‘*Eat 0–3 servings of meat per week*’ (with high technical potential) exhibited high feasibility and plasticity in all settings. However, none of these behaviours are already supported by stakeholders, *i.e.* have a rating above 5 (only ‘Very likely’) (Figures 4a–c).

### LL coordinator interviews

The LL coordinator emphasised that the nutritional behaviour of children at this age is highly dependent on their environment. While meals are largely determined by the school restaurant in school settings, the parents – or legal guardians – are mainly responsible for the children’s choice of food in other settings. Several verbatims have been reported: ‘*When they are in the school canteen, they can’t choose what they eat.* […] *When they are 6 or 10 years old, the food is usually already prepared. The adults fill their plates, and the children have to eat what is put in front of them. However, when they are in high school, they can choose a bit more (e.g. through self-services), depending on what is prepared for the day*’. If children are allowed to decide for themselves what to eat, for example, they may tend to consume fast food: ‘*The beginning of autonomy in terms of food starts in adolescence when they start to have pocket money.* […] *We can assume that they go straight to McDonalds and other fast food restaurants to be with their friends* […] *so fast food and junk food in general is associated with sharing meals with their school friends.* […] *In France, it was published a few years ago that 90% of food advertising to children is for UPFs, junk food. So, the marketing pressure is very targeted at children, which is a big problem in France.* […] *When children are used to these foods, they become customers for life*’.

The LL coordinators are in face-to-face contact with the schools and their target group. They therefore have a good understanding and insight into their target group. For example, they recognised that there is a wide range of heterogeneity within their target group, *e.g* in terms of the age and socio-economic status of the families. Thus, for the LL coordinators, it clearly appeared that families with a low socio-economic status use the school canteen to provide their children with affordable meals that include, *e.g.* meat and fruits, both of which are expensive in France: ‘[…] *we* [LL coordinator] *went to a school with a low socioeconomic status as far as the parents were concerned, and sometimes they sent their children to school without breakfast. So, for the parents, the canteen is a way to provide their children with meat (because it’s expensive) and to provide them with a lot of calories.’* According to the LL coordinator, it would be also difficult to reduce the meat consumption of French children, as it is a tradition in France to include meat in meals: ‘*Meat is associated with strength, especially among boys*’.

Stakeholders who play a decisive role in their target groups are, as far as the school restaurant setting is concerned, school canteen managers, school policy-makers and peers, whereas at home, it is the parents or legal guardians whose acceptance is required for behavioural change in their children. In particular, the LL coordinators saw the potential to work with school canteen managers to improve the food quality in schools: ‘*I think another lever is the quality of the food in the school canteens. So, I think there is a lot to do to improve the food quality in the canteens, because children eat five meals a week in school canteens. Moreover, the children influence their parents. So, if children eat well in the canteen, eat good products, they can influence their parents at home*’.

### Workshop and final selection: the five selected HIBs

Based on the combination of the above-mentioned results (*i.e.* the selection of the top 40 HIBs with high technical potential ≥6, combination of plasticity/feasibility in different settings) and field experiences of the French LL coordinators for the specific children population, the five HIBs selected for French children aged 6–15 years are:

- HIB1: Limit consumption of processed food products high in salt, sugar and fat (FS11 behaviour, Figure 3, low plasticity/feasibility, Figures 4a–c)
- HIB2: Eat 0–3 servings of meat per week (M1 behaviour, Figure 3, high plasticity/feasibility, Figures 4a–c)
- HIB3: Eat 3 servings of legumes per week (L1 behaviour, Figure 3; low to medium plasticity/feasibility, Figures 4a–c)
- HIB4: Choose primarily whole grains (G3 behaviour, Figure 3; low to medium plasticity/feasibility, Figures 4a–c)
- HIB5: Choose to drink water instead of sugar-sweetened beverages (B3 behaviour, Figure 3; medium to high plasticity/feasibility, Figures 4a–c)

HIB 1, 3 and 4 have low to medium plasticity/feasibility, and will be therefore more difficult to implement in our LL real life conditions. HIB 2 and 5 have medium to high plasticity/feasibility, and will be therefore easier to implement in real life LL conditions.

After the discussion in the workshop, the French LL coordinators chose to increment the first three HIBs as regards the French dietary guidelines, the specific condition of the French LL targeted at children aged 6–15 years, the LL coordinator field and empirical experiences among children, their previous research works, and the local upstream support of some HIBs by relevant regional LL stakeholders:

- HIB 1: Limit consumption of UPFs, notably those with added salt, sugar, fat, aroma and/or any other cosmetic additives;
- HIB 2: Reduce all animal-based foods, notably eating 0–3 servings of meat per week;
- HIB 3: Eat a variety of plant-based foods, notably eating 3 servings of legumes per week.

Therefore, among the five pre-selected HIBs proposed by Justus-Liebig University researchers (see SM 1), the French LL coordinators kept four and abandoned the fourth one, *i.e.* ‘*Eat 5 servings of vegetables and fruits per day*’ that was replaced by ‘*Choose primarily whole to refined grains*’, a recommendation more in line with French children reality consuming practically, and almost exclusively, refined cereals at breakfast, snacking time and lunch, *e.g.* white bread.

**Figure 5.**
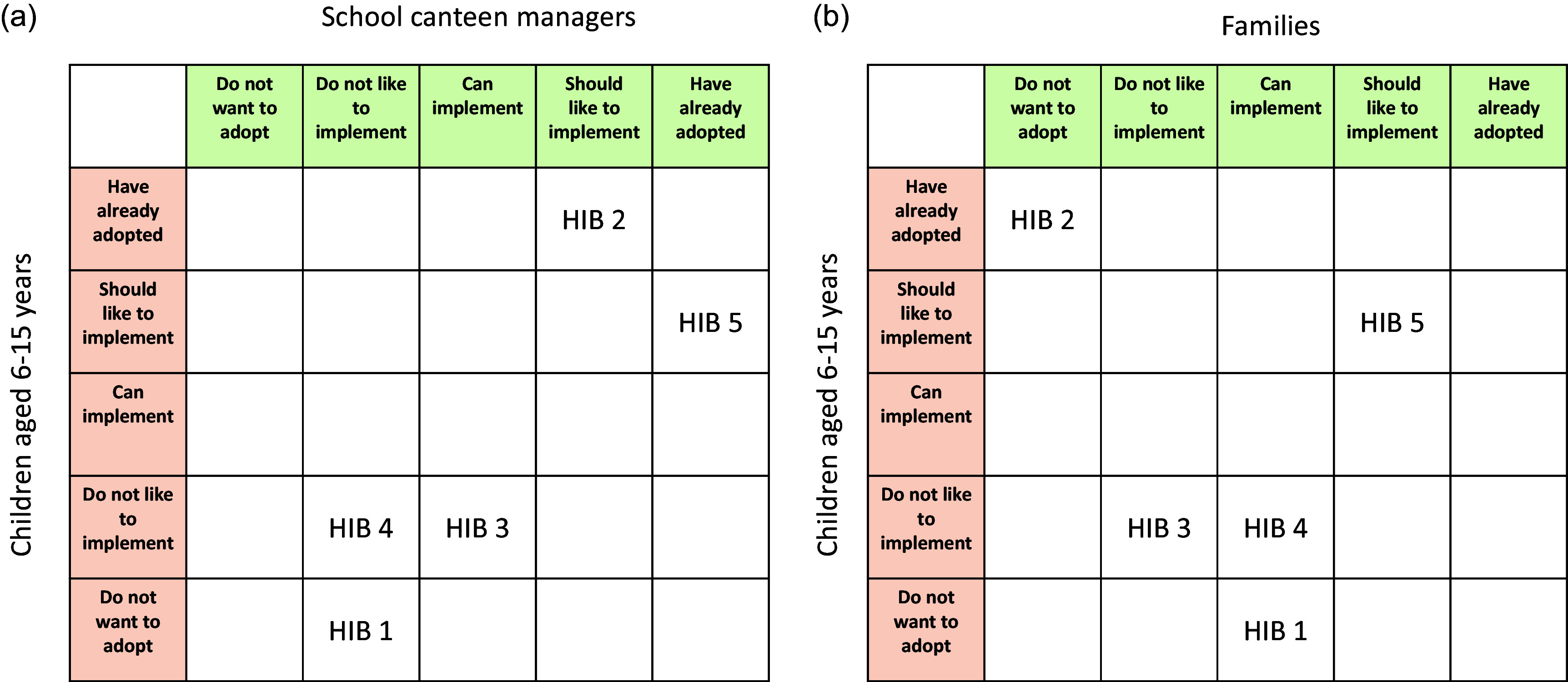
Capacity of LL actors (school canteen managers and families) and plasticity of schoolchildren for engagement in the five HIBs based on a survey completed by seventeen families, interviews with LL stakeholders, field observations, photo language workshops and focus groups in five classes of our LL: **a)** School canteen managers and children, and **b)** families (at home) and children. Abbreviations: HIB 1: Limit consumption of UPFs, notably those with added salt, sugar, fat, aroma and/or any other cosmetic additives; HIB 2: Reduce all animal-based foods, notably eating 0–3 servings of meat per week; HIB 3: Eat a variety of plant-based foods, notably eating 3 servings of legumes per week; HIB 4: Choose primarily whole grains; HIB 5: Choose to drink water instead of sugar-sweetened beverages. LL, living laboratory.

Finally, based on our French LL field research actions, the least feasible HIBs by families are HIBs 2 and 3, and HIBs 1 and 4 for school canteen managers (Figure 5a and b). For children, HIBs 1, 3 and 4 appear difficult to adopt and implement (low plasticity; Figure 5a and b

## Discussion

### Main findings regarding adequacy with the 3V score

The 40 HIBs with the greatest technical potential reveal significant tendencies and can be categorised as follows: reducing the consumption all types of meats, processed foods high in salt, sugar, and fat (including sugar-sweetened beverages), and alcoholic beverages; increasing the consumption all plant-based foods (whole-grain cereals, legumes, nuts, fruits and vegetables) and water while diversifying food intake; prioritising white meat and fish; and choosing organic and seasonal foods. Notably, there is an absence of eating behaviours related to dairy products when only the 40 HIBs with the highest rated technical potential are used.

These tendencies are in accordance with our previous work related to the development of the generic 3V score for global health (Vrai/Veritable, Végétal/Vegetal, Varié/Varied foods), which includes three fundamental dimensions that are used to characterise the diet-global health relationship, and that encompass approximately 76% of the top 40 behaviours, that is, increasing plant-based food consumption and food diversity and reducing meat and UPF consumption.^(36–38)^ These findings show that these generic dimensions have high technical potential, and indicate three strong trends to favourably impact global health, notably from very early childhood.

Our final five HIBs are among those with the highest technical potential (≥6) but not necessarily those with the highest plasticity and feasibility. However, they are all related to the 3V score to protect global health, *i.e.* reducing UPF (HIBs 1 and 5) and animal-based food (HB 2) consumption, and replacing these foods with legumes (HIB 3), whole-grain cereals (HIB 4), and nuts. Therefore, implementing the 3V score in our LL should strongly and positively impact children’s health and the environment in the middle- and long-term. Obviously, some of them may be slightly more challenging to change in terms of feasibility and plasticity within the context of the French LL (see discussion below), because they are targeted at children aged 6–15 years, and considering stakeholders supporting the LL. However, our objective is precisely to improve and implement HIBs with high technical potential, and which, initially, are not necessarily highly feasible and plastic.

### The feasibility and plasticity of the five HIBs

- HIB 1: ‘Limit consumption of UPFs, notably those with added salt, sugar, fat, aroma and/or any other cosmetic additives’

UPFs are highly affordable, available and accessible to children, and they are supported by ‘aggressive’ food advertising (in France, the majority of advertisements for children’s foods were for UPFs),^(39)^ family choices at home (*e.g.* breakfast and snack times), school catering, and/or the establishment of fast-food restaurants and supermarkets around middle and high schools, which is the time of life during which children begin to have some pocket money. They are also designed to be highly palatable, if not addictive;^(40,41)^ for many of them, this is achieved by the addition of fat, salt, sugar and/or cosmetic agents (*i.e.* texturising and colouring agents, taste modifiers, and other nonadditive compounds such as aromas and/or glucose syrup).^(5)^ The resulting standardised taste for each food category increases the risk of children having difficulty appreciating the subtler and demanding tastes of real, non-UPF foods. This artificial attractiveness results in high consumption of UPF products. In France, we recently reported that the number of calories from UPFs is 46% of the total number of daily calories in children under the age of 18 years.^(42)^ Such a high level of UPF consumption may have a considerable impact on both health and the environment in the long term.^(6)^

Due to these environmental factors at the meso-level, it may be difficult to challenge the plasticity of this HIB among children, *i.e.* to reduce UPF consumption. Therefore, a good understanding of the factors leading children to UPFs is mandatory to identify levers of action. Among them, food education at school and information provided to children’s parents may be potential solutions to investigate,^(43)^ especially within the context of the ‘whole-school approach’.^(44)^

With respect to feasibility as assessed by the stakeholders, we observed in our LL that some stakeholders, such as parents, and health and educational professionals, researchers and civil society, are motivated to change this eating behaviour, but this appears more difficult for agri-food chain actors and politicians. Notably, in food retail, UPFs are generally highly affordable and accessible^(18)^ and thus are highly profitable for stores. Notably, UPFs account for approximately 70% of all marketed and packaged industrial foods in French food super- and hyper-markets^(45)^.

- HIB 2: ‘Reducing all animal-based foods, notably eating 0–3 servings of meat per week’

In France, the percentage of calories from animal-based foods consumed daily is approximately 39% for children under the age of 18 years,^(42)^ which is high compared with that in low-income countries and the average global consumption.^(46)^ In addition, culinary traditions in the LL area (Figure 1), especially in rural areas, are based mainly on animal-based foods, such as cheese, cow/sheep meat, and cold cuts. The recent French EGALIM Law now imposes one vegetarian menu per week in the school canteen.^(47)^ Recent data in Europe (1990–2018) clearly show a decrease in unprocessed red meat consumption in children,^(46)^ with an increased rate of consumption of plant-based diets in high-income countries,^(48)^ indicating the potentially good plasticity of this HIB. However, in our region, we observed, based on LL school canteen observations and stakeholder interviews, that meat at school often appears to be the only meal with meat for disadvantaged families.

With respect to stakeholders, several barriers and levers are linked to feasibility. For example, we observed that the main barrier is the difficulty faced by school cooks in proposing tasty vegetarian meals or plant-based alternatives, as also reported by the School Meals Coalition for France,^(49)^ especially meals that include legumes, because they have been trained to cook meals with meat as a core ingredient. In addition, in more rural areas of our region, there is strong resistance to increasing plant-based foods and reducing meat consumption.

More generally, the core idea is to reduce animal-based food consumption while simultaneously moving toward animal-based foods of higher quality, not exceeding 3 servings/day,^(37)^ and more specifically staying in the range of 0–3 servings/week of red meat. Some stakeholders in our region are prone to accompany this evolution through a regional platform for the agro-food actors toward plant-based foods. In addition, a possibility may be also to develop and relaunch local short circuits based on high-quality local and traditional cow breeds for extensive breeding and resistance to climate change. This would entail the redistribution of cereal crops toward more human food in our region while reducing animal feeding, especially for animals kept under intensive conditions.

- HIB 3: ‘Eat a variety of plant-based foods, notably eating 3 servings of legumes per week’

Food variety may be indirectly evaluated through the prevalence of nutrient deficiencies. In France, a survey conducted in 2014 revealed that the nutritional needs of children aged 3–10 years were quite good, except for insufficient consumption of EPA + DHA and excessive consumption of free sugar (above the threshold of a maximum of 10% of daily calories),^(28,50)^ reflecting acceptable food diversity. In addition, during the same year, slightly fewer than one in five children aged 3 to 17 years (18.5%) consumed at least one food supplement (in the broad sense) over the last 12 months.^(33)^ Therefore, increasing the variety of plant food, preferably minimally processed food (HIB n°1), should meet the adequate nutritional needs of all children.

However, among plant-based foods, fruits and vegetables are the food groups most often rejected by children,^(51)^ and we observed in our LL that children prefer fruit juices or fruit purées to solid raw fruits for snacks, and that they do not prefer legumes (*e.g.* lentils). Thus, in France in 2014, the mean daily legume consumption in children (0–17 years) was 3–4 g,^(28)^ suggesting difficulty in challenging this HIB regarding legume diversity, *i.e.* a low plasticity.

With respect to stakeholders, in our region, a variety of protected design of origin (PDO)-labelled lentils are recognised internationally (*i.e.* The Green Lentil du Puy AOP, Le Puy-en-Velay, France), and the possibility of incorporating lentil flour into traditional bread recipes reflects existing potential.^(52)^ In schools, we also observed that cooks are not used to preparing tasty legume-based foods or meals. However, the increase in legume consumption may accompany HIB 2 of reducing red meat consumption and, more generally, meat-based protein consumption. One possibility may be to design new legume-based recipes for school canteens.

- HIB 4: ‘Choose primarily whole grains (instead of refined ones)’

In France, in 2014, most cereal-based foods consumed by children under the age of 18 were refined (*i.e.* approximately 98% of the daily cereal-based food quantity).^(28)^ Refined cereals are consumed mainly for breakfast, in school canteens in the form of (industrial) white breads and desserts, in families at home in the form of white pasta and rice, and at snack times in the form of industrial biscuits/cookies, pastries and cakes. The margin associated with improving this HIB is therefore significant, and very meaningful with respect to the numerous reported beneficial human health effects of whole-grain cereals.^(53)^ In addition, whole-grain cereals are generally as much appreciated as refined cereals by children.^(54,55)^ However, the potential plasticity of this HIB in our LL still appears to be low to medium.

Concerning feasibility by stakeholders, a previous review reported several actions to facilitate whole-grain cereal consumption, *e.g. ‘to increase the availability and the variety of foods containing whole-grain cereals, to improve their sensory appeal, to reduce their purchase cost, to use a familiarization period to introduce them to consumers (with a gradual increase in consumed amounts and repeated exposure), and to improve communication and labeling to enhance consumers’ ability to identify products with whole-grain cereals*’.^(56)^

- HIB 5: ‘Choose to drink water instead of sugar-sweetened beverages’

In France in 2014, children under the age of 18 years were accustomed to consuming approximately 120 g of industrial sweetened beverages daily (fruit juices excluded), *i.e.* almost one glass/day, which approximately corresponded to 11% of all beverages consumed daily.^(28)^ Vegetable and fruit juices constitute approximately 8–9% of all beverages consumed, and water accounts for approximately 53% of all beverages consumed.^(28)^

Generally, sodas and sweet drinks are sold with ‘cute packaging’, which is highly attractive to target children, especially at snack times. The plasticity of HIB 5 is also very challenging, notably because water is generally considered unappealing by children.^(57)^ Concerning feasibility by stakeholders, one of the main challenges appears to be the decrease in their affordability, availability and accessibility,^(58)^ especially in beverage vending machines^(59)^ and supermarkets.^(60)^ Notably, the presence of soda in big food retail stores is important for attracting customers who will buy other food in the same store, and stores that forgo selling soda risk having their customers travel to another store.

### Strengths and limitations

The main strength of this study is that the HIB selection combines both top-down (*e.g.* reducing the number of eating behaviours, technical potential) and bottom-up (empirical data collected through expert interviews and workshops, notably concerning feasibility and plasticity) approaches, which allows us to more closely align HIB with our regional LL reality. However, the study has also some limitations: thus, the five HIBs probably do not cover all aspects of global health, notably with respect to children. However, being included in the framework of the 3V score, their impact remains high for global health, including human health, and environmental and socio-economic impacts.^(37)^ Another limit is that online surveys and interviews (see SM 3–6) have been addressed by only a few PLAN’EAT experts and LL coordinators, leaving room for potential subjectivity. Nevertheless, this limit is eased by the fact that the French LL coordinators knew the reality of their LL, and chosen experts were complementary in their expertise.

## Conclusions and perspectives

The five selected HIB might be not generalisable to all European children populations because depending on the specific conditions of our LL as regards culinary tradition (*e.g.* a high level of animal product consumption in our region, especially in rural areas), or the commitment of the LL stakeholder for feasibility of HIB changes (bottom-up approach). However, the reduction of UPF, animal products, refined cereals and sweetened beverages, and the increase in legume consumption is valid for the entire French children population as we reported,^(42)^ and based on the last representative French INCA3 survey.^(28)^ These HIB changes are promoted in the last French PNNS4 (2019–2023),^(11)^ and also in the next upcoming PNNS5 (2026–2030);^(61)^ but this remains a top-down approach with all its limits for implementation on the field. Therefore, for future practice and policy, this would involve an approach combining PNNS recommendations, policy measures (*e.g.* UPF taxes, ban on UPF advertising targeted at children, in complementarity with field actors for their effective implementation (*e.g.* train school cooks to better cook legumes, reducing UPF by food services for school canteens…)).

Thus, these five HIBs constitute a solid basis for further investigations into our LL on the basis of participatory studies, notably based on their observed potential plasticity in children. The next step will be to implement these actions and improve HIB feasibility under real-life conditions. This can be achieved only through transversal actions with all non-researcher stakeholders, despite often having opposite interests. Thus, the LL methodology appears to be a relevant holistic tool for addressing these complex HIB-related issues.

Finally, considering a more global perspective, beyond the specific context of this study and other European countries, the five HIBs are also in agreement with the Planetary Health Diet Index adapted for children and adolescents, that promotes whole-cereals, legumes, moderated animal-based foods’ and reduced added sugars consumption as, *e.g.* in sweetened beverages.^(62)^ However, it would be also necessary to lay more emphasis on UPFs for such children recommendations, notably because they contain potentially unhealthy non-nutrient industrial agents, *i.e.* markers of ultra-processing; and, in this way, our selected five HIBs may constitute a global generic framework for studying children eating behaviours.

## Supporting information

10.1017/jns.2026.10105.sm001 Fardet et al. supplementary material 1 Fardet et al. supplementary material

10.1017/jns.2026.10105.sm002 Fardet et al. supplementary material 2 Fardet et al. supplementary material

10.1017/jns.2026.10105.sm003 Fardet et al. supplementary material 3 Fardet et al. supplementary material

10.1017/jns.2026.10105.sm004 Fardet et al. supplementary material 4 Fardet et al. supplementary material

10.1017/jns.2026.10105.sm005 Fardet et al. supplementary material 5 Fardet et al. supplementary material

10.1017/jns.2026.10105.sm006 Fardet et al. supplementary material 6 Fardet et al. supplementary material

10.1017/jns.2026.10105.sm007 Fardet et al. supplementary material 7 Fardet et al. supplementary material

10.1017/jns.2026.10105.sm008 Fardet et al. supplementary material 8 Fardet et al. supplementary material

## Supplementary material

The supplementary material for this article can be found at https://doi.org/10.1017/jns.2026.10105.
