# Supplementary material for: Identifying healthy and sustainable high-impact eating behaviour in French children aged 6–15 years: a combined multidisciplinary and living lab participatory approach
Source: J Nutr Sci. 2026 May 26;15:e38. doi: 10.1017/jns.2026.10105 (PMC13227139; doi:10.1017/jns.2026.10105)
Supplement: Fardet et al. supplementary material 1 — Fardet et al. supplementary material [file S2048679026101050sup001.pdf]

Supplementary material n°1

Country	Target groups	Dietary behaviour	Health relation	Environment relation	Social relation	Source (Sharepoint doc. SPG1)
Germany DE (6-18, obese)	General population DE1	Enjoy food diversity	health-promoting	sustainable		10 guidelines of the German Nutrition Society (DGE): https://www.dge.de/ernaehrungspraxis/vollwertige-ernaehrung/10-regeln-der-dge/en/
		Choose mainly plant-based foods	health-promoting	sustainable		
		Eat 5 portions of vegetable and fruits per day	lower the risk of cardiovascular and other diseases	Regional seasonal products have a better environmental impact, as resources for storage and long transport routes can be saved.	Regional seasonal products are often cheaper	
		Favour whole grain foods	reduce the risk of type 2 diabetes mellitus, dyslipidemia, colon cancer and cardiovascular diseases			
		Consume milk and dairy products such as yoghurt and cheese daily	supports bone health and is also associated with a reduced risk of colon cancer.			
		Consume fish once to twice a week: Choose fish from sustainable fisheries or sustainably operated aquacultures	importance for the health of the cardiovascular system and reduces			

			the risk of stroke.			
		You should not consume more than 300 to 600 g meat per week	People who eat a lot of red meat and sausage have a higher risk of colorectal cancer. A small amount of meat can help provide vital nutrients (available iron as well as selenium and zinc).	In the production of animal foods, the usage of resources and the emission of harmful greenhouse gases is higher than in the production of plant foods.		
		Prefer vegetable oils	This can reduce the risk of cardiovascular disease.			
		Avoid hidden fats (processed foods)				
		Limit the consumption of salt (6g/d), Choose iodised and fluoridated salt.	Too much salt in your food can increase your blood pressure and thus also the risk of cardiovascular disease.			
		Avoid sugar-sweetened foods and beverages	Sugar increases the risk of caries. Increase the risk of obesity and			

			type 2 diabetes mellitus			
		Flavour with herbs and spices				
		Drink about 1.5 litres per day: Water or other calorie-free beverages, such as unsweetened tea		If you use tap water instead of buying bottled water, you save on packaging and transportation.	If you use tap water instead of buying bottled water, you save money.	
		Cook food as short as possible	A careful preparation will preserve the natural taste and conserve the nutrients. Burnt parts contain harmful substances.	Protect the climate by saving energy when cooking and baking (use residual heat).	Save money by saving energy when cooking and baking (use residual heat).	
		Eat mindfully	Promote pleasure, relax and help regulate body weight.	Check your supplies. Use a shopping list to buy only what you need. Reuse leftovers or freeze them.		

		Watch you weight: eat a wholesome diet, stay active		Walking or biking is the more environmentally friendly alternative to driving.		
		Buy organic quality food.		Organic farming takes into account not only the aspects of environmental protection, but also animal welfare.		
	Pregnancy and lactation DE4	Eat a balanced diet	has a positive effect on the optimal development and health of the child		has a positive effect on the well-being of the mother	DGE Population groups: https://www.dge.de/ernaehrungspraxis/bevoelkerungsgruppen/
		Make the right food choices	Foodborne infections, such as toxoplasmosis or listeria, can pass to the unborn child and lead to severe illness and even death.			
		Breast milk in the first 4 to 6 months	According to requirements of the child		Strengthens emotional bond of	

					mother and child.	
	Children DE2	optimal supply of all nutrients (wholesome diet)	Physical and mental development, concentration and performance, as well as strengthening the immune system.		"DGE Quality Standard for Catering in Day Care Facilities for Children."	
	Employees DE5	A diet adapted to the daily work routine	positive effects on the ability to perform and concentrate and promotes long-term health.		"DGE quality standard for company catering"	
	Elderly DE6	Meeting nutrient needs, adapted to age-related changes	Changes in body composition, weight, energy and nutrient requirements, thirst sensation, altered sensory perceptions, masticatory performance, and digestion and gastrointestinal tract.		"DGE Quality Standard for Catering in Inpatient Senior Facilities" and the "DGE Quality Standard for Meals on Wheels".	
	Athletes DE9	Apply adapted nutrition strategies	to maximize physical and			

			mental performance			
		individual supply of minerals and vitamins to meet individual needs	essential for health and performance			
		Supplements (NEM) are not a substitute for a balanced diet.			There is no government audit of the safety of dietary supplements (NEMs)	
France FR (6-18)	General population FR1	at least 5 fruits and vegetables a day				FRANCE'S NATIONAL FOOD PROGRAMME 2019-2023 https://agriculture.gouv.fr/telecharger/108904?token=3f9e45da2753232d4d6553c263ce52d9f07f0497525102410e04e1ef47fb8f59 + French National Nutrition and Health Program 2011-2015 https://sante.gouv.fr/IMG/pdf/PNNS_UK_INDD_V2.pdf
		consume legumes (lentils, beans, chickpeas, etc.) at least twice a week				
		at least 30 minutes of vigorous physical activity per day				
		Don't sit too long, take the time to walk a little every 2 hours				
		at least one wholegrain starchy food per day				
		Fish: Twice a week, including a fatty fish				
		choose rapeseed, walnut and olive oil: added fats – oil, butter and margarine – can be consumed daily in small amounts				
		Dairy products: milk, yoghurt, cheese and cottage cheese: 2 per day for adults				
		Alcohol, maximum 2 glasses per day and not every day				
		Limit sugary drinks, fatty, sugary, salty and ultra-processed foods			promotion of Nutri-Score, improve	

					the nutritional quality of all processed foods	
		Limit salty products and salt				
		Limit delicatessen to 150 g per week				
		Favor poultry and limit other meats (pork, beef, veal, mutton, lamb, offal) to 500 g per week				
		Increase the consumption of organic products				
	Pregnancy and lactation FR4	Promote breastfeeding			respecting the woman's decision, promoting an environment conducive to breastfeeding by indicating places that are committed to facilitating breastfeeding for women	
		folic acid supplementation in women wishing to become pregnant				
	Elderly FR6	2 to 3 dairy products are recommended for people over 75				
		Consume at least once a day Meat/Fish/Eggs				

		1.5 L of drinks per day and avoid excessive consumption.				
	children and teenagers FR3	Starchy foods: at each meal and according to appetite				
		Dairy products: 3 or 4 per day				
		Meat, egg, fish: 1 to 2 times a day, alternating				
		limit sweet products				
		Water: unlimited during and between meals				
		combat sedentary behaviour among children and adolescents				
	high-risk groups FR7	increase calcium intake in young women, adolescents and elderly people				
		reduce iron deficiency in women living in poverty				
		improve the folate status of women of childbearing age				
		Supporting the development of food education in prison setting				
Ireland IE (18-30)	General population IE1	Eat more vegetables, salad and fruit - Up to seven servings a day	Enjoy a variety of coloured fruit and vegetables to benefit from the different minerals and vitamins each contains			https://www.hse.ie/eng/about/who/healthwellbeing/our-priority-programmes/heal/healthy-eating-guidelines/
		Limit intake of high fat, sugar, salt (HFSS) food and drinks				
		Size matters: Use the food pyramid as a guide for serving sizes				
		Increase your physical activity levels				

		Small changes can make a big difference. Start TODAY!				
		Beans and eggs are good sources of protein and are low in fat. They are a good choice for meatfree days.				
		Keeping active (Getting started; Walking/running/cycling; Keeping kids active; Activity for older people; Protect your skin; Be Summer Ready)				https://www.gov.ie/en/campaigns/healthy-ireland/
		Eating well (Tips for healthy eating; Tips for older people; Quick meals; Snacks and treat foods; Takeaways; Cooking with children)				
		Minding your mood (Getting enough sleep; Switching off; Keep learning and being creative; Spending time in nature; Giving to others; Returning to the workplace; Anxiety around COVID-19; Keeping in contact)				
	Children IE2	Milk is a key food, with a daily intake of 550ml of cow's milk, or equivalent amounts of yoghurt or cheese, is recommended.				https://www.fsai.ie/news_centre/press_releases/healthy_eating_1-5yearolds_22062020.html
		Water and milk are the only drinks recommended for this age group. Sugar-containing and acidic drinks should be limited and, if consumed at all, should be kept to mealtimes.				
		Parents and guardians are warned against using some beverages such as almond 'milk', coconut 'milk' and rice 'milk', as milk substitutes as these are nutritionally inadequate. If a plant-based beverage is required to				

		replace cow's milk, a soya 'milk', can be used, provided it is fortified with nutrients, particularly calcium.				
		A portion of vegetables should always be included at the main meal, together with the number of small portions of salad, vegetables or fruit that match the age of the child, for example two small portions for a two year old, four small portions for a four year old (the portion size given should fit into the child's hand so that smaller children are given less and bigger children more).	There is very little room for such foods in a 1 to 5 year-old's diet, so such foods either overwhelm the child's capacity for nutritious foods or provide additional calories that lead to the development of overweight or obesity.			
		Lean red meat (about 30g) is recommended three days a week for iron and other essential minerals in addition to protein. On other days, red meat can be replaced with poultry, fish, eggs, beans or lentils which also provide iron, as well protein and minerals. Smooth nut butters also provide protein.				
		A combination of both white and wholemeal breads, cereals, potatoes, pastas and rice will provide adequate fibre and are important sources of calories.				
		Foods high in fat, high in sugar or salt such as confectionery, cakes, crisps, biscuits and sugar-coated				

		breakfast cereals are not recommended.				
		Fats, spreads and oils should be used minimally.				
		During the extended winter months – from Hallowe'en to St Patrick's day, all children aged 1 to 5 years need to be given a low-dose (5 µg) vitamin D-only supplement to make up for lack of skin synthesis of this vitamin from sunlight.				
		Young children aged 1 to 3 years, who are naturally small (25th percentile or less on growth charts) need extra iron, which can be taken as an iron-fortified full-fat milk, or a low-dose iron supplement.				https://www.fsai.ie/news_centre/press_releases/dietary_guidelines_over65s_19042021.html
		Encouraging acceptance of the wide range of flavours and textures naturally found in the food people need for healthy eating throughout life is important at this young life stage. Fostering a tolerance to 'try' an expanding variety of vegetables, salads, fruits, meat, fish and wholemeal cereals helps children develop a taste for nutritious foods. To assist this, small amounts of sugar can be used e.g. in stewed fruit, milk puddings, in jam on wholemeal bread or as a small portion of ice cream on fruit etc.				

		If a key food group is excluded from a child's diet because a food allergy is suspected without proper indication or appropriate professional advice being sought, the growth and development of a child can be compromised.				
	Older adults IE6	Older adults who are obese with weight-related health problems should receive individual intervention to ensure weight reduction undertaken is beneficial and minimises loss of muscle tissue (slow weight loss with physical activity). Lower risk older adults who are overweight are advised to avoid weight-loss diets in order to prevent loss of muscle mass.				
		Older adults at risk of 'low intake' dehydration need adequate amount of drinks. Women need 1.6ltrs and males 2ltrs per day (unless a clinical condition to require fluid restriction).				
		Strong tea should only be consumed between meals and not during meals, as it interferes with absorption of iron and zinc.	to prevent developmen t of frailty, muscle loss (sarcopenia) and undernutriti on.			
		Sense of taste diminishes with age and can lead to increased salt intake; therefore, consumption of salty foods should be avoided and alternatives such as herbs and spices can be used to increase flavour.				

		High quality proteins to stimulate muscle protein: Healthy older adults should eat a more protein-dense diet – foods such as meat poultry, fish, dairy and eggs.				
		Adequate calorie intake				
		Diets should contain high fibre carbohydrates, but low in free sugars. The average intake of carbohydrates are at the lower end of recommended consumption range whilst one third of older people exceed recommended free sugar intake.				
		A daily 15 µg vitamin D supplement is now recommended by the Department of Health for all older adults in Ireland. This report provides specific details on the range of dietary intake recommended for vitamin D in older adults, which vary according to ability to obtain some of this vitamin from sunlight exposure.				
		Fortified foods are a good source of B vitamins (B12, folate, B6 and riboflavin) and vitamin D; whilst unsweetened orange juice, salads, fruit and vegetables are reliable daily food sources of vitamin C.				
Sweden SE (<6 & pregnant)	General population SE1	minimum of 500 g fruit and greens daily		For greens and fruits, coarse, hardy vegetables are promoted		https://www.livsmedelsverket.se/globalassets/publikationsdatabas/andra-sprak/kostraden/kostrad-eng.pdf

				over salad due to their longer shelf life and that they can be grown outdoors, and eating according to season.		
		70-90 g wholegrains daily				
		fish 2-3 times a week				
		less than 500 g red meat – and as little charcuterie as possible				
		2-5 dl of low fat dairy		negative effects caused by methane from ruminants are negative while grazing helps to keep the land open and contribute to biodiversit y in Sweden.		
		less than 6g salt – 2,4 g Na				
		a couple of tablespoons of nuts each day				
		more vegetables and fruit				

		more seafood	positive health aspects	environmental issues related to fishery (over fishing etc.)		
		switch to wholegrain: It is recommended for women to consume 70 g per day and 90 g per day for men				
		switch to healthy fats				
		switch to low fat dairy products				
		eat less red meat and processed meat				
		less salt				
		less sugar				
		maintain energy balance				
		promote a minimum of 30 min of physical activity per day: choosing the stairs, fun exercise (with others), active micropauses and get the heart beating				
		choose foods with the keyhole label and sustainability labels				
		eat less meat and meat products and more plant-foods including whole grains, vegetables and fruit, as well as healthy oils and some fish	to decrease the risk of common chronic diseases in Sweden, especially cardiovascular disease, overweight/ obesity, type 2 diabetes and certain	guidance on how to eat healthily and in an environmentally friendly manner.		

			types of cancer.			
		reduce food waste				
		Regular meals without snacking are recommended (including breakfast, lunch and dinner)				
		alcohol should be restricted due to high calorie content (or avoided for pregnant women)				
		they promote compiling to the general dietary guidelines to loose excessive weight				
	Pregnant and breastfeeding women SE4	focus on nutrient dense foods since the nutrient need increases during pregnancy while the energy need doesn't increase as much				
		supplements if they do not consume enough omega-3 fatty acids, as well as vitamin D and folic acid. Iron levels are monitored during pregnancy and supplements are prescribed when needed.				
		Pregnant women are recommended to avoid different foods and beverages e.g., alcohol, some fish (e.g., Baltic herring and fatty fish from lakes), large quantities of ginger, restrict the caffeine intake to 200 milligrams per day, avoid ginseng, blue cheese (listeria), prepacked salads, sandwiches (listeria) and game (lead)				
	small children (1-2 years) SE2	Eat three nutrient dense meals and 2-3 nutrient dense snacks each day and home-cooked food should include 1 teaspoon extra fat (margarine or vegetable oils				

		with high levels of unsaturated fat).				
		Consume whole grains (though a mix between the refined flours/grains and whole grains are recommended)				
		Consume of fruit and greens daily, however green leafs should be avoided until 12 months				
		All children (1-2 years) in Sweden are recommended vitamin D supplements, and some children above 2 years are recommended to continue supplementation – dark skinned, children not being outside or wear long-sleeves/scarfs, those who not consume fortified products or eat fish.				
		Eat iron supplemented foods (porridge and formulas)				
		Limited salt, caffeine and licorice intake				
		No sweetened drinks to food, minimal amounts of sweets/snacks/drinks, and serve small portions (let the child take twice if they want)				
		Have water quality checked if water comes from own well				
		No intake of green potatoes, honey, unpasteurized milk, uncooked meat				
		Not more than 0.5 tea spoons cinnamon				
	children and adolescent	the food should be nutrient dense and served regularly and accordingly to the plate model				

	s (2-17 years) SE3	sweets and snacks should be restricted				
		Children below 4 are recommended to eat fruits and greens every day and preferably to every meal and increasing the quantity so that they consume 400 g at the age of 4. For children above 10 years of age the recommendation is 500 g per day				
		children are recommended to consume fiber				
		avoid kidney and liver due to cadmium content				
		restrict their intake of cinnamon, flaxseeds, rice products and game meat.				
		Physical activity for 60 minutes each day				
	Elderly SE6	For healthy and active elderly people (65 + years) with normal appetite, there are no adjustments to the general dietary guidelines				
		For healthy elderly with a decreased appetite, the general dietary guidelines apply, though, the plate model is adjusted to smaller portion sizes with a little bit of extra fat, whilst being high in energy and protein and the frequency of meals are increased. The guidelines highlight sufficient protein and vitamin D intake in addition to the general dietary guidelines.				

		Those who have trouble to consume enough calories, it is recommended to add extra fat (oils, margarines or cream). Sugary drinks or products with lots of sugar may be an option if there are severe problems to consume enough calories. There should not be more than 11 hours between the last meal in the evening and breakfast				
		focus on balance and weight training				
Poland PL (<6, 6-18, pregna nt)	General population PL1	Eat more: whole-grain cereal products (e.g. oatmeal, wholemeal bread, w holemeal pasta, groats)				https://ncez.pzh.gov.pl/wp-content/uploads/2021/03/normy_zywienia_2020web.pdf + https://ncez.pzh.gov.pl/abc-zywienia/zasady-zdrowego-zywienia/wiem-ze-dobrze-jem-talerz-zdrowego-zywienia-w-praktyce/
		Eat more: various, colorful vegetables and fruits – more vegetables than fruits				
		Eat more: legume seeds (e.g. beans, peas, chickpeas, lentils, broad beans)				
		Eat more: fish (in particular fatty sea fish)				
		Eat more: low-fat dairy products (in particular fermented)				
		Eat more: nuts and seeds (e.g. walnuts, pumpkin seeds, sunflower seeds)				
		Eat less: salt				
		Eat less: red meat, processed meat (sausages, bacon)				
		Eat less: sugar and sweetened beverages				
		Eat less: processed food products (such as fast food, salty snacks, biscuits, bars) high in salt, sugars and fats				

		Replace: processed cereal products (e.g. white bread, sweet breakfast cereals) with whole-grain cereal products				
		Replace: red meat and processed meat with fish, poultry, eggs, legume seeds and nuts				
		Replace: sweetened beverages with water				
		Replace: animal fats with vegetable oils				
		Replace: full-fat dairy products with low-fat dairy products (milk, yogurt, kefir, buttermilk, and white cheese)				
		Replace: frying, grilling with cooking, including steaming, stewing or baking				
		Be physically active and maintain normal body weight.				
		limit the consumption of products with a high content of saturated fatty acids and cholesterol and replace them with products rich in unsaturated fatty acids	in order to prevent ischemic heart disease. This is especially true for people at high risk, including those with diabetes.			
		STEP 1 - take the first step,				
		STEP 2- implement the recommended level,				
		STEP 3 – achieve further health benefits.				
		Eat vegetables and fruit as often and as much as possible, at least half of what you eat. Remember the	eat a variety of fruit and vegetables - take	buy locally, visit your nearest		

		correct proportions: 3/4 - vegetables and 1/4 - fruit. (The minimum is 400g of vegetables and fruit per day, but the more the better, with a ratio of more vegetables than fruit.)	advantage of the abundance of colours and flavours;<br><br>eat purple, blue and red berries (blueberries, cherries, currants, raspberries, blackberries) - their colour is linked to the presence of anthocyanins, which reduce the risk of cardiovascular disease and cancer, among other things;<br><br>frequently eat cruciferous vegetables (e.g. broccoli, cauliflower, cabbage, kohlrabi) - the glucosinolates they contain reduce the risk of cancer;<br><br>eat	market or bazaar - buying local and seasonal fruit and vegetables is also good for the environment."		

			tomatoes and tomato products frequently - the lycopene they contain reduces the risk of cancer and cardiovascular disease; eat purple, blue and red berries (blueberries, cherries, currants, raspberries, blackberries) - their colour is linked to the presence of anthocyanins, which reduce the risk of cardiovascular disease and cancer, among other things; frequently eat cruciferous vegetables (e.g. broccoli, cauliflower, cabbage, kohlrabi) - the glucosinolates they contain reduce the			

			risk of cancer; eat tomatoes and tomato products frequently - the lycopene they contain reduces the risk of cancer and cardiovascular disease;			
		Limit your meat intake (especially red meat and processed meat products to 0.5 kg/week). Eat fish, pulses and eggs.				
		Prepare a legume-based dish at least twice a week.				
		Nuts, seeds, seeds - a handful a day for good health! Reach for a handful of unsalted nuts, seeds or pips (30 g) every day. Add them to your meal or eat them as a snack.				
		at least 3 portions (90 g) of whole grain cereals (i.e. whole grain milled products) such as groats (e.g. buckwheat), wholemeal bread or wholemeal pasta should be consumed daily.	For the sake of good health			
		It is not recommended to consume more than 350-500 g of red meat and processed meat products per week				
	Children PL3	Let's give your child fish, preferably twice a week				
	Healthy adults HU5	Reduction of salt, sugars and fats as much as possible.				https://www.okostanyer.hu/wp-content/uploads/2021/11/2021_OKOSTANYER_ANGOL_felnott_A4.pdf

Hungary HU (18-30)		Consume not more than 350-500g a week of cooked / steamed / fried (500-700 g raw) red meat (e.g. beef, pork).		Choose a variety of protein sources, you can replace the meat with fish, egg, dairy products, legumes, cereals and seeds. From meats, choose lean variants more often.		
		Eat processed meat products only occasionally, in small amounts.				
		Eat fish at least once a week. Choose from domestic fishes more often (e.g. trout, catfish, bighead carp).				
		Have at least 5 portions of vegetables or fruit a day. Of this, 3-4 portions of vegetables / 1-2 portions of fruit and at least 1 portion of fresh / raw.				
		Eat 3 portions of cereals (e.g. rice, flour products, breads, pastries, biscuits, muesli bars, etc.) every day, of which at least 1 portion should be wholegrain (wholemeal flour, brown rice, oatmeal, wholemeal pasta, bulgur, couscous, millet, buckwheat, wholemeal biscuits, etc.).				

		Eat dry legumes (dry beans, lentils, yellow peas, etc.) regularly.			After opening, store them in airtight containers and in a cool, dry place to preserve their quality.	
		Eat small handfuls of nuts, unsalted almonds, hazelnuts, oilseeds such as pumpkin seeds, sunflower seeds 2-3 times a week.				
		Consumption of local products is preferred				
		seasonality and preference for local food	the nutritional value of products sourced locally will be much higher		promoting the consumption of Hungarian and domestic foods: this will also help to develop the Hungarian economy; only Hungarian products can serve the tastes and habits of the country	

		fighting food waste				
		more plant-based diets, less meat				
		Pay attention to the quantity and quality of food and drink you consume!				
		Choose more seasonal, freshly consumable ingredients and less processed foods.				
		favour the domestic, locally produced ingredients!				
		Drink plenty of fluids				
		eat regularly, have 3-5 meals a day				
		eat a varied, balanced diet				
		A healthy diet is more than the consumed food in itself. Eating in calm conditions with pleasure has countless benefits.				
		Pick those activities, that you like and do them for at least 10 minutes. Increase the time of the exercise step-by-step: the more physical activity you do, the more your health benefits. For adults at least 150 minutes of moderate intensity or 75 minutes of intense exercise is recommended a week.				
	Healthy infants (0-12 months) H U2	Exclusive and responsive (on-demand) breastfeeding is recommended up to 5-6 months of age, regardless of gestational age and birth weight (even after initiation of complementary feeding).				https://www.okostanyer.hu/wp-content/uploads/2018/09/OKOSTANYER-6-17-eveseknek_2old_FINAL_EN.pdf
	children aged 6-17 HU3	Eat at least 4 portions of fruit or vegetables a day! Minimum 1 portion should be fresh or raw. Potatoes can				https://www.okostanyer.hu/wp-content/uploads/2018/09/OKOSTANYER-6-17-eveseknek_2old_FINAL_EN.pdf

		not be calculated into the 4 daily servings.				
		Eat 3 portions of grains a day, of which at least one portion should be whole grain.				
		All main meals should contain complete protein.				
		3 servings of milk or dairy products in an equivalent amount is recommended a day.				
		Drink 8 glasses (8 x 1.5 to 3 dl) of f luids a day, and greater number of which (at least 5 glasses) should be water				
	people over 60 HU6	1. Energy balance				https://merokanal.hu/wp-content/uploads/2019/10/60pluszegeszseg_kiadvany_web.pdf
		2. Fruit, vegetables, every day				
		3. Whole grains, every day				
		4. Animal protein (fish, meat, eggs or milk, dairy products) preferably at every main meal				
		5. Milk, dairy products every day				
		6. 2 litres of fluid daily				
		7. Less salt, added sugar and saturated fat				
		8. Variety, seasonality, preference for local products				
Spain ES (middl e-age, work life to retirem	General population ES1	adoption of a varied and balanced diet pattern				https://www.aesan.gob.es/AECOSAN/docs/documentos/seguridad_alimentaria/evaluacion_riesgos/informes_comite/INFORME_RECOMENDACIONES_DIETETICAS.pdf
		a greater predominance of plant based foods and a lower presence of animal foods	can improve the state of health and well-being	reducing the environm ental impact		

ent, elderly)		physical activity can be integrated into work, in educational centers, sports and recreational activities or when traveling, as well as in daily and domestic tasks, and that it increases the number of steps per day is also a good way to improve the health of all people				
		5 servings of fruit and vegetables per day (3 servings of fruit and at least 2 servings of vegetables)				
		4 servings of legumes per week				
		3 portion of nuts or more servings per week.				
		3-6 servings grain-based foods per day (standard portion: 40-60 g bread, 60-80 g pasta, rice)				
		0-3 servings/week for meat (standard portion: 100-125 g)				
		For processed meat: reduce or even avoid consumption				
		Fish and seafood: 3 servings/week. Standard portion 125-150 g.				
Italy IT (18-30, middle -age)	General population IT1	Directive 1. Keep your weight under control and be always active.				https://www.crea.gov.it/web/alimenti-e-nutrizione/-/linee-guida-per-una-sana-alimentazione-2018
		Directive 2. Eat more fruit and vegetables				
		Directive 3. Eat more whole-grains and legumes				
		Directive 4. Drink abundant water every day				
		Directive 5. Fats, choose the quality and limit the quantity				
		Directive 6. Sugar, sweets, and sugar-sweetened beverages: less is better				

		Directive 7. Salt, less is better but preferred that iodized				
		Directive 8. Alcoholic beverages, less is better				
		Directive 9. Try to vary you diet, how and why	not only for nutritional and healthy reasons but also to avoid consuming possible dangerous substance repeatedly			
		Directive 10. Special recommendations for specific target groups				
		Directive 11. Be careful about the different types of diet and to the use of supplements that do not have scientific basis				
		Directive 12. The food safety is influenced also by the consumer				
		Directive 13. The sustainability of the diet: everyone can give a contribution				
		Fruits and vegetables; Recommendation 5 servings/day				
		Legumes; Recommendation 2-4 servings/week				
		Nuts; Recommendation 1-3 servings/week				
		Red meat; Recommendation 1 serving/week (serving: 100g) and white meat; Recommendation 1-3 servings/week (serving: 100g)				
		Milk and yoghurt; Recommendation 3 servings/day				

		Cheese; Recommendation 3 servings/week				
		Alcohol; Recommendation (for healthy people): < 2 alcoholic units** for an adult male, < 1 alcohol unit for an adult woman; 0 alcohol unit < 18 years				
		Carbs and fibre-rich products; Bread Recommendation 2 ½ - 4 ½ serving/day				
		Pasta, rice, ecc.. Recommendation 1 – 1 ½ serving/day				
		Fish; Recommendation 2-3 servings/week				
		Eggs; Recommendation 2-4 servings/week				
		Extra olive oil; Recommendation 2-4 servings/day				
		Water; Recommendation 1.5-2L/day				
		Salt; Recommendation <5g/day (<4g/day for elderly group / IT6)				
		limit the consumption of meat, preferring the white type (chicken, rabbit) to the read one (pork, beef)		red meat has the highest environmental impact.		
		Which type of animal products should be preferred: milk and yogurt	they have good nutritional values	lower environmental impact with respect to meat (high quality protein at lower cost than meat)		

		food as chicken, eggs, milk, yogurt, ricotta and legumes:	high nutritional value		low cost	
		Fish: choose the small size species		their consumption is more sustainable due to their low trophic level		
		Choose cooking techniques with low environmental impact (techniques that last less), e.g. with steam or the microwave.				
		Prefer the consumption of seasonal and local food products.				
		Organic products are more sustainable.				
		Try to limit the impact of the food packaging: chooses the product that has less packaging, paying attention to the info reported on the label, choosing the recycling packaging, throwing away the packaging correctly.				
		Reduce household food waste, adopting specific strategies such as planning the shopping, storing correctly and saving the leftovers				
		Ask for an expert counseling if you need to go on a diet				
		if you are overweight eat less and focus on low-energy product				

		if you go on a diet, follow a balanced diet avoiding too strict self-made diets; be careful about the adolescents, that are more likely to be at risk of developing eating disorders or to follow risky diets that aim to drastically lose weight				
		be careful about a vegan diet that, if not correctly followed, can bring to important deficiencies				
		be careful also about the excess of physical exercises, especially among the boys.				
		after cooking, don't leave a meal outside the fridge for more than 2 hours in winter and 1 hour in summer				
		avoiding the contact between the cooked foods and those that will be eaten raw				
		wash carefully fruit and vegetables				
		pay attention to the 'use by' date				
	Childbearing age, conception period, pregnancy IT4	plan the pregnancy, ensuring the right intake of folic acid also before the conception				
		try to arrive at this life stage with an adequate weight that is important to have a healthy pregnancy				
		do not go on a strict diet during this period				
		assure an adequate intake of water, protein, calcium, iron and folate				
		follow hygienic procedures to manage food at home and avoid the risk of contracting infections (e.g. do not eat raw or less cooked products and before consume fresh				

		fruit and vegetables, wash them carefully)				
		do not drink alcohol				
		do not smoke				
	Breastfeeding IT4	nutritional requests are higher than the pregnancy period, for this reason, ensure an adequate fresh fruit and vegetables consumption, fish, milk and dairy products, together with drinking more water than a normal situation (3-4 glasses of water more that those recommended)				
		do not go on a diet during the breastfeeding period				
	Menopause IT5	follow a balanced diet as in the previous period (this means that you should not eliminate any food groups rather than limiting the consumption of some of them)	have in mind that during this period there are different **risk factors**: overweight, obesity, being inactive, smoking, and alcohol			
		do a constant physical activity (including both exercises and the normal movement, e.g. do not be sedentary but active)				
		maintain an adequate intake of calcium through the consumption of partially skimmed milk and yogurt or with small portions of cheese together with small-size fishes and some type of vegetables (e.g. spinach or broccoli)				

		avoid the use of vegetables estrogen				
	Unweaned IT2	choose the milk-formula if you cannot breastfeed				
		continue to breastfeed during the weaning period				
		do not add salt and sugar to the baby meal				
		introduce slowly every kind of food prepared with simple cooking techniques				
		ensure an adequate intake of water also during the meal				
		avoid pushing the baby to eat every kind of food				
		be careful while the baby is eating				
	School kids IT3	encourage the kid to have breakfast every morning with milk/yogurt + bread/biscuits (including sometimes whole-grain products)				
		encourage the kid to have a healthy break at mid-morning and mid-afternoon eating for example fruit, nuts, a slice of bread with cheese/oil				
		encourage the consumption of fruit and vegetables (2-3 portion of fruit and 2 portion of vegetables per day)				
		legumes (2-4 times a week)				
		fish- better if bluefish (2-3 times a week)				
		foods that guarantee an adequate intake of calcium (milk, yogurt and cheese-choosing the yogurt without added sugar)				
		limit the consumption of sweet snacks and sweetened beverages				

		vary the kid diet and do not give him/her always the same choices				
		encourage the kid to enjoy the school lunch				
		make more attractive the taste of some bitter vegetables putting them together with those sweeter				
		encourage the kid to do physical activity: moderate-powerful at least one hour per day; do 3 times a week physical activity that strengths the muscle and bone structure				
		encourage the kid to spend time outdoor doing movement				
	Adolescents IT5	follow a diet rich in plant-based products such as fruit, vegetables and legumes				
		do not follow unbalanced diets				
		especially if you are a girl, ensure an adequate intake of all nutrients, without excluding any type of food to guarantee and adequate intake of iron and calcium				
		prevent the osteoporosis through and adequate intake of products rich in calcium (milk and yogurt: three times per day; cheese: three times per week; small-size fish: three times a week; vegetables and water rich in calcium) together with a frequent physical activity and a correct exposure to sun				
		have a balanced breakfast every day				
		prefer to drink water instead of other beverages				

		limit the consumption of food rich in salt, sugar and fats				
		sleep adequately				
		do a constant physical activity meaning moderate-powerful activity 1 hour per day (e.g. running, walking..) adding at least three times a week a powerful activity that strengthen the muscles and bone structure				
		do not drink alcoholic beverages	due to the health damage			
	The elderly IT6	eat constantly during all day frequent and simple meals				
		in your daily main meal insert fruit, vegetables and one of these protein products as fish, meat, eggs, cheese, or legumes;				
		have a balanced breakfast every day that includes also milk or yogurt (that could be considered also a snack)				
		avoid eating pre-cooked meal that are often rich in salt, sugar and fats				
		do not avoid the consumption of vegetables, legumes and whole-grain products				
		in case of problem related to mastication/swallowing preferring the puree/soups/smoothie form				
		limit the consumption of sugar, sweets and sugary drinks				
		be active walking at least 30 minutes per day outside to help the fixation of Vitamin D				
		add other type of physical activity if you can				

	Sportive/athletes IT9	if you play an amateur sport or do a normal physical activity you should not follow particular recommendations rather than those present in the dietary guidelines				
		if you are a person who abundantly sweat during the physical activity, you should drink water before, during and after that				
		if you are an athlete you should eat more because your energy requirements are higher and also drink more water for your increased requirements in terms of liquid an minerals				
		if you are an athlete speak to experts as nutritionists or doctors to have specific nutritional advice if you do activity 5-7 days per week, each session that lasts 2-3 hours				
		advise for professional athletes to improve their performances: drink water constantly, eat enough quantities of carbohydrates, do not eat foods that can compromise your digestion (as fats and fibers) near the physical activity.				
	non-communicable diseases IT7	ensure an adequate consumption of fresh fruit and vegetables and whole-grain cereals				
		avoid the consumption of trans fatty acids, reduce the use of salt and limit the consumption of red meat, in particular the cured meat				
Greece EL		Consume a variety of fruit and vegetables everyday.				https://www.fao.org/nutrition/education/food-dietary-guidelines/regions/countries/greece/en/

(work-life to retirement, elderly)	General population EL1	Consume a variety of grains everyday. Prefer whole grains.				
		Prefere low fat-dairy products				
		Limit red meat consumption. Choose lean cuts. Avoid processed meat.				
		Consume fish and seafood frequently. Choose small fatty fish.				
		Consume legumes frequently.				
		Use olive oil as the main added fat.				
		Limit salt and added sugar intake.				
		Be physically active every day. Maintain a healthy body weight.				
		Drink plenty of water.				
	Adults EL5	For adults 4 servings of vegetables per day are recommended				
		For adults 3 servings of legumes per week are recommended				
		For adults, 1-2 servings of nuts per day are recommended				
		For adults, 5-8 servings of refined and whole-grain cereals per day are recommended. Of these servings, consumption of potatoes should be 3 servings per week.				
		For adults, 1 serving of lean red meat is recommended per week. Moreover, 1-2 servings of white meat are recommended per week. For both red and white meat, as little processed as possible is recommended				

		For adults, 2-3 servings of fish and seafood per week (variety of fish and seafood) are recommended				
		If an adult consumes alcohol, the recommendation is to prefer consuming wine during meals, up to 2 glasses/per day for men and up to 1 glass/per day for women.				
	infants, children and adolescents EL3 /EL4(parents)	emphasis is placed on the importance of consuming a healthy breakfast, including items from at least three food groups (i.e. dairy products, cereals, and fruits or vegetables)				
		the influence of parental behaviours (e.g. acting as role models) for encouraging healthy dietary patterns: Be a role model for your children by encouraging healthy eating.				
		the importance of consuming family meals: Eat together as a family as frequently as possible. Turn the TV off.				
		Eat 3 main meals and at least one snack every day.				
		Eat breakfast every day.				
		Drink plenty of water				
		Cook at home healthy and safe food				
		Choose seasonal products.				
Planetary health diet PHD	General population PHD1	Eat a healthy diet: Healthy diets have an appropriate caloric intake and consist largely of a diversity of plant foods, low amounts of animal source foods, contain unsaturated rather than	Present dietary trends: The global burden of non-communicab	producing food for nearly 10 billion people should: use no	A transformation to healthy diets from sustainab	Additionally: https://openaccess.city.ac.uk/id/eprint/21633/8/Food%20Planet%20Health.pdf https://eatforum.org/eat-lancet-commission/eat-lancet-commission-summary-report/

		saturated fats, and limited amounts of refined grains, highly processed foods and added sugars.	le diseases is set to worsen -> Dietary changes from current diets towards healthy diets are likely to result in significant health benefits that include averting approximately 7.4 to 10.8 million premature deaths per year, a reduction of between 18% to 28%.	additional land; safeguard existing biodiversity; reduce consumptive water use and manage water responsibly; drastically reduce nitrogen and phosphorus pollution; produce zero carbon dioxide emissions and cause no further increase in methane and nitrous oxide emissions. Present dietary trends: the impacts of food production on greenhouse gas emissions, nitrogen	le food systems is a prerequisite for attaining the UN Sustainable Development Goals and Paris Agreement. This includes eradicating hunger and universal access to high quality primary health care that integrates family planning and education on healthy diets, with the SDGs on freshwater, climate, land, oceans and biodiversity and achieved	

				and phosphorus pollution, biodiversity loss, and water and land use will erode the stability of the Earth system	through a strong commitment to global partnerships and action. Present diets: Nearly 1 billion people in the world lack sufficient food and many more consume an unhealthy diet that contributes to premature death and morbidity	
		a greater than 100% increase in the consumption of healthy foods such as nuts, fruits, vegetables and legumes			However, the changes needed differ greatly by region	
		a greater than 50% reduction in global consumption of unhealthy foods such as red meat and sugar			However, the changes needed differ greatly by region	
		Food waste must be greatly reduced.				

		Protein sources primarily from plants, including soy foods, other legumes, and nuts.	dietary patterns with the following characteristics promote low risk of major chronic disease and overall wellbeing			
		Fish or alternative sources of omega-3 fatty acids several times per week, with optional modest consumption of poultry and eggs.				
		Low intakes of red meat, if any, especially processed meat.				
		Fat largely from unsaturated plant sources, with low intakes of saturated fats; no partially hydrogenated oils				
		Carbohydrates primarily from whole grains with limited intake refined grains and sugar less 5% of energy				
		At least five servings of fruits and vegetables per day, not including potatoes				
		Moderate dairy consumption as an option				
