# Supplementary material for: Identifying healthy and sustainable high-impact eating behaviour in French children aged 6–15 years: a combined multidisciplinary and living lab participatory approach
Source: J Nutr Sci. 2026 May 26;15:e38. doi: 10.1017/jns.2026.10105 (PMC13227139; doi:10.1017/jns.2026.10105)
Supplement: Fardet et al. supplementary material 2 — Fardet et al. supplementary material [file S2048679026101050sup002.pdf]

Supplementary material n°2

**Categories**	**Key behaviour items**	**Children adjustments**	**Young parents adjustments***
Legumes			
	serving: 70 g raw / 125 g cooked)		
	Choose a variety of legumes		
	Choose organically produced legumes		
	Choose regional legumes		
Vegetables and fruits			
	per day (1 serving for an adult diet: 125		
	portions of vegetables / 1-2 portions of		
	Choose a variety of vegetables and fruits		
	and fruits		
	Choose regional vegetables and fruits		
	Choose seasonal vegetables and fruits		
Nuts and seeds			
	)3 times a week (1 serving for an adult		
	Choose a variety of nuts and seeds		
	seeds		
	Choose regional nuts and seeds		
Grains			
	day (1 serving: e.g., 40-60 g bread / 60-80 g dried pasta or dried rice)		
	(e.g., flour types, pasta, rice etc.)		
	Choose primarily whole grains		
	Choose organically produced grains		
	Choose regional grains		
Meat			
	serving for an adult diet: 100-125 g)		
	meat (both red and white meat) or even		
	even avoid it		
	even avoid it		
	meat		
	Choose organically produced meat		
	Choose regional meat		
	an alternative source of protein		
	alternative source of protein		
	alternative source of protein		
	legumes, nuts) instead of meat as an alternative source of protein		
Fish			
	week (1 serving: 125-150g)		

	sardines, anchovies)		
	Choose organically produced fish		
	Choose domestic fish		
Eggs			
	Eat 2-4 servings of eggs per week (1 serving for an adult diet: 1 egg)		
	Choose organically produced eggs		
	Choose regional eggs		
Dairy			
	(for an adult diet e.g., 200 g milk/dairy<br>product and 60 g cheese per day)		
	instead of full-fat milk and dairy products		
	and dairy products		
	dairy products		
	Choose regional milk and dairy products		
Fats, sugar and salt			
	Consume 30-40 g of oils (for an adult diet) per day		
	rapeseed oil)		
	Choose organically produced oils		
	Choose regional oils		
	(e.g., from sweets) (max. 25 g for an<br>adult diet per day)		
	Choose organically produced sugar		
	Eat a max. of 6 g salt (for an adult diet) pe	(babies), eat no added<br>salt in meals	Add no salt to the baby
	Choose iodized salt		
	Choose fluoridated salt		
	products high in salt, sugars and<br>fats (e.g., fast food, salty snacks, biscuits,		
Beverages			
	Drink 1.5-2L water (for an adult diet) per day		
	water		
	sweetened beverages		
	beverages (e.g., tea) instead of sugar-<br>sweetened beverages		
	(e.g., tea, coffee, juice)		
	a maximum of up to 2 glasses/per day for<br>men and up to 1 glass/per day for	Do not drink alcohol at<br>an age under 18 years	you are pregnant or<br>breast feeding
Eating behaviours			
	Eat 3 main meals every day	smaller meals (e.g.<br>have a snack at mid-<br>morning and mid-	according to the<br>baby's needs (if<br>possible)

	Eat breakfast	Accept a variety of foods	acceptance of a variety of foods
		Eat together as frequently as possible	
	eating		
Nutritional needs	accordingly (don't over-/under-eat)		
	needs (e.g. macro and micro nutrients)		
	with reliable ressources		
	Ensure an adequate vitamin-D intake through sun exposure or supplementation (20 µg/d)		folic acid intake through supplementation (400

***Footnote**: The adjustments in this column are intended for other project contexts and are not applicable to the French living lab (children) study. The applicable column for Living Lab France is “Children adjustments”.
