# Supplementary material for: Identifying healthy and sustainable high-impact eating behaviour in French children aged 6–15 years: a combined multidisciplinary and living lab participatory approach
Source: J Nutr Sci. 2026 May 26;15:e38. doi: 10.1017/jns.2026.10105 (PMC13227139; doi:10.1017/jns.2026.10105)
Supplement: Fardet et al. supplementary material 5 — Fardet et al. supplementary material [file S2048679026101050sup005.pdf]

Intro

Dear Project Partner,

The following survey serves the identification of 5 high impact behaviours per LL, which we will continue to work with for the rest of the Plan'Eat project. Your ratings will help to look at a maximum number of potential behaviours, and to distill the most promising ones.

What to expect in the survey
You will be asked to rate a number of behaviours regarding their socio-economic potential.

The socio-economic potential is determined by many different socio-ecomomic indicators (e.g., diet affordability, animal welfare, labour rights). We ask you to keep these indicators in mind when ranking the **overall or average socio-economic potential** of each behaviour across all indicators. I.e., please indicate one socio-economic potential rating per behaviour, taking all indicators you deem important into account.

About the rating: The term socio-economic potential refers to the potential positive impact of the behaviours on socio-ecomomic issues:
The higher the positive socio-economic impact, the higher the potential.

You can also indicate 'no/negative potential' if the behaviour in question overall has no potential at all or rather negative impacts on socio-economic issues.

If there are behaviours that need more elaboration than 'one simple rating'
At the end of the survey there will be a free field for you to elaborate more on single behaviours. If you rate anything you would like to specify or discuss more in-depth, please take a note of it and write it in the free field at the end of the survey.

If there are behaviours not listed in our survey that you would like to rate and discuss
You will be able to name and rate extra behaviours at the end of the survey. Please take note of them for yourself as you go through the survey, and use the space provided in the end to add them.

We estimate that it will take about 15-20 minutes to rate all behaviours. You can take a break and return to the survey at any time.

Thank you for your time and answers! JLU

All the information you provide will be treated strictly confidential. Only members of the team of JLU and EUFIC will have access to the collected data, and it will only be used for the purpose and in the way as stated before. Please note that the information you provide is not anonymous, because you will indicate your organization.

Your decision to participate in this research and your answers are entirely voluntary. Please consent to participating in this survey.

○ Yes, I consent.

What organization are you from?

[▾]

Socio-economic

Please remember the rating: The higher the positive socio-economic impact of a behaviour, the higher the potential.

What is the socio-economic potential of the following behaviours related to **legumes?**

	No/negative potential	Very low potential	Low potential	Medium potential	High potential	Very high potential	Not applicable/Don't know
Eat 3 servings of legumes per week (1 serving for an adult diet: 70 g raw / 125 g cooked)	○	○	○	○	○	○	○
Choose a variety of legumes	○	○	○	○	○	○	○
Choose organically produced legumes	○	○	○	○	○	○	○
Choose regional legumes	○	○	○	○	○	○	○

What is the socio-economic potential of the following behaviours related to **vegetables and fruits?**

	No/negative potential	Very low potential	Low potential	Medium potential	High potential	Very high potential	Not applicable/Don't know
Eat 5 servings of vegetables and fruits per day (1 serving for an adult diet: 125 g)	○	○	○	○	○	○	○
Eat more vegetables than fruits (3-4 portions of vegetables / 1-2 portions of fruit)	○	○	○	○	○	○	○
Choose a variety of vegetables and fruits	○	○	○	○	○	○	○
	No/negative potential	Very low potential	Low potential	Medium potential	High potential	Very high potential	Not applicable/Don't know

	No/negative potential	Very low potential	Low potential	Medium potential	High potential	Very high potential	Not applicable/Don't know
Choose organically produced vegetables and fruits	○	○	○	○	○	○	○
Choose regional vegetables and fruits	○	○	○	○	○	○	○
Choose seasonal vegetables and fruits	○	○	○	○	○	○	○

What is the socio-economic potential of the following behaviours related to **nuts and seeds?**

	No/negative potential	Very low potential	Low potential	Medium potential	High potential	Very high potential	Not applicable/Don't know
Eat a small handful of nuts and seeds (2-)3 times a week (1 serving for an adult diet: 25 g)	○	○	○	○	○	○	○
Choose a variety of nuts and seeds	○	○	○	○	○	○	○
Choose organically produced nuts and seeds	○	○	○	○	○	○	○
Choose regional nuts and seeds	○	○	○	○	○	○	○

What is the socio-economic potential of the following behaviours related to **grains?**

	No/negative potential	Very low potential	Low potential	Medium potential	High potential	Very high potential	Not applicable/Don't know
Eat 3-6 servings of grain-based foods per day (1 serving for an adult diet: e.g., 40-60 g bread / 60-80 g dried pasta or dried rice)	○	○	○	○	○	○	○
Choose a variety of grain-based foods (e.g., flour types, pasta, rice etc.)	○	○	○	○	○	○	○
Choose primarily whole grains	○	○	○	○	○	○	○
Choose organically produced grains	○	○	○	○	○	○	○
Choose regional grains	○	○	○	○	○	○	○

What is the socio-economic potential of the following behaviours related to **meat?**

	No/negative potential	Very low potential	Low potential	Medium potential	High potential	Very high potential	Not applicable/Don't know
Eat 0-3 servings of meat per week (1 serving for an adult diet: 100-125 g)	○	○	○	○	○	○	○
Limit the consumption of processed meat (both red and white meat) or even avoid it	○	○	○	○	○	○	○
Limit the consumption of red meat or even avoid it	○	○	○	○	○	○	○
Limit the consumption of all meats or even avoid it	○	○	○	○	○	○	○

	No/negative potential	Very low potential	Low potential	Medium potential	High potential	Very high potential	Not applicable/Don't know
Choose poultry instead of red/processed meat	○	○	○	○	○	○	○
Choose organically produced meat	○	○	○	○	○	○	○
Choose regional meat	○	○	○	○	○	○	○
Choose dairy products instead of meat as an alternative source of protein	○	○	○	○	○	○	○

	No/negative potential	Very low potential	Low potential	Medium potential	High potential	Very high potential	Not applicable/Don't know
Choose eggs instead of meat as an alternative source of protein	○	○	○	○	○	○	○
Choose fish instead of meat as an alternative source of protein	○	○	○	○	○	○	○
Choose plant-based alternatives (e.g., legumes, nuts) instead of meat as an alternative source of protein	○	○	○	○	○	○	○

What is the socio-economic potential of the following behaviours related to **fish?**

	No/negative potential	Very low potential	Low potential	Medium potential	High potential	Very high potential	Not applicable/Don't know
Eat (2-)3 servings of fish and seafood per week (1 serving for an adult diet: 125-150g)	○	○	○	○	○	○	○
Choose (small) fatty fish (e.g. salmon, sardines, anchovies)	○	○	○	○	○	○	○
Choose organically produced fish	○	○	○	○	○	○	○
Choose domestic fish	○	○	○	○	○	○	○

What is the socio-economic potential of the following behaviours related to **eggs?**

	No/negative potential	Very low potential	Low potential	Medium potential	High potential	Very high potential	Not applicable/Don't know
Eat 2-4 servings of eggs per week (1 serving for an adult diet: 1 egg)	○	○	○	○	○	○	○
Choose organically produced eggs	○	○	○	○	○	○	○
Choose regional eggs	○	○	○	○	○	○	○

What is the socio-economic potential of the following behaviours related to **milk and dairy products?**

	No/negative potential	Very low potential	Low potential	Medium potential	High potential	Very high potential	Not applicable/Don't know
Consume milk and dairy products daily (for an adult diet e.g., 200 g milk/dairy product and 60 g cheese per day)	○	○	○	○	○	○	○
Choose low-fat milk and dairy products instead of full-fat milk and dairy products	○	○	○	○	○	○	○
Choose plant-based alternatives to milk and dairy products	○	○	○	○	○	○	○
	No/negative potential	Very low potential	Low potential	Medium potential	High potential	Very high potential	Not applicable/Don't know

	No/negative potential	Very low potential	Low potential	Medium potential	High potential	Very high potential	Not applicable/Don't know
Choose organically produced milk and dairy products	○	○	○	○	○	○	○
Choose regional milk and dairy products	○	○	○	○	○	○	○

What is the socio-economic potential of the following behaviours related to **fats, sugar, and salt?**

	No/negative potential	Very low potential	Low potential	Medium potential	High potential	Very high potential	Not applicable/Don't know
Consume 30-40 g of oils (for an adult diet) per day	○	○	○	○	○	○	○
Choose vegetable oils (e.g., olive oil, rapeseed oil)	○	○	○	○	○	○	○
Choose organically produced oils	○	○	○	○	○	○	○
Choose regional oils	○	○	○	○	○	○	○

	No/negative potential	Very low potential	Low potential	Medium potential	High potential	Very high potential	Not applicable/Don't know
Limit the consumption of added sugars (e.g., from sweets) (max. 25 g for an adult diet per day)	○	○	○	○	○	○	○
Choose organically produced sugar	○	○	○	○	○	○	○
Eat a max. of 6 g salt (for an adult diet) per day	○	○	○	○	○	○	○
Choose iodized salt	○	○	○	○	○	○	○

	No/negative potential	Very low potential	Low potential	Medium potential	High potential	Very high potential	Not applicable/Don't know
Choose fluoridated salt	○	○	○	○	○	○	○
For young children (babies), eat no added salt in meals	○	○	○	○	○	○	○

	No/negative potential	Very low potential	Low potential	Medium potential	High potential	Very high potential	Not applicable/Don't know
Limit the consumption of processed food products high in salt, sugars, and fats (e.g., fast food, salty snacks, biscuits, bars)	○	○	○	○	○	○	○

What is the socio-economic potential of the following behaviours related to **beverages?**

	No/negative potential	Very low potential	Low potential	Medium potential	High potential	Very high potential	Not applicable/Don't know
Drink 1.5-2L water (for an adult diet) per day	○	○	○	○	○	○	○
Choose tab water instead of bottled water	○	○	○	○	○	○	○
Choose to drink water instead of sugar-sweetened beverages	○	○	○	○	○	○	○
Choose to drink other unsweetened beverages (e.g., tea) instead of sugar-sweetened beverages	○	○	○	○	○	○	○

	No/negative potential	Very low potential	Low potential	Medium potential	High potential	Very high potential	Not applicable/Don't know
Choose organically produced beverages (e.g., tea, coffee, juice)	○	○	○	○	○	○	○
Limit alcoholic beverage consumption to a max. of up to 2 glasses/per day for men and up to 1 glass/per day for women	○	○	○	○	○	○	○
Do not drink alcohol at an age under 18 years	○	○	○	○	○	○	○
Do not drink alcohol if you are pregnant or breast feeding	○	○	○	○	○	○	○

What is the socio-economic potential of the following **eating behaviours?**

	No/negative potential	Very low potential	Low potential	Medium potential	High potential	Very high potential	Not applicable/Don't know
Eat 3 main meals every day	○	○	○	○	○	○	○
Eat more frequent and smaller meals (e.g. have a snack at mid-morning and mid-afternoon)	○	○	○	○	○	○	○
Eat breakfast	○	○	○	○	○	○	○
Accept a variety of foods	○	○	○	○	○	○	○
	No/negative potential	Very low potential	Low potential	Medium potential	High potential	Very high potential	Not applicable/Don't know
Eat together as frequently as possible	○	○	○	○	○	○	○
Feed breast milk according to the baby's needs (if possible)	○	○	○	○	○	○	○
Eat mindfully and allow plenty of time for eating	○	○	○	○	○	○	○

What is the socio-economic potential of the following behaviours related to **nutritional needs?**

	No/negative potential	Very low potential	Low potential	Medium potential	High potential	Very high potential	Not applicable/Don't know
Know your energy (caloric) needs and eat accordingly (don't over-/under-eat)	○	○	○	○	○	○	○
Inform yourself about your nutritional needs (e.g. macro and micro nutrients) with reliable resources	○	○	○	○	○	○	○
Ensure an adequate vitamin-D intake through sun exposure or supplementation (20 µg/d for an adult)	○	○	○	○	○	○	○
Ensure an adequate folic acid intake through supplementation (400 µg/d) if you are of childbearing age	○	○	○	○	○	○	○

Other behaviours

Are there any further behaviours you would like to rate? (e.g. limit the consumption of fish, eat tofu X times a week)

Please name up to three behaviours.
In the following you will again rate the socio-economic potential.

If you do not have any further behaviours, please leave the following text fields empty.

1. Further behaviour
2. Further behaviour
3. Further behaviour

Other behaviours: Environment

What is the socio-economic potential of the following **eating behaviours**?

	No/negative potential	Very low potential	Low potential	Medium potential	High potential	pc
${q://QID1212325209/ChoiceTextEntryValue/1}	○	○	○	○	○	
${q://QID1212325209/ChoiceTextEntryValue/2}	○	○	○	○	○	
${q://QID1212325209/ChoiceTextEntryValue/3}	○	○	○	○	○	

Comments and end survey

Please briefly elaborate which socio-economic indicators you primarily relied on when completing the socio-economic potential rating? Was it the same for each behaviour?

Here you can also use the space to further specify and discuss your ratings if you'd like to.

Please write here if you have any further comments or leave empty and move to the end of the survey!

Powered by Qualtrics
