# Supplementary material for: Identifying healthy and sustainable high-impact eating behaviour in French children aged 6–15 years: a combined multidisciplinary and living lab participatory approach
Source: J Nutr Sci. 2026 May 26;15:e38. doi: 10.1017/jns.2026.10105 (PMC13227139; doi:10.1017/jns.2026.10105)
Supplement: Fardet et al. supplementary material 6 — Fardet et al. supplementary material [file S2048679026101050sup006.pdf]

Intro

Dear LL leaders and representatives,

The following survey serves the identification of 5 high impact behaviours for your LL, which we will continue to work with for the rest of the Plan'Eat project.

For this purpose, we will present you with an extensive list of behaviours. Your ratings will help to look at a maximum number of potential behaviours, and to distill the most promising ones. This survey is a preparation for the following interview, where we will discuss your ratings, any comments you have etc.

What to expect in the survey
Firstly, we will ask you about the most important eating settings (e.g., school, home, hospital) for you and your target group. Secondly, for each setting, you will be asked to rate a number of behaviours regarding two things:

- a) How likely is it that your target group would adopt this behaviour?
- b) How likely is it that relevant stakeholder (e.g., teachers, parents, employers, canteen providers) would support your target group with adopting this behaviour?

The time the survey takes depends on the number of eating settings you indicate. We estimate that it will take about 10-15 minutes to rate all behaviours in one setting. You can take a break and return to the survey at any time.

If there are behaviours not listed in our survey that you would like to rate and discuss
You will be able to name and rate extra behaviours at the end of the survey. Please take note of them for yourself as you go through the survey, and use the space provided in the end to add them.

Thank you for your time and answers! JLU

All the information you provide will be treated strictly confidential. Only members of the team of JLU and EUFIC will have access to the collected data, and it will only be used for the purpose and in the way as stated before. Please note that the information you provide is not anonymous, because we will discuss your answers in the interview.

Your decision to participate in this research and your answers are entirely voluntary. Please consent to participating in this survey.

- ○ Yes, I consent.

Settings

Which is your living lab?

What is your target group? You can select multiple if applicable.

- [ ] Young children (under 6 years)
- [ ] Children and adolescents (6-18 years)
- [ ] Adult population (18 years and older)
- [ ] Pregnant women & young parents
- [ ] Elderly (60 years and older)

What are the *most relevant* eating settings for you and your target group (e.g. school, home, hospital)?

Important: Please name at least one setting before you click next!
If you do not have specific settings, please write 'general' under '1. Setting' and leave the others empty.

You can name up to three settings. In the following you will rate each behaviour for each setting separately.

1. Setting
2. Setting
3. Setting

Important

For the behaviours we indicate standard servings per day/week for an adult diet. If your target group is different (e.g., children, adolescents or elderly), please in your head adapt these serving sizes to what is adequate for your target group.

I.e., the survey question will contain the adult serving as an example, but please answer the question with the equivalent adequate serving size for your target group in mind.

Setting 1 Plasticity

We will now ask you to rate all behaviours in the first setting you indicated:

${q://QID7/ChoiceTextEntryValue/1}

How likely is it that your target group would adopt the following behaviours related to **legumes** in the following setting: **${q://QID7/ChoiceTextEntryValue/1}**

	Target group cannot do this	Very unlikely	Unlikely	Somewhat likely	Likely	Very likely	Target group already does this	Not applicable/I don't know
Eat 3 servings of legumes per week (1 serving for an adult diet: 70 g raw / 125 g cooked)	○	○	○	○	○	○	○	○
Choose a variety of legumes	○	○	○	○	○	○	○	○
Choose organically produced legumes	○	○	○	○	○	○	○	○
Choose regional legumes	○	○	○	○	○	○	○	○

How likely is it that your target group would adopt the following behaviours related to **vegetables and fruits** in the following setting: **${q://QID7/ChoiceTextEntryValue/1}**

	Target group cannot do this	Very unlikely	Unlikely	Somewhat likely	Likely	Very likely	Target group already does this	Not applicable/I don't know
Eat 5 servings of vegetables and fruits per day (1 serving for an adult diet: 125 g)	○	○	○	○	○	○	○	○
Eat more vegetables than fruits (3-4 portions of vegetables / 1-2 portions of fruit)	○	○	○	○	○	○	○	○
Choose a variety of vegetables and fruits	○	○	○	○	○	○	○	○

	Target group cannot do this	Very unlikely	Unlikely	Somewhat likely	Likely	Very likely	Target group already does this	Not applicable/I don't know
Choose organically produced vegetables and fruits	○	○	○	○	○	○	○	○
Choose regional vegetables and fruits	○	○	○	○	○	○	○	○
Choose seasonal vegetables and fruits	○	○	○	○	○	○	○	○

How likely is it that your target group would adopt the following behaviours related to **nuts and seeds** in the following setting: **${q://QID7/ChoiceTextEntryValue/1}**

	Target group cannot do this	Very unlikely	Unlikely	Somewhat likely	Likely	Very likely	Target group already does this	Not applicable/I don't know
Eat a small handful of nuts and seeds (2-)3 times a week (1 serving for an adult diet: 25 g)	○	○	○	○	○	○	○	○
Choose a variety of nuts and seeds	○	○	○	○	○	○	○	○
Choose organically produced nuts and seeds	○	○	○	○	○	○	○	○
Choose regional nuts and seeds	○	○	○	○	○	○	○	○

How likely is it that your target group would adopt the following behaviours related to **grains** in the following setting: **${q://QID7/ChoiceTextEntryValue/1}**

	Target group cannot do this	Very unlikely	Unlikely	Somewhat likely	Likely	Very likely	Target group already does this	Not applicable/I don't know
Eat 3-6 servings of grain-based foods per day (1 serving for an adult diet: e.g., 40-60 g bread / 60-80 g dried pasta or dried rice)	○	○	○	○	○	○	○	○
Choose a variety of grain-based foods (e.g., flour types, pasta, rice etc.)	○	○	○	○	○	○	○	○
Choose primarily whole grains	○	○	○	○	○	○	○	○
Choose organically produced grains	○	○	○	○	○	○	○	○
Choose regional grains	○	○	○	○	○	○	○	○

How likely is it that your target group would adopt the following behaviours related to **meat** in the following setting: **${q://QID7/ChoiceTextEntryValue/1}**

	Target group cannot do this	Very unlikely	Unlikely	Somewhat likely	Likely	Very likely	Target group already does this	Not applicable/I don't know
Eat 0-3 servings of meat per week (1 serving for an adult diet: 100-125 g)	○	○	○	○	○	○	○	○

	Target group cannot do this	Very unlikely	Unlikely	Somewhat likely	Likely	Very likely	Target group already does this	Not applicable/I don't know
Limit the consumption of processed meat (both red and white meat) or even avoid it	○	○	○	○	○	○	○	○
Limit the consumption of red meat or even avoid it	○	○	○	○	○	○	○	○
Limit the consumption of all meats or even avoid it	○	○	○	○	○	○	○	○

	Target group cannot do this	Very unlikely	Unlikely	Somewhat likely	Likely	Very likely	Target group already does this	Not applicable/I don't know
Choose poultry instead of red/processed meat	○	○	○	○	○	○	○	○
Choose organically produced meat	○	○	○	○	○	○	○	○
Choose regional meat	○	○	○	○	○	○	○	○
Choose dairy products instead of meat as an alternative source of protein	○	○	○	○	○	○	○	○

	Target group cannot do this	Very unlikely	Unlikely	Somewhat likely	Likely	Very likely	Target group already does this	Not applicable/I don't know
Choose eggs instead of meat as an alternative source of protein	○	○	○	○	○	○	○	○
Choose fish instead of meat as an alternative source of protein	○	○	○	○	○	○	○	○
Choose plant-based alternatives (e.g., legumes, nuts) instead of meat as an alternative source of protein	○	○	○	○	○	○	○	○

How likely is it that your target group would adopt the following behaviours related to **fish** in the following setting: **${q://QID7/ChoiceTextEntryValue/1}**

	Target group cannot do this	Very unlikely	Unlikely	Somewhat likely	Likely	Very likely	Target group already does this	Not applicable/I don't know
Eat (2-)3 servings of fish and seafood per week (1 serving for an adult diet: 125-150g)	○	○	○	○	○	○	○	○
Choose (small) fatty fish (e.g. salmon, sardines, anchovies)	○	○	○	○	○	○	○	○
Choose organically produced fish	○	○	○	○	○	○	○	○
Choose domestic fish	○	○	○	○	○	○	○	○

How likely is it that your target group would adopt the following behaviours related to **eggs** in the following setting: **${q://QID7/ChoiceTextEntryValue/1}**

	Target group cannot do this	Very unlikely	Unlikely	Somewhat likely	Likely	Very likely	Target group already does this	Not applicable/I don't know
Eat 2-4 servings of eggs per week (1 serving for an adult diet: 1 egg)	○	○	○	○	○	○	○	○
Choose organically produced eggs	○	○	○	○	○	○	○	○
Choose regional eggs	○	○	○	○	○	○	○	○

How likely is it that your target group would adopt the following behaviours related to **milk and dairy products** in the following setting: **${q://QID7/ChoiceTextEntryValue/1}**

	Target group cannot do this	Very unlikely	Unlikely	Somewhat likely	Likely	Very likely	Target group already does this	Not applicable/I don't know
Consume milk and dairy products daily (for an adult diet e.g., 200 g milk/dairy product and 60 g cheese per day)	○	○	○	○	○	○	○	○
Choose low-fat milk and dairy products instead of full-fat milk and dairy products	○	○	○	○	○	○	○	○
Choose plant-based alternatives to milk and dairy products	○	○	○	○	○	○	○	○
Choose organically produced milk and dairy products	○	○	○	○	○	○	○	○

	Target group cannot do this	Very unlikely	Unlikely	Somewhat likely	Likely	Very likely	Target group already does this	Not applicable/I don't know
Choose regional milk and dairy products	○	○	○	○	○	○	○	○

How likely is it that your target group would adopt the following behaviours related to **fats, sugar, and salt** in the following setting: **${q://QID7/ChoiceTextEntryValue/1}**

	Target group cannot do this	Very unlikely	Unlikely	Somewhat likely	Likely	Very likely	Target group already does this	Not applicable/I don't know
Consume 30-40 g of oils (for an adult diet) per day	○	○	○	○	○	○	○	○
Choose vegetable oils (e.g., olive oil, rapeseed oil)	○	○	○	○	○	○	○	○
Choose organically produced oils	○	○	○	○	○	○	○	○
Choose regional oils	○	○	○	○	○	○	○	○

	Target group cannot do this	Very unlikely	Unlikely	Somewhat likely	Likely	Very likely	Target group already does this	Not applicable/I don't know
Limit the consumption of added sugars (e.g., from sweets) (max. 25 g for an adult diet per day)	○	○	○	○	○	○	○	○
Choose organically produced sugar	○	○	○	○	○	○	○	○
Eat a max. of 6 g salt (for an adult diet) per day	○	○	○	○	○	○	○	○
Choose iodized salt	○	○	○	○	○	○	○	○

	Target group cannot do this	Very unlikely	Unlikely	Somewhat likely	Likely	Very likely	Target group already does this	Not applicable/I don't know
Choose fluoridated salt	○	○	○	○	○	○	○	○
For young children (babies), eat no added salt in meals	○	○	○	○	○	○	○	○
Add no salt to the baby meal	○	○	○	○	○	○	○	○

	Target group cannot do this	Very unlikely	Unlikely	Somewhat likely	Likely	Very likely	Target group already does this	Not applicable/I don't know
Limit the consumption of processed food products high in salt, sugars, and fats (e.g., fast food, salty snacks, biscuits, bars)	○	○	○	○	○	○	○	○

How likely is it that your target group would adopt the following behaviours related to **beverages** in the following setting: **${q://QID7/ChoiceTextEntryValue/1}**

	Target group cannot do this	Very unlikely	Unlikely	Somewhat likely	Likely	Very likely	Target group already does this	Not applicable/I don't know
Drink 1.5-2L water (for an adult diet) per day	○	○	○	○	○	○	○	○
Choose tab water instead of bottled water	○	○	○	○	○	○	○	○
Choose to drink water instead of sugar-sweetened beverages	○	○	○	○	○	○	○	○
Choose to drink other unsweetened beverages (e.g., tea) instead of sugar-sweetened beverages	○	○	○	○	○	○	○	○

	Target group cannot do this	Very unlikely	Unlikely	Somewhat likely	Likely	Very likely	Target group already does this	Not applicable/I don't know
Choose organically produced beverages (e.g., tea, coffee, juice)	○	○	○	○	○	○	○	○
Limit alcoholic beverage consumption to a max. of up to 2 glasses/per day for men and up to 1 glass/per day for women	○	○	○	○	○	○	○	○
Do not drink alcohol at an age under 18 years	○	○	○	○	○	○	○	○
Do not drink alcohol if you are pregnant or breast feeding	○	○	○	○	○	○	○	○

How likely is it that your target group would adopt the following **eating behaviours** in the following setting: **${q://QID7/ChoiceTextEntryValue/1}**

	Target group cannot do this	Very unlikely	Unlikely	Somewhat likely	Likely	Very likely	Target group already does this	Not applicable/I don't know
Eat 3 main meals every day	○	○	○	○	○	○	○	○
Eat more frequent and smaller meals (e.g. have a snack at mid-morning and mid-afternoon)	○	○	○	○	○	○	○	○
Eat breakfast	○	○	○	○	○	○	○	○
Accept a variety of foods	○	○	○	○	○	○	○	○
Encourage the acceptance of a variety of foods	○	○	○	○	○	○	○	○
Eat together as frequently as possible	○	○	○	○	○	○	○	○
Feed breast milk according to the baby's needs (if possible)	○	○	○	○	○	○	○	○
Eat mindfully and allow plenty of time for eating	○	○	○	○	○	○	○	○

How likely is it that your target group would adopt the following behaviours related to **nutritional needs** in the following setting: **${q://QID7/ChoiceTextEntryValue/1}**

	Target group cannot do this	Very unlikely	Unlikely	Somewhat likely	Likely	Very likely	Target group already does this	Not applicable/I don't know
Know your energy (caloric) needs and eat accordingly (don't over-/under-eat)	○	○	○	○	○	○	○	○
Inform yourself about your nutritional needs (e.g. macro and micro nutrients) with reliable resources	○	○	○	○	○	○	○	○
Ensure an adequate vitamin-D intake through sun exposure or supplementation (20 µg/d for an adult)	○	○	○	○	○	○	○	○
Ensure an adequate folic acid intake through supplementation (400 µg/d) if you are of childbearing age	○	○	○	○	○	○	○	○

Setting 1 Feasibility

How likely is it that relevant stakeholders would support your target group with adopting the following behaviours related to **legumes** in the following setting: **${q://QID7/ChoiceTextEntryValue/1}**

	Stakeholders can't support this	Very unlikely	Unlikely	Somewhat likely	Likely	Very likely	Stakeholders already support this	Not applicable/Don't know
Eat 3 servings of legumes per week (1 serving for an adult diet: 70 g raw / 125 g cooked)	○	○	○	○	○	○	○	○
Choose a variety of legumes	○	○	○	○	○	○	○	○
Choose organically produced legumes	○	○	○	○	○	○	○	○
Choose regional legumes	○	○	○	○	○	○	○	○

How likely is it that relevant stakeholders would support your target group with adopting the following behaviours related to **vegetables and fruits** in the following setting: **${q://QID7/ChoiceTextEntryValue/1}**

	Stakeholders can't support this	Very unlikely	Unlikely	Somewhat likely	Likely	Very likely	Stakeholders already support this	Not applicable/Don't know
Eat 5 servings of vegetables and fruits per day (1 serving for an adult diet: 125 g)	○	○	○	○	○	○	○	○
Eat more vegetables than fruits (3-4 portions of vegetables / 1-2 portions of fruit)	○	○	○	○	○	○	○	○
Choose a variety of vegetables and fruits	○	○	○	○	○	○	○	○
	Stakeholders can't support this	Very unlikely	Unlikely	Somewhat likely	Likely	Very likely	Stakeholders already support this	Not applicable/Don't know

	Stakeholders can't support this	Very unlikely	Unlikely	Somewhat likely	Likely	Very likely	Stakeholders already support this	Not applicable/Don't know
Choose organically produced vegetables and fruits								
Choose regional vegetables and fruits								
Choose seasonal vegetables and fruits								

How likely is it that relevant stakeholders would support your target group with adopting the following behaviours related to **nuts and seeds** in the following setting: **${q://QID7/ChoiceTextEntryValue/1}**

	Stakeholders can't support this	Very unlikely	Unlikely	Somewhat likely	Likely	Very likely	Stakeholders already support this	Not applicable/Don't know
Eat a small handful of nuts and seeds (2-)3 times a week (1 serving for an adult diet: 25 g)								
Choose a variety of nuts and seeds								
Choose organically produced nuts and seeds								
Choose regional nuts and seeds								

How likely is it that relevant stakeholders would support your target group with adopting the following behaviours related to **grains** in the following setting: **${q://QID7/ChoiceTextEntryValue/1}**

	Stakeholders can't support this	Very unlikely	Unlikely	Somewhat likely	Likely	Very likely	Stakeholders already support this	Not applicable/Don't know
Eat 3-6 servings of grain-based foods per day (1 serving for an adult diet: e.g., 40-60 g bread / 60-80 g dried pasta or dried rice)	○	○	○	○	○	○	○	○
Choose a variety of grain-based foods (e.g., flour types, pasta, rice etc.)	○	○	○	○	○	○	○	○
Choose primarily whole grains	○	○	○	○	○	○	○	○
Choose organically produced grains	○	○	○	○	○	○	○	○
Choose regional grains	○	○	○	○	○	○	○	○

How likely is it that relevant stakeholders would support your target group with adopting the following behaviours related to **meat** in the following setting: **${q://QID7/ChoiceTextEntryValue/1}**

	Stakeholders can't support this	Very unlikely	Unlikely	Somewhat likely	Likely	Very likely	Stakeholders already support this	Not applicable/Don't know
Eat 0-3 servings of meat per week (1 serving for an adult diet: 100-125 g)	○	○	○	○	○	○	○	○
Limit the consumption of processed meat (both red and white meat) or even avoid it	○	○	○	○	○	○	○	○
Limit the consumption of red meat or even avoid it	○	○	○	○	○	○	○	○

	Stakeholders can't support this	Very unlikely	Unlikely	Somewhat likely	Likely	Very likely	Stakeholders already support this	Not applicable/Don't know
Limit the consumption of all meats or even avoid it	◯	◯	◯	◯	◯	◯	◯	◯

	Stakeholders can't support this	Very unlikely	Unlikely	Somewhat likely	Likely	Very likely	Stakeholders already support this	Not applicable/Don't know
Choose poultry instead of red/processed meat	◯	◯	◯	◯	◯	◯	◯	◯
Choose organically produced meat	◯	◯	◯	◯	◯	◯	◯	◯
Choose regional meat	◯	◯	◯	◯	◯	◯	◯	◯
Choose dairy products instead of meat as an alternative source of protein	◯	◯	◯	◯	◯	◯	◯	◯

	Stakeholders can't support this	Very unlikely	Unlikely	Somewhat likely	Likely	Very likely	Stakeholders already support this	Not applicable/Don't know
Choose eggs instead of meat as an alternative source of protein	◯	◯	◯	◯	◯	◯	◯	◯
Choose fish instead of meat as an alternative source of protein	◯	◯	◯	◯	◯	◯	◯	◯
Choose plant-based alternatives (e.g., legumes, nuts) instead of meat as an alternative source of protein	◯	◯	◯	◯	◯	◯	◯	◯

How likely is it that relevant stakeholders would support your target group with adopting the following behaviours related to **fish** in the following setting: **${q://QID7/ChoiceTextEntryValue/1}**

	Stakeholders can't support this	Very unlikely	Unlikely	Somewhat likely	Likely	Very likely	Stakeholders already support this	Not applicable/Don't know
Eat (2-)3 servings of fish and seafood per week (1 serving for an adult diet: 125-150g)	○	○	○	○	○	○	○	○
Choose (small) fatty fish (e.g. salmon, sardines, anchovies)	○	○	○	○	○	○	○	○
Choose organically produced fish	○	○	○	○	○	○	○	○
Choose domestic fish	○	○	○	○	○	○	○	○

How likely is it that relevant stakeholders would support your target group with adopting the following behaviours related to **eggs** in the following setting: **${q://QID7/ChoiceTextEntryValue/1}**

	Stakeholders can't support this	Very unlikely	Unlikely	Somewhat likely	Likely	Very likely	Stakeholders already support this	Not applicable/Don't know
Eat 2-4 servings of eggs per week (1 serving for an adult diet: 1 egg)	○	○	○	○	○	○	○	○
Choose organically produced eggs	○	○	○	○	○	○	○	○
Choose regional eggs	○	○	○	○	○	○	○	○

How likely is it that relevant stakeholders would support your target group with adopting the following behaviours related to **milk and dairy products** in the following setting: **${q://QID7/ChoiceTextEntryValue/1}**

	Stakeholders can't support this	Very unlikely	Unlikely	Somewhat likely	Likely	Very likely	Stakeholders already support this	Not applicable/Don't know
Consume milk and dairy products daily (for an adult diet e.g., 200 g milk/dairy product and 60 g cheese per day)	○	○	○	○	○	○	○	○
Choose low-fat milk and dairy products instead of full-fat milk and dairy products	○	○	○	○	○	○	○	○
Choose plant-based alternatives to milk and dairy products	○	○	○	○	○	○	○	○
Choose organically produced milk and dairy products	○	○	○	○	○	○	○	○
Choose regional milk and dairy products	○	○	○	○	○	○	○	○

How likely is it that relevant stakeholders would support your target group with adopting the following behaviours related to **fats, sugar, and salt** in the following setting: **${q://QID7/ChoiceTextEntryValue/1}**

	Stakeholders can't support this	Very unlikely	Unlikely	Somewhat likely	Likely	Very likely	Stakeholders already support this	Not applicable/Don't know
Consume 30-40 g of oils (for an adult diet) per day	○	○	○	○	○	○	○	○
Choose vegetable oils (e.g., olive oil, rapeseed oil)	○	○	○	○	○	○	○	○

	Stakeholders can't support this	Very unlikely	Unlikely	Somewhat likely	Likely	Very likely	Stakeholders already support this	Not applicable/Don't know
Choose organically produced oils	○	○	○	○	○	○	○	○
Choose regional oils	○	○	○	○	○	○	○	○
	Stakeholders can't support this	Very unlikely	Unlikely	Somewhat likely	Likely	Very likely	Stakeholders already support this	Not applicable/Don't know
Limit the consumption of added sugars (e.g., from sweets) (max. 25 g for an adult diet per day)	○	○	○	○	○	○	○	○
Choose organically produced sugar	○	○	○	○	○	○	○	○
Eat a max. of 6 g salt (for an adult diet) per day	○	○	○	○	○	○	○	○
Choose iodized salt	○	○	○	○	○	○	○	○
	Stakeholders can't support this	Very unlikely	Unlikely	Somewhat likely	Likely	Very likely	Stakeholders already support this	Not applicable/Don't know
Choose fluoridated salt	○	○	○	○	○	○	○	○
For young children (babies), eat no added salt in meals	○	○	○	○	○	○	○	○
Add no salt to the baby meal	○	○	○	○	○	○	○	○
Limit the consumption of processed food products high in salt, sugars, and fats (e.g., fast food, salty snacks, biscuits, bars)	○	○	○	○	○	○	○	○

How likely is it that relevant stakeholders would support your target group with adopting the following behaviours related to **beverages** in the following setting: **${q://QID7/ChoiceTextEntryValue/1}**

	Stakeholders can't support this	Very unlikely	Unlikely	Somewhat likely	Likely	Very likely	Stakeholders already support this	Not applicable/Don't know
Drink 1.5-2L water (for an adult diet) per day	○	○	○	○	○	○	○	○
Choose tab water instead of bottled water	○	○	○	○	○	○	○	○
Choose to drink water instead of sugar-sweetened beverages	○	○	○	○	○	○	○	○
Choose to drink other unsweetened beverages (e.g., tea) instead of sugar-sweetened beverages	○	○	○	○	○	○	○	○
	Stakeholders can't support this	Very unlikely	Unlikely	Somewhat likely	Likely	Very likely	Stakeholders already support this	Not applicable/Don't know
Choose organically produced beverages (e.g., tea, coffee, juice)	○	○	○	○	○	○	○	○
Limit alcoholic beverage consumption to a max. of up to 2 glasses/per day for men and up to 1 glass/per day for women	○	○	○	○	○	○	○	○
Do not drink alcohol at an age under 18 years	○	○	○	○	○	○	○	○
Do not drink alcohol if you are pregnant or breast feeding	○	○	○	○	○	○	○	○

How likely is it that relevant stakeholders would support your target group with adopting the following **eating behaviours** in the following setting: **${q://QID7/ChoiceTextEntryValue/1}**

	Stakeholders can't support this	Very unlikely	Unlikely	Somewhat likely	Likely	Very likely	Stakeholders already support this	Not applicable/Don't know
Eat 3 main meals every day	○	○	○	○	○	○	○	○
Eat more frequent and smaller meals (e.g. have a snack at mid-morning and mid-afternoon)	○	○	○	○	○	○	○	○
Eat breakfast	○	○	○	○	○	○	○	○
Accept a variety of foods	○	○	○	○	○	○	○	○
	Stakeholders can't support this	Very unlikely	Unlikely	Somewhat likely	Likely	Very likely	Stakeholders already support this	Not applicable/Don't know
Encourage the acceptance of a variety of foods	○	○	○	○	○	○	○	○
Eat together as frequently as possible	○	○	○	○	○	○	○	○
Feed breast milk according to the baby's needs (if possible)	○	○	○	○	○	○	○	○
Eat mindfully and allow plenty of time for eating	○	○	○	○	○	○	○	○

How likely is it that relevant stakeholders would support your target group with adopting the following behaviours related to **nutritional needs** in the following setting: **${q://QID7/ChoiceTextEntryValue/1}**

	Stakeholders can't support this	Very unlikely	Unlikely	Somewhat likely	Likely	Very likely	Stakeholders already support this	Not applicable/Dc know
Know your energy (caloric) needs and eat accordingly (don't over-/under-eat)	○	○	○	○	○	○	○	○

	Stakeholders can't support this	Very unlikely	Unlikely	Somewhat likely	Likely	Very likely	Stakeholders already support this	Not applicable/Dc know
Inform yourself about your nutritional needs (e.g. macro and micro nutrients) with reliable resources	○	○	○	○	○	○	○	○
Ensure an adequate vitamin-D intake through sun exposure or supplementation (20 µg/d for an adult)	○	○	○	○	○	○	○	○
Ensure an adequate folic acid intake through supplementation (400 µg/d) if you are of childbearing age	○	○	○	○	○	○	○	○

Setting 2 Plasticity

We will now ask you to rate all behaviours in the second setting you indicated:

${q://QID7/ChoiceTextEntryValue/2}

How likely is it that your target group would adopt the following behaviours related to **legumes** in the following setting: **${q://QID7/ChoiceTextEntryValue/2}**

	Target group cannot do this	Very unlikely	Unlikely	Somewhat likely	Likely	Very likely	Target group already does this	Not applicable/I don't know
Eat 3 servings of legumes per week (1 serving for an adult diet: 70 g raw / 125 g cooked)	○	○	○	○	○	○	○	○
Choose a variety of legumes	○	○	○	○	○	○	○	○
Choose organically produced legumes	○	○	○	○	○	○	○	○
Choose regional legumes	○	○	○	○	○	○	○	○

How likely is it that your target group would adopt the following behaviours related to **vegetables and fruits** in the following setting: **${q://QID7/ChoiceTextEntryValue/2}**

	Target group cannot do this	Very unlikely	Unlikely	Somewhat likely	Likely	Very likely	Target group already does this	Not applicable/I don't know
Eat 5 servings of vegetables and fruits per day (1 serving for an adult diet: 125 g)	○	○	○	○	○	○	○	○
Eat more vegetables than fruits (3-4 portions of vegetables / 1-2 portions of fruit)	○	○	○	○	○	○	○	○
Choose a variety of vegetables and fruits	○	○	○	○	○	○	○	○

	Target group cannot do this	Very unlikely	Unlikely	Somewhat likely	Likely	Very likely	Target group already does this	Not applicable/I don't know
Choose organically produced vegetables and fruits	○	○	○	○	○	○	○	○
Choose regional vegetables and fruits	○	○	○	○	○	○	○	○
Choose seasonal vegetables and fruits	○	○	○	○	○	○	○	○

How likely is it that your target group would adopt the following behaviours related to **nuts and seeds** in the following setting: **${q://QID7/ChoiceTextEntryValue/2}**

	Target group cannot do this	Very unlikely	Unlikely	Somewhat likely	Likely	Very likely	Target group already does this	Not applicable/I don't know
Eat a small handful of nuts and seeds (2-)3 times a week (1 serving for an adult diet: 25 g)	○	○	○	○	○	○	○	○
Choose a variety of nuts and seeds	○	○	○	○	○	○	○	○
Choose organically produced nuts and seeds	○	○	○	○	○	○	○	○
Choose regional nuts and seeds	○	○	○	○	○	○	○	○

How likely is it that your target group would adopt the following behaviours related to **grains** in the following setting: **${q://QID7/ChoiceTextEntryValue/2}**

	Target group cannot do this	Very unlikely	Unlikely	Somewhat likely	Likely	Very likely	Target group already does this	Not applicable/I don't know
Eat 3-6 servings of grain-based foods per day (1 serving for an adult diet: e.g., 40-60 g bread / 60-80 g dried pasta or dried rice)	○	○	○	○	○	○	○	○
Choose a variety of grain-based foods (e.g., flour types, pasta, rice etc.)	○	○	○	○	○	○	○	○
Choose primarily whole grains	○	○	○	○	○	○	○	○
Choose organically produced grains	○	○	○	○	○	○	○	○
Choose regional grains	○	○	○	○	○	○	○	○

How likely is it that your target group would adopt the following behaviours related to **meat** in the following setting: **${q://QID7/ChoiceTextEntryValue/2}**

	Target group cannot do this	Very unlikely	Unlikely	Somewhat likely	Likely	Very likely	Target group already does this	Not applicable/I don't know
Eat 0-3 servings of meat per week (1 serving for an adult diet: 100-125 g)	○	○	○	○	○	○	○	○
Limit the consumption of processed meat (both red and white meat) or even avoid it	○	○	○	○	○	○	○	○
Limit the consumption of red meat or even avoid it	○	○	○	○	○	○	○	○
Limit the consumption of all meats or even avoid it	○	○	○	○	○	○	○	○

	Target group cannot do this	Very unlikely	Unlikely	Somewhat likely	Likely	Very likely	Target group already does this	Not applicable/I don't know
Choose poultry instead of red/processed meat	○	○	○	○	○	○	○	○
Choose organically produced meat	○	○	○	○	○	○	○	○
Choose regional meat	○	○	○	○	○	○	○	○

	Target group cannot do this	Very unlikely	Unlikely	Somewhat likely	Likely	Very likely	Target group already does this	Not applicable/I don't know
Choose dairy products instead of meat as an alternative source of protein	○	○	○	○	○	○	○	○

	Target group cannot do this	Very unlikely	Unlikely	Somewhat likely	Likely	Very likely	Target group already does this	Not applicable/I don't know
Choose eggs instead of meat as an alternative source of protein	○	○	○	○	○	○	○	○
Choose fish instead of meat as an alternative source of protein	○	○	○	○	○	○	○	○
Choose plant-based alternatives (e.g., legumes, nuts) instead of meat as an alternative source of protein	○	○	○	○	○	○	○	○

How likely is it that your target group would adopt the following behaviours related to **fish** in the following setting: **${q://QID7/ChoiceTextEntryValue/2}**

	Target group cannot do this	Very unlikely	Unlikely	Somewhat likely	Likely	Very likely	Target group already does this	Not applicable/I don't know
Eat (2-)3 servings of fish and seafood per week (1 serving for an adult diet: 125-150g)	○	○	○	○	○	○	○	○
Choose (small) fatty fish (e.g. salmon, sardines, anchovies)	○	○	○	○	○	○	○	○
Choose organically produced fish	○	○	○	○	○	○	○	○
Choose domestic fish	○	○	○	○	○	○	○	○

How likely is it that your target group would adopt the following behaviours related to **eggs** in the following setting: **${q://QID7/ChoiceTextEntryValue/2}**

	Target group cannot do this	Very unlikely	Unlikely	Somewhat likely	Likely	Very likely	Target group already does this	Not applicable/I don't know
Eat 2-4 servings of eggs per week (1 serving for an adult diet: 1 egg)	○	○	○	○	○	○	○	○
Choose organically produced eggs	○	○	○	○	○	○	○	○
Choose regional eggs	○	○	○	○	○	○	○	○

How likely is it that your target group would adopt the following behaviours related to **milk and dairy products** in the following setting: **${q://QID7/ChoiceTextEntryValue/2}**

	Target group cannot do this	Very unlikely	Unlikely	Somewhat likely	Likely	Very likely	Target group already does this	Not applicable/I don't know
Consume milk and dairy products daily (for an adult diet e.g., 200 g milk/dairy product and 60 g cheese per day)	○	○	○	○	○	○	○	○
Choose low-fat milk and dairy products instead of full-fat milk and dairy products	○	○	○	○	○	○	○	○
Choose plant-based alternatives to milk and dairy products	○	○	○	○	○	○	○	○
Choose organically produced milk and dairy products	○	○	○	○	○	○	○	○
Choose regional milk and dairy products	○	○	○	○	○	○	○	○

How likely is it that your target group would adopt the following behaviours related to **fats, sugar, and salt** in the following setting: **${q://QID7/ChoiceTextEntryValue/2}**

	Target group cannot do this	Very unlikely	Unlikely	Somewhat likely	Likely	Very likely	Target group already does this	Not applicable/I don't know
Consume 30-40 g of oils (for an adult diet) per day	○	○	○	○	○	○	○	○
Choose vegetable oils (e.g., olive oil, rapeseed oil)	○	○	○	○	○	○	○	○
Choose organically produced oils	○	○	○	○	○	○	○	○
Choose regional oils	○	○	○	○	○	○	○	○

	Target group cannot do this	Very unlikely	Unlikely	Somewhat likely	Likely	Very likely	Target group already does this	Not applicable/I don't know
	Target group cannot do this	Very unlikely	Unlikely	Somewhat likely	Likely	Very likely	Target group already does this	Not applicable/I don't know
Limit the consumption of added sugars (e.g., from sweets) (max. 25 g for an adult diet per day)	○	○	○	○	○	○	○	○
Choose organically produced sugar	○	○	○	○	○	○	○	○
Eat a max. of 6 g salt (for an adult diet) per day	○	○	○	○	○	○	○	○
Choose iodized salt	○	○	○	○	○	○	○	○
	Target group cannot do this	Very unlikely	Unlikely	Somewhat likely	Likely	Very likely	Target group already does this	Not applicable/I don't know
Choose fluoridated salt	○	○	○	○	○	○	○	○
For young children (babies), eat no added salt in meals	○	○	○	○	○	○	○	○
Add no salt to the baby meal	○	○	○	○	○	○	○	○
Limit the consumption of processed food products high in salt, sugars, and fats (e.g., fast food, salty snacks, biscuits, bars)	○	○	○	○	○	○	○	○

How likely is it that your target group would adopt the following behaviours related to **beverages** in the following setting: **${q://QID7/ChoiceTextEntryValue/2}**

	Target group cannot do this	Very unlikely	Unlikely	Somewhat likely	Likely	Very likely	Target group already does this	Not applicable/I don't know
Drink 1.5-2L water (for an adult diet) per day	○	○	○	○	○	○	○	○
Choose tab water instead of bottled water	○	○	○	○	○	○	○	○
Choose to drink water instead of sugar-sweetened beverages	○	○	○	○	○	○	○	○

	Target group cannot do this	Very unlikely	Unlikely	Somewhat likely	Likely	Very likely	Target group already does this	Not applicable/I don't know
Choose to drink other unsweetened beverages (e.g., tea) instead of sugar-sweetened beverages	○	○	○	○	○	○	○	○

	Target group cannot do this	Very unlikely	Unlikely	Somewhat likely	Likely	Very likely	Target group already does this	Not applicable/I don't know
Choose organically produced beverages (e.g., tea, coffee, juice)	○	○	○	○	○	○	○	○
Limit alcoholic beverage consumption to a max. of up to 2 glasses/per day for men and up to 1 glass/per day for women	○	○	○	○	○	○	○	○
Do not drink alcohol at an age under 18 years	○	○	○	○	○	○	○	○
Do not drink alcohol if you are pregnant or breast feeding	○	○	○	○	○	○	○	○

How likely is it that your target group would adopt the following **eating behaviours** in the following setting: **${q://QID7/ChoiceTextEntryValue/2}**

	Target group cannot do this	Very unlikely	Unlikely	Somewhat likely	Likely	Very likely	Target group already does this	Not applicable/I don't know
Eat 3 main meals every day	○	○	○	○	○	○	○	○
Eat more frequent and smaller meals (e.g. have a snack at mid-morning and mid-afternoon)	○	○	○	○	○	○	○	○
Eat breakfast	○	○	○	○	○	○	○	○
Accept a variety of foods	○	○	○	○	○	○	○	○
Encourage the acceptance of a variety of foods	○	○	○	○	○	○	○	○
Eat together as frequently as possible	○	○	○	○	○	○	○	○

	Target group cannot do this	Very unlikely	Unlikely	Somewhat likely	Likely	Very likely	Target group already does this	Not applicable/I don't know
Feed breast milk according to the baby's needs (if possible)	○	○	○	○	○	○	○	○
Eat mindfully and allow plenty of time for eating	○	○	○	○	○	○	○	○

How likely is it that your target group would adopt the following behaviours related to **nutritional needs** in the following setting: **${q://QID7/ChoiceTextEntryValue/2}**

	Target group cannot do this	Very unlikely	Unlikely	Somewhat likely	Likely	Very likely	Target group already does this	Not applicable/I don't know
Know your energy (caloric) needs and eat accordingly (don't over-/under-eat)	○	○	○	○	○	○	○	○
Inform yourself about your nutritional needs (e.g. macro and micro nutrients) with reliable resources	○	○	○	○	○	○	○	○
Ensure an adequate vitamin-D intake through sun exposure or supplementation (20 µg/d for an adult)	○	○	○	○	○	○	○	○
Ensure an adequate folic acid intake through supplementation (400 µg/d) if you are of childbearing age	○	○	○	○	○	○	○	○

Setting 2 Feasibility

How likely is it that relevant stakeholders would support your target group with adopting the following behaviours related to **legumes** in the following setting: **${q://QID7/ChoiceTextEntryValue/2}**

	Stakeholders can't support this	Very unlikely	Unlikely	Somewhat likely	Likely	Very likely	Stakeholders already support this	Not applicable/Don't know
Eat 3 servings of legumes per week (1 serving for an adult diet: 70 g raw / 125 g cooked)	○	○	○	○	○	○	○	○

	Stakeholders can't support this	Very unlikely	Unlikely	Somewhat likely	Likely	Very likely	Stakeholders already support this	Not applicable/Don't know
Choose a variety of legumes	○	○	○	○	○	○	○	○
Choose organically produced legumes	○	○	○	○	○	○	○	○
Choose regional legumes	○	○	○	○	○	○	○	○

How likely is it that relevant stakeholders would support your target group with adopting the following behaviours related to **vegetables and fruits** in the following setting: **${q://QID7/ChoiceTextEntryValue/2}**

	Stakeholders can't support this	Very unlikely	Unlikely	Somewhat likely	Likely	Very likely	Stakeholders already support this	Not applicable/Don't know
Eat 5 servings of vegetables and fruits per day (1 serving for an adult diet: 125 g)	○	○	○	○	○	○	○	○
Eat more vegetables than fruits (3-4 portions of vegetables / 1-2 portions of fruit)	○	○	○	○	○	○	○	○
Choose a variety of vegetables and fruits	○	○	○	○	○	○	○	○
	Stakeholders can't support this	Very unlikely	Unlikely	Somewhat likely	Likely	Very likely	Stakeholders already support this	Not applicable/Don't know
Choose organically produced vegetables and fruits	○	○	○	○	○	○	○	○
Choose regional vegetables and fruits	○	○	○	○	○	○	○	○
Choose seasonal vegetables and fruits	○	○	○	○	○	○	○	○

How likely is it that relevant stakeholders would support your target group with adopting the following behaviours related to **nuts and seeds** in the following

setting: **${q://QID7/ChoiceTextEntryValue/2}**

	Stakeholders can't support this	Very unlikely	Unlikely	Somewhat likely	Likely	Very likely	Stakeholders already support this	Not applicable/Don't know
Eat a small handful of nuts and seeds (2-)3 times a week (1 serving for an adult diet: 25 g)	○	○	○	○	○	○	○	○
Choose a variety of nuts and seeds	○	○	○	○	○	○	○	○
Choose organically produced nuts and seeds	○	○	○	○	○	○	○	○
Choose regional nuts and seeds	○	○	○	○	○	○	○	○

How likely is it that relevant stakeholders would support your target group with adopting the following behaviours related to **grains** in the following setting: **${q://QID7/ChoiceTextEntryValue/2}**

	Stakeholders can't support this	Very unlikely	Unlikely	Somewhat likely	Likely	Very likely	Stakeholders already support this	Not applicable/Don't know
Eat 3-6 servings of grain-based foods per day (1 serving for an adult diet: e.g., 40-60 g bread / 60-80 g dried pasta or dried rice)	○	○	○	○	○	○	○	○
Choose a variety of grain-based foods (e.g., flour types, pasta, rice etc.)	○	○	○	○	○	○	○	○
Choose primarily whole grains	○	○	○	○	○	○	○	○

	Stakeholders can't support this	Very unlikely	Unlikely	Somewhat likely	Likely	Very likely	Stakeholders already support this	Not applicable/Don't know
Choose organically produced grains	○	○	○	○	○	○	○	○
Choose regional grains	○	○	○	○	○	○	○	○

How likely is it that relevant stakeholders would support your target group with adopting the following behaviours related to **meat** in the following setting: **${q://QID7/ChoiceTextEntryValue/2}**

	Stakeholders can't support this	Very unlikely	Unlikely	Somewhat likely	Likely	Very likely	Stakeholders already support this	Not applicable/Don't know
Eat 0-3 servings of meat per week (1 serving for an adult diet: 100-125 g)	○	○	○	○	○	○	○	○
Limit the consumption of processed meat (both red and white meat) or even avoid it	○	○	○	○	○	○	○	○
Limit the consumption of red meat or even avoid it	○	○	○	○	○	○	○	○
Limit the consumption of all meats or even avoid it	○	○	○	○	○	○	○	○
	Stakeholders can't support this	Very unlikely	Unlikely	Somewhat likely	Likely	Very likely	Stakeholders already support this	Not applicable/Don't know
Choose poultry instead of red/processed meat	○	○	○	○	○	○	○	○
Choose organically produced meat	○	○	○	○	○	○	○	○
Choose regional meat	○	○	○	○	○	○	○	○
Choose dairy products instead of meat as an alternative source of protein	○	○	○	○	○	○	○	○

	Stakeholders can't support this	Very unlikely	Unlikely	Somewhat likely	Likely	Very likely	Stakeholders already support this	Not applicable/Don' know
	Stakeholders can't support this	Very unlikely	Unlikely	Somewhat likely	Likely	Very likely	Stakeholders already support this	Not applicable/Don' know
Choose eggs instead of meat as an alternative source of protein	○	○	○	○	○	○	○	○
Choose fish instead of meat as an alternative source of protein	○	○	○	○	○	○	○	○
Choose plant-based alternatives (e.g., legumes, nuts) instead of meat as an alternative source of protein	○	○	○	○	○	○	○	○

How likely is it that relevant stakeholders would support your target group with adopting the following behaviours related to **fish** in the following setting: **${q://QID7/ChoiceTextEntryValue/2}**

	Stakeholders can't support this	Very unlikely	Unlikely	Somewhat likely	Likely	Very likely	Stakeholders already support this	Not applicable/Don't know
Eat (2-)3 servings of fish and seafood per week (1 serving for an adult diet: 125-150g)	○	○	○	○	○	○	○	○
Choose (small) fatty fish (e.g. salmon, sardines, anchovies)	○	○	○	○	○	○	○	○
Choose organically produced fish	○	○	○	○	○	○	○	○
Choose domestic fish	○	○	○	○	○	○	○	○

How likely is it that relevant stakeholders would support your target group with adopting the following behaviours related to **eggs** in the following

setting: **${q://QID7/ChoiceTextEntryValue/2}**

	Stakeholders can't support this	Very unlikely	Unlikely	Somewhat likely	Likely	Very likely	Stakeholders already support this	Not applicable/Don't know
Eat 2-4 servings of eggs per week (1 serving for an adult diet: 1 egg)	○	○	○	○	○	○	○	○
Choose organically produced eggs	○	○	○	○	○	○	○	○
Choose regional eggs	○	○	○	○	○	○	○	○

How likely is it that relevant stakeholders would support your target group with adopting the following behaviours related to **milk and dairy products** in the following setting: **${q://QID7/ChoiceTextEntryValue/2}**

	Stakeholders can't support this	Very unlikely	Unlikely	Somewhat likely	Likely	Very likely	Stakeholders already support this	Not applicable/Don't know
Consume milk and dairy products daily (for an adult diet e.g., 200 g milk/dairy product and 60 g cheese per day)	○	○	○	○	○	○	○	○
Choose low-fat milk and dairy products instead of full-fat milk and dairy products	○	○	○	○	○	○	○	○
Choose plant-based alternatives to milk and dairy products	○	○	○	○	○	○	○	○
	Stakeholders can't support this	Very unlikely	Unlikely	Somewhat likely	Likely	Very likely	Stakeholders already support this	Not applicable/Don't know

	Stakeholders can't support this	Very unlikely	Unlikely	Somewhat likely	Likely	Very likely	Stakeholders already support this	Not applicable/Don't know
Choose organically produced milk and dairy products	○	○	○	○	○	○	○	○
Choose regional milk and dairy products	○	○	○	○	○	○	○	○

How likely is it that relevant stakeholders would support your target group with adopting the following behaviours related to **fats, sugar, and salt** in the following setting: **${q://QID7/ChoiceTextEntryValue/2}**

	Stakeholders can't support this	Very unlikely	Unlikely	Somewhat likely	Likely	Very likely	Stakeholders already support this	Not applicable/Don't know
Consume 30-40 g of oils (for an adult diet) per day	○	○	○	○	○	○	○	○
Choose vegetable oils (e.g., olive oil, rapeseed oil)	○	○	○	○	○	○	○	○
Choose organically produced oils	○	○	○	○	○	○	○	○
Choose regional oils	○	○	○	○	○	○	○	○
	Stakeholders can't support this	Very unlikely	Unlikely	Somewhat likely	Likely	Very likely	Stakeholders already support this	Not applicable/Don't know
Limit the consumption of added sugars (e.g., from sweets) (max. 25 g for an adult diet per day)	○	○	○	○	○	○	○	○
Choose organically produced sugar	○	○	○	○	○	○	○	○
Eat a max. of 6 g salt (for an adult diet) per day	○	○	○	○	○	○	○	○
Choose iodized salt	○	○	○	○	○	○	○	○
	Stakeholders can't	Very unlikely	Unlikely	Somewhat likely	Likely	Very likely	Stakeholders already	Not applicable/Don't

	Stakeholders can't support this support this	Very unlikely	Unlikely	Somewhat likely	Likely	Very likely	Stakeholders already support this support this	Not applicable/Don't know know
Choose fluoridated salt	○	○	○	○	○	○	○	○
For young children (babies), eat no added salt in meals	○	○	○	○	○	○	○	○
Add no salt to the baby meal	○	○	○	○	○	○	○	○
Limit the consumption of processed food products high in salt, sugars, and fats (e.g., fast food, salty snacks, biscuits, bars)	○	○	○	○	○	○	○	○

How likely is it that relevant stakeholders would support your target group with adopting the following behaviours related to **beverages** in the following setting: **${q://QID7/ChoiceTextEntryValue/2}**

	Stakeholders can't support this	Very unlikely	Unlikely	Somewhat likely	Likely	Very likely	Stakeholders already support this	Not applicable/Don't know
Drink 1.5-2L water (for an adult diet) per day	○	○	○	○	○	○	○	○
Choose tab water instead of bottled water	○	○	○	○	○	○	○	○
Choose to drink water instead of sugar-sweetened beverages	○	○	○	○	○	○	○	○
Choose to drink other unsweetened beverages (e.g., tea) instead of sugar-sweetened beverages	○	○	○	○	○	○	○	○
	Stakeholders can't support this	Very unlikely	Unlikely	Somewhat likely	Likely	Very likely	Stakeholders already support this	Not applicable/Don't know

	Stakeholders can't support this	Very unlikely	Unlikely	Somewhat likely	Likely	Very likely	Stakeholders already support this	Not applicable/Don't know
Choose organically produced beverages (e.g., tea, coffee, juice)	○	○	○	○	○	○	○	○
Limit alcoholic beverage consumption to a max. of up to 2 glasses/per day for men and up to 1 glass/per day for women	○	○	○	○	○	○	○	○
Do not drink alcohol at an age under 18 years	○	○	○	○	○	○	○	○
Do not drink alcohol if you are pregnant or breast feeding	○	○	○	○	○	○	○	○

How likely is it that relevant stakeholders would support your target group with adopting the following **eating behaviours** in the following setting: **${q://QID7/ChoiceTextEntryValue/2}**

	Stakeholders can't support this	Very unlikely	Unlikely	Somewhat likely	Likely	Very likely	Stakeholders already support this	Not applicable/Don't know
Eat 3 main meals every day	○	○	○	○	○	○	○	○
Eat more frequent and smaller meals (e.g. have a snack at mid-morning and mid-afternoon)	○	○	○	○	○	○	○	○
Eat breakfast	○	○	○	○	○	○	○	○
Accept a variety of foods	○	○	○	○	○	○	○	○

	Stakeholders can't support this	Very unlikely	Unlikely	Somewhat likely	Likely	Very likely	Stakeholders already support this	Not applicable/Don't know
Encourage the acceptance of a variety of foods	○	○	○	○	○	○	○	○

	Stakeholders can't support this	Very unlikely	Unlikely	Somewhat likely	Likely	Very likely	Stakeholders already support this	Not applicable/Don't know
Eat together as frequently as possible	○	○	○	○	○	○	○	○
Feed breast milk according to the baby's needs (if possible)	○	○	○	○	○	○	○	○
Eat mindfully and allow plenty of time for eating	○	○	○	○	○	○	○	○

How likely is it that relevant stakeholders would support your target group with adopting the following behaviours related to **nutritional needs** in the following setting: **${q://QID7/ChoiceTextEntryValue/2}**

	Stakeholders can't support this	Very unlikely	Unlikely	Somewhat likely	Likely	Very likely	Stakeholders already support this	Not applicable/Dc know
Know your energy (caloric) needs and eat accordingly (don't over-/under-eat)	○	○	○	○	○	○	○	○
Inform yourself about your nutritional needs (e.g. macro and micro nutrients) with reliable resources	○	○	○	○	○	○	○	○
Ensure an adequate vitamin-D intake through sun exposure or supplementation (20 µg/d for an adult)	○	○	○	○	○	○	○	○
Ensure an adequate folic acid intake through supplementation (400 µg/d) if you are of childbearing age	○	○	○	○	○	○	○	○

Setting 3 Plasticity

We will now ask you to rate all behaviours in the third setting you indicated:

${q://QID7/ChoiceTextEntryValue/3}

How likely is it that your target group would adopt the following behaviours related to **legumes** in the following setting: **${q://QID7/ChoiceTextEntryValue/3}**

	Target group cannot do this	Very unlikely	Unlikely	Somewhat likely	Likely	Very likely	Target group already does this	Not applicable/I don't know
Eat 3 servings of legumes per week (1 serving for an adult diet: 70 g raw / 125 g cooked)	○	○	○	○	○	○	○	○
Choose a variety of legumes	○	○	○	○	○	○	○	○
Choose organically produced legumes	○	○	○	○	○	○	○	○
Choose regional legumes	○	○	○	○	○	○	○	○

How likely is it that your target group would adopt the following behaviours related to **vegetables and fruits** in the following setting: **${q://QID7/ChoiceTextEntryValue/3}**

	Target group cannot do this	Very unlikely	Unlikely	Somewhat likely	Likely	Very likely	Target group already does this	Not applicable/I don't know
Eat 5 servings of vegetables and fruits per day (1 serving for an adult diet: 125 g)	○	○	○	○	○	○	○	○
Eat more vegetables than fruits (3-4 portions of vegetables / 1-2 portions of fruit)	○	○	○	○	○	○	○	○
Choose a variety of vegetables and fruits	○	○	○	○	○	○	○	○

	Target group cannot do this	Very unlikely	Unlikely	Somewhat likely	Likely	Very likely	Target group already does this	Not applicable/I don't know
Choose organically produced vegetables and fruits	○	○	○	○	○	○	○	○
Choose regional vegetables and fruits	○	○	○	○	○	○	○	○
Choose seasonal vegetables and fruits	○	○	○	○	○	○	○	○

How likely is it that your target group would adopt the following behaviours related to **nuts and seeds** in the following setting: **${q://QID7/ChoiceTextEntryValue/3}**

	Target group cannot do this	Very unlikely	Unlikely	Somewhat likely	Likely	Very likely	Target group already does this	Not applicable/I don't know
Eat a small handful of nuts and seeds (2-)3 times a week (1 serving for an adult diet: 25 g)	○	○	○	○	○	○	○	○
Choose a variety of nuts and seeds	○	○	○	○	○	○	○	○
Choose organically produced nuts and seeds	○	○	○	○	○	○	○	○
Choose regional nuts and seeds	○	○	○	○	○	○	○	○

How likely is it that your target group would adopt the following behaviours related to **grains** in the following setting: **${q://QID7/ChoiceTextEntryValue/3}**

	Target group cannot do this	Very unlikely	Unlikely	Somewhat likely	Likely	Very likely	Target group already does this	Not applicable/I don't know
Eat 3-6 servings of grain-based foods per day (1 serving for an adult diet: e.g., 40-60 g bread / 60-80 g dried pasta or dried rice)	○	○	○	○	○	○	○	○
Choose a variety of grain-based foods (e.g., flour types, pasta, rice etc.)	○	○	○	○	○	○	○	○
Choose primarily whole grains	○	○	○	○	○	○	○	○
Choose organically produced grains	○	○	○	○	○	○	○	○
Choose regional grains	○	○	○	○	○	○	○	○

How likely is it that your target group would adopt the following behaviours related to **meat** in the following setting: **${q://QID7/ChoiceTextEntryValue/3}**

	Target group cannot do this	Very unlikely	Unlikely	Somewhat likely	Likely	Very likely	Target group already does this	Not applicable/I don't know
Eat 0-3 servings of meat per week (1 serving for an adult diet: 100-125 g)	○	○	○	○	○	○	○	○

	Target group cannot do this	Very unlikely	Unlikely	Somewhat likely	Likely	Very likely	Target group already does this	Not applicable/I don't know
Limit the consumption of processed meat (both red and white meat) or even avoid it								
Limit the consumption of red meat or even avoid it								
Limit the consumption of all meats or even avoid it								

	Target group cannot do this	Very unlikely	Unlikely	Somewhat likely	Likely	Very likely	Target group already does this	Not applicable/I don't know
Choose poultry instead of red/processed meat								
Choose organically produced meat								
Choose regional meat								
Choose dairy products instead of meat as an alternative source of protein								

	Target group cannot do this	Very unlikely	Unlikely	Somewhat likely	Likely	Very likely	Target group already does this	Not applicable/I don't know
Choose eggs instead of meat as an alternative source of protein								
Choose fish instead of meat as an alternative source of protein								
Choose plant-based alternatives (e.g., legumes, nuts) instead of meat as an alternative source of protein								

How likely is it that your target group would adopt the following behaviours related to **fish** in the following setting: **${q://QID7/ChoiceTextEntryValue/3}**

	Target group cannot do this	Very unlikely	Unlikely	Somewhat likely	Likely	Very likely	Target group already does this	Not applicable/I don't know
Eat (2-)3 servings of fish and seafood per week (1 serving for an adult diet: 125-150g)	○	○	○	○	○	○	○	○
Choose (small) fatty fish (e.g. salmon, sardines, anchovies)	○	○	○	○	○	○	○	○
Choose organically produced fish	○	○	○	○	○	○	○	○
Choose domestic fish	○	○	○	○	○	○	○	○

How likely is it that your target group would adopt the following behaviours related to **eggs** in the following setting: **${q://QID7/ChoiceTextEntryValue/3}**

	Target group cannot do this	Very unlikely	Unlikely	Somewhat likely	Likely	Very likely	Target group already does this	Not applicable/I don't know
Eat 2-4 servings of eggs per week (1 serving for an adult diet: 1 egg)	○	○	○	○	○	○	○	○
Choose organically produced eggs	○	○	○	○	○	○	○	○
Choose regional eggs	○	○	○	○	○	○	○	○

How likely is it that your target group would adopt the following behaviours related to **milk and dairy products** in the following setting: **${q://QID7/ChoiceTextEntryValue/3}**

	Target group cannot do this	Very unlikely	Unlikely	Somewhat likely	Likely	Very likely	Target group already does this	Not applicable/I don't know
Consume milk and dairy products daily (for an adult diet e.g., 200 g milk/dairy product and 60 g cheese per day)	○	○	○	○	○	○	○	○
Choose low-fat milk and dairy products instead of full-fat milk and dairy products	○	○	○	○	○	○	○	○
Choose plant-based alternatives to milk and dairy products	○	○	○	○	○	○	○	○
Choose organically produced milk and dairy products	○	○	○	○	○	○	○	○

	Target group cannot do this	Very unlikely	Unlikely	Somewhat likely	Likely	Very likely	Target group already does this	Not applicable/I don't know
Choose regional milk and dairy products	○	○	○	○	○	○	○	○

How likely is it that your target group would adopt the following behaviours related to **fats, sugar, and salt** in the following setting: **${q://QID7/ChoiceTextEntryValue/3}**

	Target group cannot do this	Very unlikely	Unlikely	Somewhat likely	Likely	Very likely	Target group already does this	Not applicable/I don't know
Consume 30-40 g of oils (for an adult diet) per day	○	○	○	○	○	○	○	○
Choose vegetable oils (e.g., olive oil, rapeseed oil)	○	○	○	○	○	○	○	○
Choose organically produced oils	○	○	○	○	○	○	○	○
Choose regional oils	○	○	○	○	○	○	○	○

	Target group cannot do this	Very unlikely	Unlikely	Somewhat likely	Likely	Very likely	Target group already does this	Not applicable/I don't know
Limit the consumption of added sugars (e.g., from sweets) (max. 25 g for an adult diet per day)	○	○	○	○	○	○	○	○
Choose organically produced sugar	○	○	○	○	○	○	○	○
Eat a max. of 6 g salt (for an adult diet) per day	○	○	○	○	○	○	○	○
Choose iodized salt	○	○	○	○	○	○	○	○

	Target group cannot do this	Very unlikely	Unlikely	Somewhat likely	Likely	Very likely	Target group already does this	Not applicable/I don't know
Choose fluoridated salt	○	○	○	○	○	○	○	○
For young children (babies), eat no added salt in meals	○	○	○	○	○	○	○	○
Add no salt to the baby meal	○	○	○	○	○	○	○	○

	Target group cannot do this	Very unlikely	Unlikely	Somewhat likely	Likely	Very likely	Target group already does this	Not applicable/I don't know
Limit the consumption of processed food products high in salt, sugars, and fats (e.g., fast food, salty snacks, biscuits, bars)	○	○	○	○	○	○	○	○

How likely is it that your target group would adopt the following behaviours related to **beverages** in the following setting: **${q://QID7/ChoiceTextEntryValue/3}**

	Target group cannot do this	Very unlikely	Unlikely	Somewhat likely	Likely	Very likely	Target group already does this	Not applicable/I don't know
Drink 1.5-2L water (for an adult diet) per day	○	○	○	○	○	○	○	○
Choose tab water instead of bottled water	○	○	○	○	○	○	○	○
Choose to drink water instead of sugar-sweetened beverages	○	○	○	○	○	○	○	○
Choose to drink other unsweetened beverages (e.g., tea) instead of sugar-sweetened beverages	○	○	○	○	○	○	○	○

	Target group cannot do this	Very unlikely	Unlikely	Somewhat likely	Likely	Very likely	Target group already does this	Not applicable/I don't know
Choose organically produced beverages (e.g., tea, coffee, juice)	○	○	○	○	○	○	○	○
Limit alcoholic beverage consumption to a max. of up to 2 glasses/per day for men and up to 1 glass/per day for women	○	○	○	○	○	○	○	○
Do not drink alcohol at an age under 18 years	○	○	○	○	○	○	○	○
Do not drink alcohol if you are pregnant or breast feeding	○	○	○	○	○	○	○	○

How likely is it that your target group would adopt the following **eating behaviours** in the following setting: **${q://QID7/ChoiceTextEntryValue/3}**

	Target group cannot do this	Very unlikely	Unlikely	Somewhat likely	Likely	Very likely	Target group already does this	Not applicable/I don't know
Eat 3 main meals every day	○	○	○	○	○	○	○	○
Eat more frequent and smaller meals (e.g. have a snack at mid-morning and mid-afternoon)	○	○	○	○	○	○	○	○
Eat breakfast	○	○	○	○	○	○	○	○
Accept a variety of foods	○	○	○	○	○	○	○	○
Encourage the acceptance of a variety of foods	○	○	○	○	○	○	○	○
Eat together as frequently as possible	○	○	○	○	○	○	○	○
Feed breast milk according to the baby's needs (if possible)	○	○	○	○	○	○	○	○
Eat mindfully and allow plenty of time for eating	○	○	○	○	○	○	○	○

How likely is it that your target group would adopt the following behaviours related to **nutritional needs** in the following setting: **${q://QID7/ChoiceTextEntryValue/3}**

	Target group cannot do this	Very unlikely	Unlikely	Somewhat likely	Likely	Very likely	Target group already does this	Not applicable/I don't know
Know your energy (caloric) needs and eat accordingly (don't over-/under-eat)	○	○	○	○	○	○	○	○
Inform yourself about your nutritional needs (e.g. macro and micro nutrients) with reliable resources	○	○	○	○	○	○	○	○
Ensure an adequate vitamin-D intake through sun exposure or supplementation (20 µg/d for an adult)	○	○	○	○	○	○	○	○
Ensure an adequate folic acid intake through supplementation (400 µg/d) if you are of childbearing age	○	○	○	○	○	○	○	○

Setting 3 Feasibility

How likely is it that relevant stakeholders would support your target group with adopting the following behaviours related to **legumes** in the following setting: **${q://QID7/ChoiceTextEntryValue/3}**

	Stakeholders can't support this	Very unlikely	Unlikely	Somewhat likely	Likely	Very likely	Stakeholders already support this	Not applicable/Don't know
Eat 3 servings of legumes per week (1 serving for an adult diet: 70 g raw / 125 g cooked)	○	○	○	○	○	○	○	○
Choose a variety of legumes	○	○	○	○	○	○	○	○
Choose organically produced legumes	○	○	○	○	○	○	○	○
Choose regional legumes	○	○	○	○	○	○	○	○

How likely is it that relevant stakeholders would support your target group with adopting the following behaviours related to **vegetables and fruits** in the following setting: **${q://QID7/ChoiceTextEntryValue/3}**

	Stakeholders can't support this	Very unlikely	Unlikely	Somewhat likely	Likely	Very likely	Stakeholders already support this	Not applicable/Don't know
Eat 5 servings of vegetables and fruits per day (1 serving for an adult diet: 125 g)	○	○	○	○	○	○	○	○
Eat more vegetables than fruits (3-4 portions of vegetables / 1-2 portions of fruit)	○	○	○	○	○	○	○	○
Choose a variety of vegetables and fruits	○	○	○	○	○	○	○	○
	Stakeholders can't support this	Very unlikely	Unlikely	Somewhat likely	Likely	Very likely	Stakeholders already support this	Not applicable/Don't know

	Stakeholders can't support this	Very unlikely	Unlikely	Somewhat likely	Likely	Very likely	Stakeholders already support this	Not applicable/Don't know
Choose organically produced vegetables and fruits	○	○	○	○	○	○	○	○
Choose regional vegetables and fruits	○	○	○	○	○	○	○	○
Choose seasonal vegetables and fruits	○	○	○	○	○	○	○	○

How likely is it that relevant stakeholders would support your target group with adopting the following behaviours related to **nuts and seeds** in the following setting: **${q://QID7/ChoiceTextEntryValue/3}**

	Stakeholders can't support this	Very unlikely	Unlikely	Somewhat likely	Likely	Very likely	Stakeholders already support this	Not applicable/Don't know
Eat a small handful of nuts and seeds (2-)3 times a week (1 serving for an adult diet: 25 g)	○	○	○	○	○	○	○	○
Choose a variety of nuts and seeds	○	○	○	○	○	○	○	○
Choose organically produced nuts and seeds	○	○	○	○	○	○	○	○
Choose regional nuts and seeds	○	○	○	○	○	○	○	○

How likely is it that relevant stakeholders would support your target group with adopting the following behaviours related to **grains** in the following setting: **${q://QID7/ChoiceTextEntryValue/3}**

	Stakeholders can't support this	Very unlikely	Unlikely	Somewhat likely	Likely	Very likely	Stakeholders already support this	Not applicable/Don't know
Eat 3-6 servings of grain-based foods per day (1 serving for an adult diet: e.g., 40-60 g bread / 60-80 g dried pasta or dried rice)	○	○	○	○	○	○	○	○
Choose a variety of grain-based foods (e.g., flour types, pasta, rice etc.)	○	○	○	○	○	○	○	○
Choose primarily whole grains	○	○	○	○	○	○	○	○
Choose organically produced grains	○	○	○	○	○	○	○	○
Choose regional grains	○	○	○	○	○	○	○	○

How likely is it that relevant stakeholders would support your target group with adopting the following behaviours related to **meat** in the following setting: **${q://QID7/ChoiceTextEntryValue/3}**

	Stakeholders can't support this	Very unlikely	Unlikely	Somewhat likely	Likely	Very likely	Stakeholders already support this	Not applicable/Don't know
Eat 0-3 servings of meat per week (1 serving for an adult diet: 100-125 g)	○	○	○	○	○	○	○	○
Limit the consumption of processed meat (both red and white meat) or even avoid it	○	○	○	○	○	○	○	○
Limit the consumption of red meat or even avoid it	○	○	○	○	○	○	○	○

	Stakeholders can't support this	Very unlikely	Unlikely	Somewhat likely	Likely	Very likely	Stakeholders already support this	Not applicable/Don't know
Limit the consumption of all meats or even avoid it								

	Stakeholders can't support this	Very unlikely	Unlikely	Somewhat likely	Likely	Very likely	Stakeholders already support this	Not applicable/Don't know
Choose poultry instead of red/processed meat								
Choose organically produced meat								
Choose regional meat								
Choose dairy products instead of meat as an alternative source of protein								

	Stakeholders can't support this	Very unlikely	Unlikely	Somewhat likely	Likely	Very likely	Stakeholders already support this	Not applicable/Don't know
Choose eggs instead of meat as an alternative source of protein								
Choose fish instead of meat as an alternative source of protein								
Choose plant-based alternatives (e.g., legumes, nuts) instead of meat as an alternative source of protein								

How likely is it that relevant stakeholders would support your target group with adopting the following behaviours related to **fish** in the following setting: **${q://QID7/ChoiceTextEntryValue/3}**

	Stakeholders can't support this	Very unlikely	Unlikely	Somewhat likely	Likely	Very likely	Stakeholders already support this	Not applicable/Don't know
Eat (2-)3 servings of fish and seafood per week (1 serving for an adult diet: 125-150g)	○	○	○	○	○	○	○	○
Choose (small) fatty fish (e.g. salmon, sardines, anchovies)	○	○	○	○	○	○	○	○
Choose organically produced fish	○	○	○	○	○	○	○	○
Choose domestic fish	○	○	○	○	○	○	○	○

How likely is it that relevant stakeholders would support your target group with adopting the following behaviours related to **eggs** in the following setting: **${q://QID7/ChoiceTextEntryValue/3}**

	Stakeholders can't support this	Very unlikely	Unlikely	Somewhat likely	Likely	Very likely	Stakeholders already support this	Not applicable/Don't know
Eat 2-4 servings of eggs per week (1 serving for an adult diet: 1 egg)	○	○	○	○	○	○	○	○
Choose organically produced eggs	○	○	○	○	○	○	○	○
Choose regional eggs	○	○	○	○	○	○	○	○

How likely is it that relevant stakeholders would support your target group with adopting the following behaviours related to **milk and dairy products** in the following setting: **${q://QID7/ChoiceTextEntryValue/3}**

	Stakeholders can't support this	Very unlikely	Unlikely	Somewhat likely	Likely	Very likely	Stakeholders already support this	Not applicable/Don't know
Consume milk and dairy products daily (for an adult diet e.g., 200 g milk/dairy product and 60 g cheese per day)	○	○	○	○	○	○	○	○
Choose low-fat milk and dairy products instead of full-fat milk and dairy products	○	○	○	○	○	○	○	○
Choose plant-based alternatives to milk and dairy products	○	○	○	○	○	○	○	○
	Stakeholders can't support this	Very unlikely	Unlikely	Somewhat likely	Likely	Very likely	Stakeholders already support this	Not applicable/Don't know
Choose organically produced milk and dairy products	○	○	○	○	○	○	○	○
Choose regional milk and dairy products	○	○	○	○	○	○	○	○

How likely is it that relevant stakeholders would support your target group with adopting the following behaviours related to **fats, sugar, and salt** in the following setting: **${q://QID7/ChoiceTextEntryValue/3}**

	Stakeholders can't support this	Very unlikely	Unlikely	Somewhat likely	Likely	Very likely	Stakeholders already support this	Not applicable/Don't know
Consume 30-40 g of oils (for an adult diet) per day	○	○	○	○	○	○	○	○
Choose vegetable oils (e.g., olive oil, rapeseed oil)	○	○	○	○	○	○	○	○

	Stakeholders can't support this	Very unlikely	Unlikely	Somewhat likely	Likely	Very likely	Stakeholders already support this	Not applicable/Don't know
Choose organically produced oils	○	○	○	○	○	○	○	○
Choose regional oils	○	○	○	○	○	○	○	○
	Stakeholders can't support this	Very unlikely	Unlikely	Somewhat likely	Likely	Very likely	Stakeholders already support this	Not applicable/Don't know
Limit the consumption of added sugars (e.g., from sweets) (max. 25 g for an adult diet per day)	○	○	○	○	○	○	○	○
Choose organically produced sugar	○	○	○	○	○	○	○	○
Eat a max. of 6 g salt (for an adult diet) per day	○	○	○	○	○	○	○	○
Choose iodized salt	○	○	○	○	○	○	○	○
	Stakeholders can't support this	Very unlikely	Unlikely	Somewhat likely	Likely	Very likely	Stakeholders already support this	Not applicable/Don't know
Choose fluoridated salt	○	○	○	○	○	○	○	○
For young children (babies), eat no added salt in meals	○	○	○	○	○	○	○	○
Add no salt to the baby meal	○	○	○	○	○	○	○	○
Limit the consumption of processed food products high in salt, sugars, and fats (e.g., fast food, salty snacks, biscuits, bars)	○	○	○	○	○	○	○	○

How likely is it that relevant stakeholders would support your target group with adopting the following behaviours related to **beverages** in the following setting: **${q://QID7/ChoiceTextEntryValue/3}**

	Stakeholders can't support this	Very unlikely	Unlikely	Somewhat likely	Likely	Very likely	Stakeholders already support this	Not applicable/Don't know
Drink 1.5-2L water (for an adult diet) per day	○	○	○	○	○	○	○	○
Choose tab water instead of bottled water	○	○	○	○	○	○	○	○
Choose to drink water instead of sugar-sweetened beverages	○	○	○	○	○	○	○	○
Choose to drink other unsweetened beverages (e.g., tea) instead of sugar-sweetened beverages	○	○	○	○	○	○	○	○
	Stakeholders can't support this	Very unlikely	Unlikely	Somewhat likely	Likely	Very likely	Stakeholders already support this	Not applicable/Don't know
Choose organically produced beverages (e.g., tea, coffee, juice)	○	○	○	○	○	○	○	○
Limit alcoholic beverage consumption to a max. of up to 2 glasses/per day for men and up to 1 glass/per day for women	○	○	○	○	○	○	○	○
Do not drink alcohol at an age under 18 years	○	○	○	○	○	○	○	○
Do not drink alcohol if you are pregnant or breast feeding	○	○	○	○	○	○	○	○

How likely is it that relevant stakeholders would support your target group with adopting the following **eating behaviours** in the following setting: **${q://QID7/ChoiceTextEntryValue/3}**

	Stakeholders can't support this	Very unlikely	Unlikely	Somewhat likely	Likely	Very likely	Stakeholders already support this	Not applicable/Don't know
Eat 3 main meals every day	○	○	○	○	○	○	○	○
Eat more frequent and smaller meals (e.g. have a snack at mid-morning and mid-afternoon)	○	○	○	○	○	○	○	○
Eat breakfast	○	○	○	○	○	○	○	○
Accept a variety of foods	○	○	○	○	○	○	○	○

	Stakeholders can't support this	Very unlikely	Unlikely	Somewhat likely	Likely	Very likely	Stakeholders already support this	Not applicable/Don't know
Encourage the acceptance of a variety of foods	○	○	○	○	○	○	○	○
Eat together as frequently as possible	○	○	○	○	○	○	○	○
Feed breast milk according to the baby's needs (if possible)	○	○	○	○	○	○	○	○
Eat mindfully and allow plenty of time for eating	○	○	○	○	○	○	○	○

How likely is it that relevant stakeholders would support your target group with adopting the following behaviours related to **nutritional needs** in the following setting: **${q://QID7/ChoiceTextEntryValue/3}**

	Stakeholders can't support this	Very unlikely	Unlikely	Somewhat likely	Likely	Very likely	Stakeholders already support this	Not applicable/Dc know
Know your energy (caloric) needs and eat accordingly (don't over-/under-eat)	○	○	○	○	○	○	○	○

	Stakeholders can't support this	Very unlikely	Unlikely	Somewhat likely	Likely	Very likely	Stakeholders already support this	Not applicable/Dc know
Inform yourself about your nutritional needs (e.g. macro and micro nutrients) with reliable resources	○	○	○	○	○	○	○	○
Ensure an adequate vitamin-D intake through sun exposure or supplementation (20 µg/d for an adult)	○	○	○	○	○	○	○	○
Ensure an adequate folic acid intake through supplementation (400 µg/d) if you are of childbearing age	○	○	○	○	○	○	○	○

Other behaviours

Are there any further behaviours you would like to rate? (e.g. limit the consumption of fish, eat tofu X times a week)

Please name up to three behaviours.
In the following you will again rate behavioural plasticity and feasibility for each behaviour in each setting separately.

If you do not have any further behaviours, please leave the following text fields empty.

1. Further behaviour ☐
2. Further behaviour ☐
3. Further behaviour ☐

Other behaviours: Setting 1 Plasticity

How likely is it that your target group would adopt the following **eating behaviours** in the following setting: **${q://QID7/ChoiceTextEntryValue/1}**

	Target group cannot do this	Very unlikely	Unlikely	Somewhat likely	Likely	Very likely	Target group already does this	N applic don't
${q://QID16/ChoiceTextEntryValue/1}	○	○	○	○	○	○	○	○
${q://QID16/ChoiceTextEntryValue/2}	○	○	○	○	○	○	○	○
${q://QID16/ChoiceTextEntryValue/3}	○	○	○	○	○	○	○	○

Other behaviours: Setting 1 Feasibility

How likely is it that relevant stakeholders would support your target group with adopting the following **eating behaviours** in the following setting: **${q://QID7/ChoiceTextEntryValue/1}**

	Stakeholders can't support this	Very unlikely	Unlikely	Somewhat likely	Likely	Very likely	Stakeho alrea suppor
${q://QID16/ChoiceTextEntryValue/1}	○	○	○	○	○	○	○
${q://QID16/ChoiceTextEntryValue/2}	○	○	○	○	○	○	○
${q://QID16/ChoiceTextEntryValue/3}	○	○	○	○	○	○	○

Other behaviours: Setting 2 Plasticity

How likely is it that your target group would adopt the following **eating behaviours** in the following setting: **${q://QID7/ChoiceTextEntryValue/2}**

	Target group cannot do this	Very unlikely	Unlikely	Somewhat likely	Likely	Very likely	Target group already does this	N applic don't
${q://QID16/ChoiceTextEntryValue/1}	○	○	○	○	○	○	○	○
${q://QID16/ChoiceTextEntryValue/2}	○	○	○	○	○	○	○	○
${q://QID16/ChoiceTextEntryValue/3}	○	○	○	○	○	○	○	○

Other behaviours: Setting 2 Feasibility

How likely is it that relevant stakeholders would support your target group with adopting the following **eating behaviours** in the following setting: **${q://QID7/ChoiceTextEntryValue/2}**

	Stakeholders can't support this	Very unlikely	Unlikely	Somewhat likely	Likely	Very likely	Stakeho alrea suppor
${q://QID16/ChoiceTextEntryValue/1}	○	○	○	○	○	○	○
${q://QID16/ChoiceTextEntryValue/2}	○	○	○	○	○	○	○
${q://QID16/ChoiceTextEntryValue/3}	○	○	○	○	○	○	○

Other behaviours: Setting 3 Plasticity

How likely is it that your target group would adopt the following **eating behaviours** in the following setting: **${q://QID7/ChoiceTextEntryValue/3}**

	Target group cannot do this	Very unlikely	Unlikely	Somewhat likely	Likely	Very likely	Target group already does this	N applic don't
${q://QID16/ChoiceTextEntryValue/1}	○	○	○	○	○	○	○	○
${q://QID16/ChoiceTextEntryValue/2}	○	○	○	○	○	○	○	○
${q://QID16/ChoiceTextEntryValue/3}	○	○	○	○	○	○	○	○

Other behaviours: Setting 3 Feasibility

How likely is it that relevant stakeholders would support your target group with adopting the following **eating behaviours** in the following setting: **${q://QID7/ChoiceTextEntryValue/3}**

	Stakeholders can't support this	Very unlikely	Unlikely	Somewhat likely	Likely	Very likely	Stakeho alrea suppor
${q://QID16/ChoiceTextEntryValue/1}	○	○	○	○	○	○	○
${q://QID16/ChoiceTextEntryValue/2}	○	○	○	○	○	○	○
${q://QID16/ChoiceTextEntryValue/3}	○	○	○	○	○	○	○

Comments and end survey

Please write here if you have any further comments, or bring them with you to the interview!

Powered by Qualtrics
