# Supplementary material for: Identifying healthy and sustainable high-impact eating behaviour in French children aged 6–15 years: a combined multidisciplinary and living lab participatory approach
Source: J Nutr Sci. 2026 May 26;15:e38. doi: 10.1017/jns.2026.10105 (PMC13227139; doi:10.1017/jns.2026.10105)
Supplement: Fardet et al. supplementary material 7 — Fardet et al. supplementary material [file S2048679026101050sup007.pdf]

Plan’Eat T2.1 Expert interviews

1) **Questions about you and your target group**
   a) What is your role in the organization?
   b) Who is your target group? How do you work with the target groups/how do you have access to them?

2) **Questions about the survey**
   a) What was your experience filling in the survey?
      i) What does the survey capture well?
      ii) What does it miss?
   b) Do the behaviours we listed need to be modified for your target group? E.g. serving sizes? Would you be able to provide exact serving sizes for your target group?
   c) Which settings did you chose and why?
   d) Which stakeholder did you think about?

3) **High potential behaviours in the near future, i.e., high likelihood that target group will adopt the behaviour and/or high support**
   a) Which behaviours have a very high potential? Why?
      i) Likelihood of adaptation by target group high
      ii) Support from important stakeholders high
   b) Do they need more specific definition/can we work with the behaviour as written in the survey?
   → ASK THIS OPEN FIRST, then go through survey answers

4) **Low potential behaviours in the near future, i.e., low likelihood that target group will adopt the behaviour and/or low support**
   a) Which behaviours have a very low potential? Why?
      i) Likelihood of adaptation by target group low
      ii) Support from important stakeholders low
   b) Shall we exclude these behaviours from our further work?
   → ASK THIS OPEN FIRST, then go through survey answers

<u>**I don‘t know /not applicable**</u> → ask in interview why
<u>**Target group already does it**</u> → Do we have data/evidence?
<u>**Stakeholders already support this**</u> → how?

5) **Rating difficulties & knowledge gaps**
   a) Which behaviours were difficult to rate?
   b) Where do we have gaps in the knowledge? How can we fill them/decide?

6) **Further behaviours**
   a) Are there further behaviours that are important to add/discuss/rate?

7) **Summary**
   a) What are the main current problem behaviours for your target group?
   b) Are these the ones we should work with?
   c) If you could pick 5 behaviours to work with for your target group, which ones would that be? Why? SHOW SLIDE

d) What are their main problems/challenges your target group faces in achieving those behaviours?

8) **Future research in Plan’Eat**

e) What survey methods are possible with your target group? (standard survey, diary survey)

f) What interventions are possible with your target group?

g) What surveys have you planned and when?
