# Supplementary material for: Identifying healthy and sustainable high-impact eating behaviour in French children aged 6–15 years: a combined multidisciplinary and living lab participatory approach
Source: J Nutr Sci. 2026 May 26;15:e38. doi: 10.1017/jns.2026.10105 (PMC13227139; doi:10.1017/jns.2026.10105)
Supplement: Fardet et al. supplementary material 8 — Fardet et al. supplementary material [file S2048679026101050sup008.pdf]

WORK SHEET

Workshop: 'Five high impact behaviours' (03.06.2023)

Welcome dear LL France to our workshop! We are happy to have you join us today. Our workshop is designed to provide you with an overview of our research so far and to give you an opportunity to make a final selection of high impact behaviours. Throughout the session, you will need the following work sheet and there will be time provided to answer the questions asked. We hope that you find this workshop to be informative, engaging, and enjoyable.

Workshop materials

On page 4 and forward you can find the results from the surveys for your LL France. Depicted are the 40 behaviours with the highest environmental and health potential. The graph shows your individual rating of these behaviours by setting with regard to *plasticity* and *feasibility*. Your settings are: school restaurants, middle to lower class families and upper class families.

- *Plasticity* refers to the following question: How likely is it that your target group would adopt this behaviour?
- *Feasibility* refers to: How likely is it that relevant stakeholder (e.g., teachers, parents, employers, canteen providers) would support your target group with adopting this behaviour?

<u>Reflection and decision: identifying behaviours and describing them in detail</u>

In the following, you will have the chance to make changes to the survey answers, choose the five high impact behaviours and describe them to us in more detail.

<u>*Task 1*</u>: Please have a look at the graph and see what is depicted. Do you want to change a rating? Does something come to mind as you see these results? Feel free to add comments as you like.

Based on your ratings in the survey and our interview discussion, we suggest the following **high impact behaviours for your LL**:

1. Choose to drink water instead of sugar-sweetened beverages
   (Target group cannot do this in school restaurant setting?)
2. Limit the consumption of processed food products high in salt, sugars, and fats
   (e.g., fast food, salty snacks, biscuits, bars)
3. Eat 0-3 servings of meat per week (1 serving for an adult diet: 100-125 g)
4. Eat 5 servings of vegetables and fruits per day (1 serving for an adult diet: 125 g)
5. Accept a variety of foods (Middle to lower class families?)

Task 2: Do you agree with our selection?

- Feel free to make changes and replace behaviours with others you see. However, please make sure to have at least two food groups represented in the final selection of behaviours (e.g., meat and fruit/vegetables).
- Please rank the behaviours you chose in order of importance, i.e., rank them reflecting your opinion which is the most important to address in your LL to least important.
- You can choose up to maximum 5 behaviours, but you don't have to. Feel free to delete some behaviours we suggested. Less than 5 behaviours are fine, too!

Please write below, in the order of importance, one behaviour after each number. Please make sure at least two food groups are represented.

1.

2.

3.

4.

5.

Task 3: Please explain the behaviours more in detail, so we understand them better before developing surveys and interventions for them. For each behaviour, please answer the following questions:

- How much a of a problem is this behaviour in your (sub-)target group? E.g., how often do they do this already?
- Which are the most important settings for this behaviour? Where does it occur, or in which settings can we perhaps change this behaviour the best?
- Who are important stakeholders in those settings that we need to take into account? How do they impact the target group and the behaviour? Do they enable it, or hinder it?

Please first name the behaviour you are currently describing. After, please write a few words each for occurrence, most important settings and most important stakeholders including their role. Please take your time to complete this task and provide as much detail as possible.

1. Behaviour:

a) (Sub-) target group for which this behaviour is most relevant:

b) Current situation: occurrence of the behaviour, why is it a problem etc.:

c) Most important settings:

d) Most important stakeholders and their role:

2. Behaviour:

a) (Sub-) target group for which this behaviour is most relevant:

b) Current situation: occurrence of the behaviour, why is it a problem etc.:

c) Most important settings:

d) Most important stakeholders and their role:

3. Behaviour:

a) (Sub-) target group for which this behaviour is most relevant:

b) Current situation: occurrence of the behaviour, why is it a problem etc.:

c) Most important settings:

d) Most important stakeholders and their role:

4. Behaviour:

a) (Sub-) target group for which this behaviour is most relevant:

b) Current situation: occurrence of the behaviour, why is it a problem etc.:

c) Most important settings:

d) Most important stakeholders and their role:

5. Behaviour:

a) (Sub-) target group for which this behaviour is most relevant:

b) Current situation: occurrence of the behaviour, why is it a problem etc.:

c) Most important settings:

d) Most important stakeholders and their role:

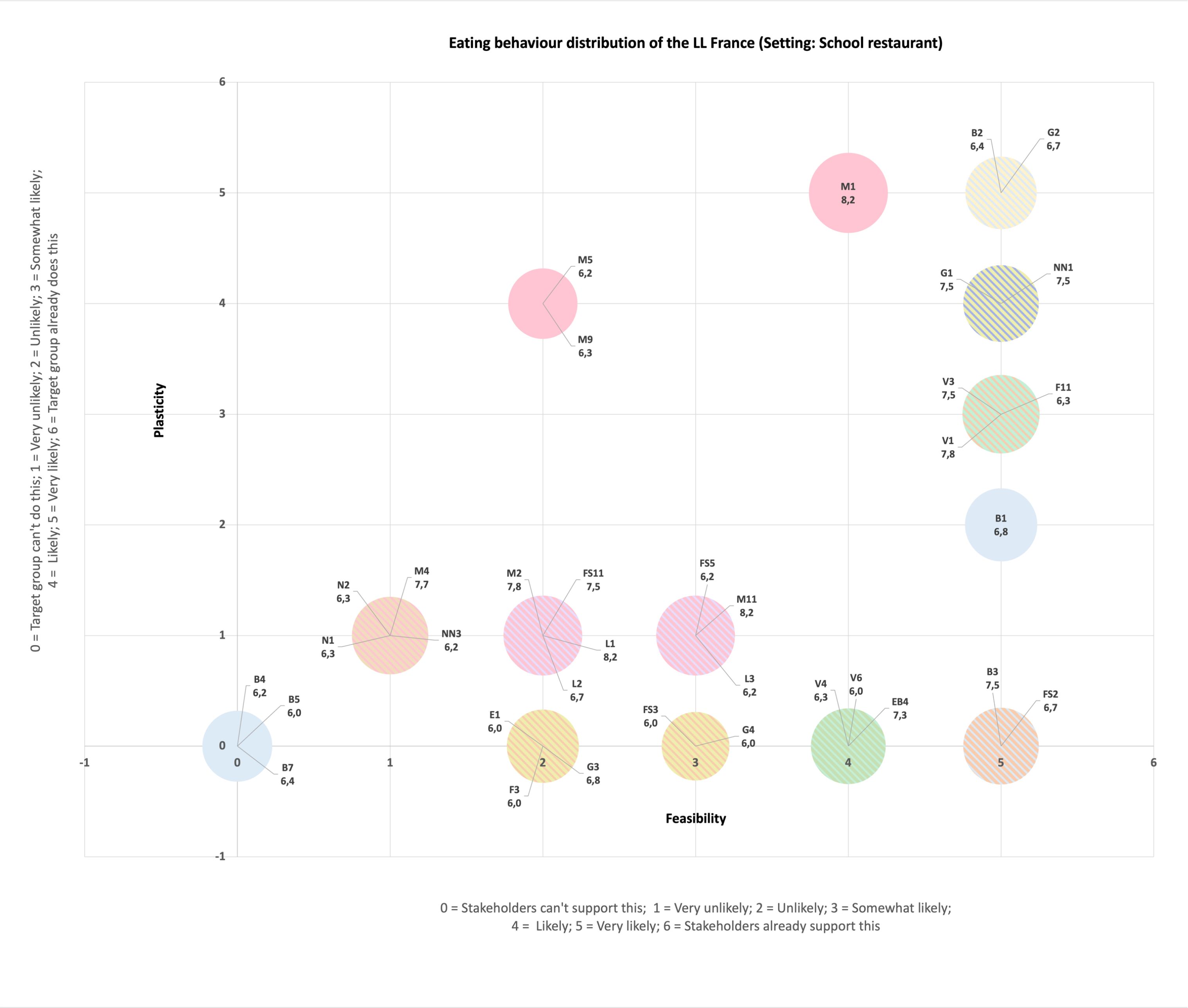

Eating behaviour distribution of the LL France (Setting: School restaurant)
0 = Target group can't do this; 1 = Very unlikely; 2 = Unlikely; 3 = Somewhat likely; 4 = Likely; 5 = Very likely; 6 = Target group already does this
Plasticity
Feasibility
0 = Stakeholders can't support this; 1 = Very unlikely; 2 = Unlikely; 3 = Somewhat likely; 4 = Likely; 5 = Very likely; 6 = Stakeholders already support this
M1 8,2
B2 6,4
G2 6,7
M5 6,2
M9 6,3
G1 7,5
NN1 7,5
V3 7,5
F11 6,3
V1 7,8
B1 6,8
N2 6,3
M4 7,7
N1 6,3
NN3 6,2
M2 7,8
FS11 7,5
L1 8,2
L2 6,7
FS5 6,2
M11 8,2
L3 6,2
B4 6,2
B5 6,0
B7 6,4
E1 6,0
G3 6,8
F3 6,0
FS3 6,0
G4 6,0
V4 6,3
V6 6,0
EB4 7,3
B3 7,5
FS2 6,7

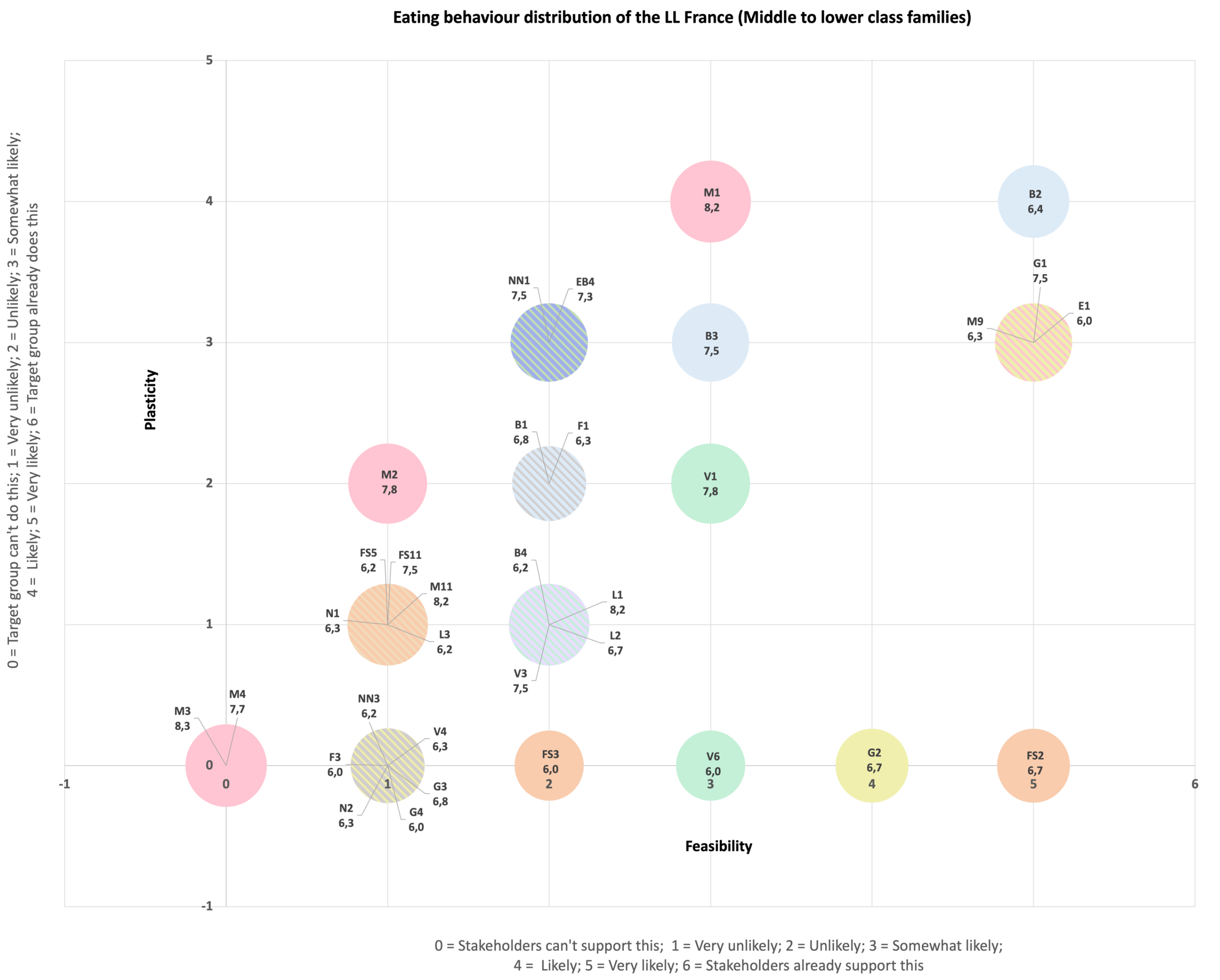
Eating behaviour distribution of the LL France (Middle to lower class families)
Plasticity
0 = Target group can't do this; 1 = Very unlikely; 2 = Unlikely; 3 = Somewhat likely; 4 = Likely; 5 = Very likely; 6 = Target group already does this
Feasibility
0 = Stakeholders can't support this; 1 = Very unlikely; 2 = Unlikely; 3 = Somewhat likely; 4 = Likely; 5 = Very likely; 6 = Stakeholders already support this
M1 8,2
B2 6,4
NN1 7,5
EB4 7,3
B3 7,5
G1 7,5
E1 6,0
M9 6,3
B1 6,8
F1 6,3
M2 7,8
V1 7,8
FS5 6,2
FS11 7,5
M11 8,2
N1 6,3
L3 6,2
B4 6,2
L1 8,2
L2 6,7
V3 7,5
M3 8,3
M4 7,7
NN3 6,2
V4 6,3
F3 6,0
G3 6,8
N2 6,3
G4 6,0
FS3 6,0
V6 6,0
G2 6,7
FS2 6,7

Eating behaviour distribution of the LL France (Upper class families)

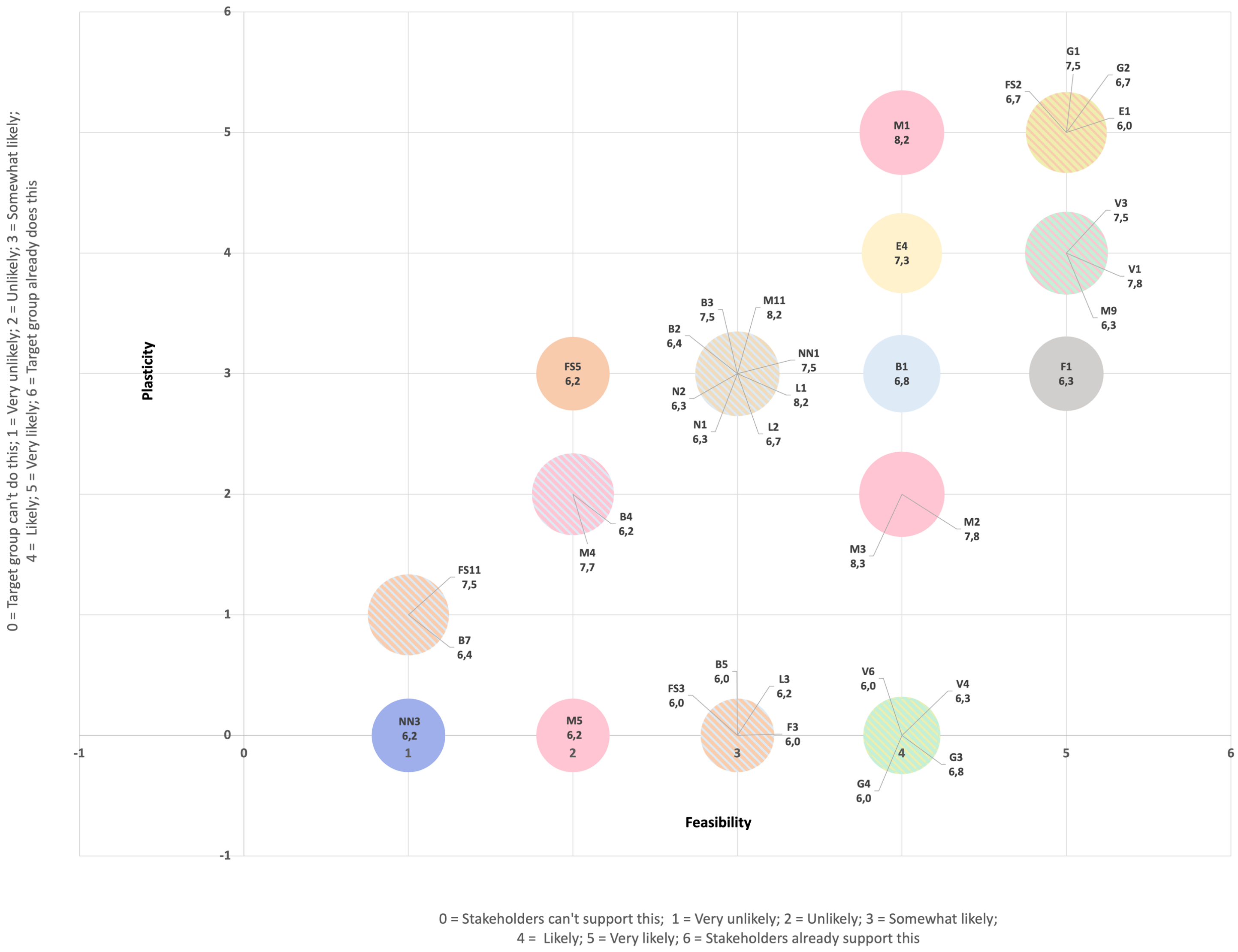

Legend

Environment and health potential score: Numbers below behaviour codes (e.g. B1) refer to the behaviour's environmental and health potential score (e.g. 6.8); score range: 0 to 10; circle size is according to the score

Beverages	
**B1**	Drink 1.5-2L water (for an adult diet) per day
**B2**	Choose tab water instead of bottled water
**B3**	Choose to drink water instead of sugar-sweetened beverages
**B4**	Choose to drink other unsweetened beverages (e.g., tea) instead of sugar-sweetened beverages
**B5**	Choose organically produced beverages (e.g., tea, coffee, juice)
**B6**	Limit alcoholic beverage consumption (max. of up to 2 glasses/per day for ♂ or 1 glass/per day for ♀
**B7**	Do not drink alcohol at an age under 18 years
**B8**	Do not drink alcohol if you are pregnant or breast feeding
Eggs	
E1	Eat 2-4 servings of eggs per week (1 serving for an adult diet: 1 egg)
Eating Behaviours	
**EB4**	Accept a variety of foods
**EB6**	Feed breast milk according to the baby's needs (if possible)
Fish	
**F1**	Eat (2-)3 servings of fish and seafood per week (1 serving for an adult diet: 125-150g)
**F3**	Choose organically produced fish
Fats, sugar, and salt	
**FS2**	Choose vegetable oils (e.g., olive oil, rapeseed oil)
**FS3**	Choose organically produced oils
**FS5**	Limit the consumption of added sugars (e.g., from sweets) (max. 25 g for an adult diet per day)
**FS11**	Limit consumption of processed foods high in salt, sugars, and fats (e.g., fast food, salty snacks, biscuits)
Grains	
**G1**	Eat 3-6 servings of grain-based foods per day (1 serv. adult diet: e.g., 40-60 g bread/60-80 g dried pasta)
**G2**	Choose a variety of grain-based foods (e.g., flour types, pasta, rice etc.)
**G3**	Choose primarily whole grains
**G4**	Choose organically produced grains
Legumes	
**L1**	Eat 3 servings of legumes per week (1 serving for an adult diet: 70 g raw/125 g cooked)
**L2**	Choose a variety of legumes
**L3**	Choose organically produced legumes
Meat	
**M1**	Eat 0-3 servings of meat per week (1 serving for an adult diet: 100-125 g)
**M2**	Limit the consumption of processed meat (both red and white meat) or even avoid it
**M3**	Limit the consumption of red meat or even avoid it
**M4**	Limit the consumption of all meats or even avoid it
**M5**	Choose poultry instead of red/processed meat
**M9**	Choose eggs instead of meat as an alternative source of protein
**M11**	Choose plant-based alternatives (e.g., legumes, nuts) instead of meat as an alternative source of protein
Nuts and seeds	
**N1**	Eat a small handful of nuts and seeds (2-)3 times a week (1 serving for an adult diet: 25 g)
**N2**	Choose a variety of nuts and seeds
Nutritional needs	
**NN1**	Know your energy (caloric) needs and eat accordingly (don't over-/under-eat)
**NN3**	Ensure an adequate vitamin-D intake through sun exposure or supplementation (20 µg/d for an adult)
Vegetables and fruits	
**V1**	Eat 5 servings of vegetables and fruits per day (1 serving for an adult diet: 125 g)
**V3**	Choose a variety of vegetables and fruits
**V4**	Choose organically produced vegetables and fruits
**V6**	Choose seasonal vegetables and fruits
Multiple colours (striped circles)	
Indicator for overlapping eating behaviours	

Comments – Please provide us here any comments you have regarding the graph(s):
